# The Synthesis of α-Aminophosphonates via Enantioselective Organocatalytic Reaction of 1-(*N*-Acylamino)alkylphosphonium Salts with Dimethyl Phosphite

**DOI:** 10.3390/molecules25020405

**Published:** 2020-01-18

**Authors:** Alicja Walęcka-Kurczyk, Krzysztof Walczak, Anna Kuźnik, Sebastian Stecko, Agnieszka Październiok-Holewa

**Affiliations:** 1 Department of Organic Chemistry, Bioorganic Chemistry and Biotechnology, Silesian University of Technology, B. Krzywoustego 4, 44-100 Gliwice, Poland; alicja.walecka-kurczyk@polsl.pl (A.W.-K.); krzysztof.walczak@polsl.pl (K.W.); anna.kuznik@polsl.pl (A.K.); 2 Biotechnology Center of Silesian University of Technology, B. Krzywoustego 8, 44-100 Gliwice, Poland; 3 Institute of Organic Chemistry, Polish Academy of Sciences, Kasprzaka 44/52, 01-224 Warsaw, Poland; sebastian.stecko@icho.edu.pl

**Keywords:** α-aminophosphonic acids, α-aminophosphonates, phosphonium salts, stereoselectivity, α-amidoalkylation, *Cinchona* alkaloids

## Abstract

α-Aminophosphonic acids are phosphorus analogues of α-amino acids. Compounds of this type find numerous applications in medicine and crop protection due to their unique biological activities. A crucial factor in these activities is the configuration of the stereoisomers. Only a few methods of stereoselective transformation of α-amino acids into their phosphorus analogues are known so far and all of them are based on asymmetric induction, thus involving the use of a chiral substrate. In contrast, we have focused our efforts on the development of an effective method for this type of transformation using a racemic mixture of starting *N*-protected α-amino acids and a chiral catalyst. Herein, a simple and efficient stereoselective organocatalytic α-amidoalkylation of dimethyl phosphite by 1-(*N*-acylamino)alkyltriphenylphosphonium salts to enantiomerically enriched α-aminophosphonates is reported. Using 5 mol% of chiral quinine- or hydroquinine-derived quaternary ammonium salts provides final products in very good yields up to 98% and with up to 92% ee. The starting phosphonium salts were easily obtained from α-amino acid derivatives by decarboxylative methoxylation and subsequent substitution with triphenylphosphonium tetrafluoroborate. The appropriate self-disproportionation of enantiomers (SDE) test for selected α-aminophosphonate derivatives via achiral flash chromatography was performed to confirm the reliability of the enantioselectivity results that were obtained.

## 1. Introduction

**Figure 1:**
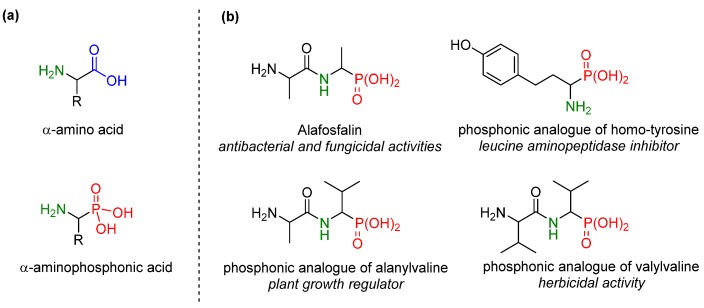
(**a**) Structure of α-amino acid and α-aminophosphonic acid; (**b**) Selected examples of biologically active phosphonic analogues of α-amino acids and their derivatives.

α-Aminophosphonic acids are one of the most recognizable classes of organophosphorus compounds. Structurally, they are analogues of α-amino acids in which the planar carboxylic group is replaced with a tetrahedral phosphonic acid group (Figure 1a). Almost 60 years of research has shown that many naturally occurring and synthetic α-aminophosphonic acids, as well as their derivatives, exhibit multidirectional, significant biological activities [1,2]. Compounds of this class are increasingly used in the synthesis of various types of peptidomimetics, clearly showing greater activity than the genuine peptides. Among the structurally diverse α-aminophosphonic acids and their derivatives, enzyme inhibitors, antibacterial and antifungal agents, anticancer agents, as well as herbicides and plant growth regulators can be found (Figure 1b) [1,2,3,4,5,6,7].

Due to the possibility of wide applications of α-aminophosphonic acids and their derivatives, various methods for their synthesis have been developed over the past several decades [5,8,9,10,11].

**Scheme 1:**
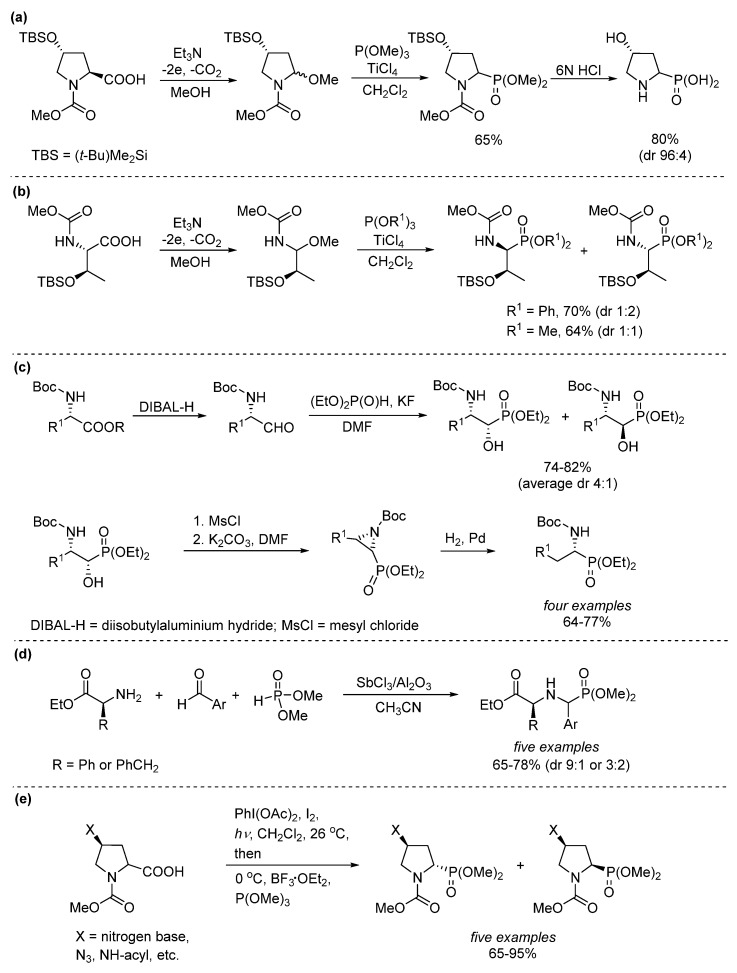
The stereoselective transformation of *N*-protected α-amino acids and their derivatives into their phosphorus analogues—known synthetic routes (**a**–**e**).

It seems natural that α-amino acids should be the most frequently used substrates for the synthesis of α-aminophosphonic acids. However, to the best of our knowledge, only a few methods for the transformation of α-amino acids into their phosphonic analogues have been reported so far [12,13,14,15,16,17,18,19,20,21,22,23,24]. Furthermore, only five of them can be classified as stereoselective processes. For example, Seebach and Renaud [12] have described a diastereoselective transformation of a *trans*-4-hydroxy-_L_-proline derivative into its phosphonic analogue (Scheme 1a). The proposed three-step path involved the oxidative decarboxylation of a proline derivative leading to 2-methoxyproline, which by the Ti-catalyzed addition of P(OMe)_3_ followed by acidic hydrolysis produced the generation of aminophosphonic acids with high dr 96:4 via a carbenium ion (Scheme 1a). Seebach et al. [14] have also used the same concept in a diastereoselective synthesis of acyclic *N*-protected phosphonodiesters, starting from *l*-threonine (Scheme 1b). However, in this case, the Michaelis-Arbuzov-type reaction exhibited no significant stereoselectivity, leading to an essentially equimolar mixture of products (Scheme 1b). In 2002, Larchevêque and Pousset [17] reported another method for the preparation of *N*-Boc-protected α-aminophosphonic acid esters with high diastereoselectivity (Scheme 1c). The crucial step in this strategy was the regioselective catalytic hydrogenation of *N*-Boc protected cis aziridines derived from syn α-hydroxy β-aminophosphonates. In 2008, Kapoor et al. [19] described the reaction of ethyl esters of (*S*)-phenylglycine or (*S*)-phenylalanine with aryl aldehydes and dimethyl phosphite in the presence of antimony trichloride adsorbed on alumina as a catalyst (Scheme 1d). The authors obtained the expected mixtures of α-aminophosphonates in good yields and a diastereomeric ratio equal to 9:1 or 3:2. In 2013, Boto et al. [24] developed a sequential radical scission-oxidation/phosphorylation protocol for the synthesis of 2-phosphonoproline derivatives (Scheme 1e). It was demonstrated that the diastereoselectivity of this reaction was highly dependent on the nitrogen substituent type at the C-4 position. For example, azanucleotide analogues were obtained with very good overall yields and diastereomeric cis/trans ratios in the range from 98:2 to 15:85.

**Scheme 2:**
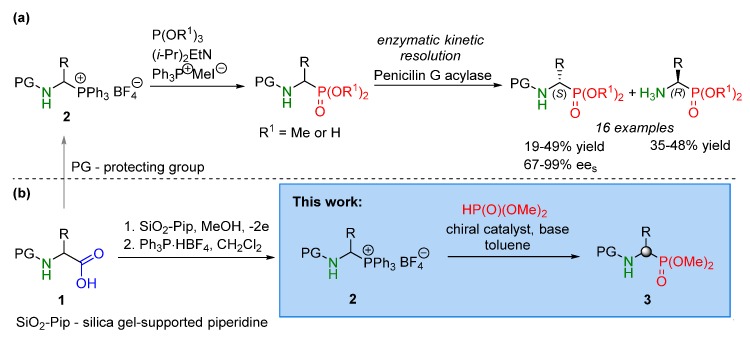
The transformation of *N*-protected α-amino acids into α-aminophosphonate derivatives via 1-(*N*-acylamino)alkylphosphonium salts: (**a**) The method based on enzymatic kinetic resolution of racemic mixtures of α-aminoalkylphosphonic acids and α-aminophosphonates; (**b**) A new approach based on enantioselective catalytic α-amidoalkylation of dimethyl phosphite with phosphonium salts.

In 2012, we described an efficient protocol for the synthesis of 1-(*N*-acylamino)alkylphosphonium salts **2** from *N*-acyl-α-amino acids **1** (Scheme 2b) [25], which may serve as attractive precursors of 1-(*N*-acylamino)alkylphosphonic and phosphinic acid esters via the Michaelis-Arbuzov-type reaction [26,27]. In an extension of these studies, in 2016, Mazurkiewicz et al. [28] targeted pure enantiomers of α-aminoalkylphosphonic acids and α-aminophosphonates by the enzymatic kinetic resolution of their racemic mixtures (Scheme 2a) in the presence of Penicillin G acylase to provide the corresponding enantiomers with high ee (enantiomeric excess) values. Of course, the maximum 50% yield of such an approach was its main limitation. So far, there are no reports on purely enantioselective methods for the preparation of enantiomerically enriched phosphonic acid esters directly from phosphonium salts in a carbon–phosphorus bond-forming reaction in the presence of an enantiodifferentiating agent.

Here, we present our recent results on the enantioselective catalytic α-amidoalkylation of dimethyl phosphite with 1-(*N*-acylamino)alkylphosphonium salts **2** in the presence of a chiral catalyst. Taking into account that the starting 1-(*N*-acylamino)alkylphosphonium tetrafluoroborates **2** are readily available from *N*-acyl-α-amino acids **1**, the presented approach can be considered a new strategy for the transformation of *N*-acyl-α-amino acids **1** into non-racemic 1-(*N*-acylamino)alkylphosphonic acid esters **3** (Scheme 2b).

## 2. Result s and Discussion

**Scheme 3:**
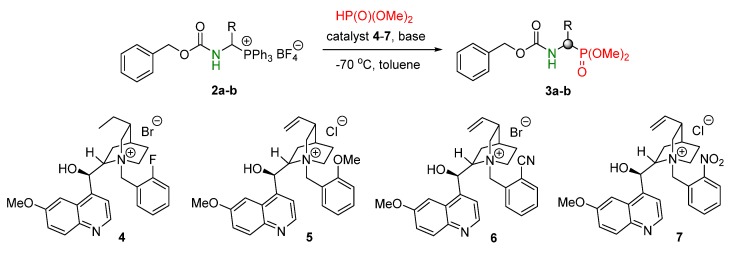
Reaction of phosphonium salts **2a-b** with dimethyl phosphite in the presence of chiral catalysts **4-7**.

At the outset of our studies, systematic exploration of different reaction conditions was conducted in order to optimize the yield and enantioselectivity. This initial screening was performed using two phosphonium salts, **2a** and **2b**, obtained from *N*-benzyloxycarbonyl alanine (Cbz-Ala-OH) and *N*-benzyloxycarbonyl phenylalanine (Cbz-Phe-OH), respectively (Scheme 3, Table 1). Four chiral quaternary ammonium salts **4**–**7**, derived from hydroquinine or quinine, were selected as stereo-differentiating agents. We decided to use this type of chiral catalysts in our reaction for several reasons. First, these *Cinchona* alkaloid derivatives can be obtained by simple and low-cost methods from commercially available substrates [29,30]. Moreover, this class of organocatalysts has emerged as a useful chiral base, as well as chiral phase transfer (PT) catalysts for the reaction of stable or in situ generated *N*-acylimines with various nucleophiles, for example carbon nucleophiles [30,31,32,33,34] and phosphorus nucleophiles, such as dialkyl phosphites [30,34,35,36,37,38,39]. Therefore, we expected the use of chiral catalysts **4**–**7** for the stereoselective α-amidoalkylation of dimethyl phosphite with phosphonium salts **2** to provide satisfactory results (Scheme 3).

Initially, salt **2a** was reacted with dimethyl phosphite in toluene, using 2-fluoro substituted *N*-benzylhydroquininium bromide **4** as the catalyst (5 mol%) and excess K_2_CO_3_ at −70°C (Table 1, entry 1). After three days, the expected dimethyl *N*-protected α-aminoethylphosphonate **3a** was obtained with a moderate yield and enantioselectivity. The same reaction carried out in the presence of 3 equiv. of KOH as a base allowed us to increase the yield of **3a** to 84% and enhanced the enantiomeric excess to 84% (Table 1, entry 2). In order to improve the solubility of phosphonium salt **2a** in the reaction mixture, a further experiment with the addition of a small amount of CH_2_Cl_2_ was performed. Unfortunately, we obtained the expected product **3a** in a lower yield and enantioselectivity (cf. Table 1, entries 2 and 3).

Next, screening of catalysts for the reaction of phosphonium salt **2a** with dimethyl phosphite was performed (Scheme 3, Table 1, entry 5 to 7). The reactions were carried out under standard conditions using 3 equiv. of KOH, 5 mol% of catalysts **5** to **7** (all bearing an ortho substituent at the benzylic quinuclidinic moiety) in toluene at −70°C (Table 1, entries 5 to 7). Among the examined catalysts, 4-F-substituted catalyst **4**, derived from hydroquinine, and 2-methoxy substituted catalyst **5**, derived from quinine, gave the best results (see Table 1, entries 2 and 5). Taking into account the strong amidoalkylating properties of phosphonium salts **2 [40,41]**, we decided to perform the next experiment with the amount of KOH decreased by half. However, this decrease in the base loading caused a drop in yield and enantioselectivity (Table 1, entry 4).

Next, phosphonium salt **2b** with a larger substituent at the α-position was subjected to the model reaction as a starting material (Scheme 3, Table 1, entries 8–15). The influence of the catalyst type and the amount of KOH on the reaction efficiency was investigated. As highlighted in Table 1, the reaction of phosphonium salt **2b** with dimethyl phosphite in toluene conducted for 3 days at −70°C in the presence of chiral catalyst **5** and KOH (3 equiv.) gave the most satisfactory results. The expected α-aminophosphonate **3b** was obtained with a high yield and good enantioselectivity (Table 1, entry 13, 84% yield, 73% ee). Additionally, in order to examine the possibility of racemization of the product under these reaction conditions, subsequent experiments were performed during a shorter time of 1 or 2 days. Based on the results obtained, we excluded the probability of such side processes (Table 1, entries 14 to 15).

Thus, we decided that the best reaction conditions for the stereoselective α-amidoalkylation of dimethyl phosphite by phosphonium salts were three equivalents of KOH and three days in toluene at the temperature of −70°C, in the presence of chiral PT catalyst **4** or **5**.

**Scheme 4:**
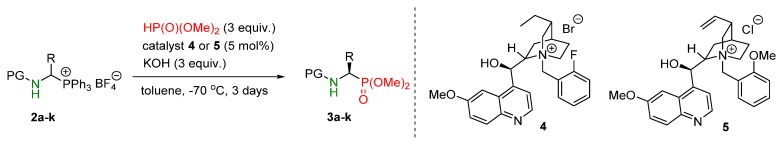
Enantioselective organocatalytic α-amidoalkylation of dimethyl phosphite with phosphonium salts **2**.

With the optimized conditions in hand, the scope of the stereoselective α-amidoalkylation of dimethyl phosphite with various phosphonium salts **2a**–**k**, catalyzed by chiral ammonium salts **4** or **5** was investigated (Scheme 4, Table 2).

Similarly as for the above mentioned reaction of *N*-Cbz-protected salt **2b** with dimethyl phosphite, the same reaction of *N*-Boc-protected phosphonium salt **2c** resulted in a very good yield of 93% and 72% ee (compare Table 2, entries 2 to 3). In the case of more sterically hindered salts, such as **2d** and **2i**–**k**, the reaction with dimethyl phosphite furnished generally good yields in the range of 69%–98% and moderate 42%–65% ee (see Table 2, entries 4, 9 to 11). Interestingly, in the case of *N*-Boc-protected valine-derivative **2e**, a significant decrease in enantioselectivity (to 23% ee) was observed (Table 2, entry 5), indicating that the steric hindrance at the β-position of the amino acid moiety could affect the transition state responsible for enantiodifferentiation. In comparison, the removal of the unfavorable steric interaction, as in the case of leucine-derived salt **2f**, led to a respectable stereoselectivity level to provide product **3f** in good yields and with ee values of 80% (with cat. **4**) and 79% (with cat. **5**), respectively (see Table 2, entry 6). To our delight, under these conditions, phosphonium salts derived from unnatural amino acids, such as norvaline **2g** and norleucine **2h**, gave the expected products **3g**,**h** with very good yields and useful enantioselectivities (see Table 2, entry 7, 85% yield, 80% ee and entry 8, 80% yield, 92% ee).

It is well known that ee values can be improved via recrystallization, which leads to the self-disproportionation of enantiomers (SDE) phenomenon [42]. Additionally, it has been demonstrated that some compounds, e.g., enantiomerically enriched α and β-amino acid derivatives [43,44] and especially β-amino-α,α-difluorophosphonic acid derivatives [45] exhibit pronounced SDE during achiral column chromatography. The occurrence of the SDE phenomenon may cause mistakes in the reporting of the ee value of catalytic asymmetric reactions. Thus, we conducted the SDE test via achiral flash chromatography in order to confirm that the values of enantiomeric excess that were obtained were reliable. After performing the appropriate test, we found that no SDE phenomenon was present in our case (for details, see Supplementary Materials).

The comparison of the optical rotation of two selected dimethyl aminophosphonate derivatives **3a** and **3f** with literature values allowed us to determine the absolute configuration of the products to be *R* (see paragraph in Materials and Methods). It has been found that phosphonic acid derivatives possessing the (*R*)-configuration, corresponding to the stereochemistry of proteinogenic _L_-α-amino acids, exhibit higher biological activities than their *S* analogues [2]. For example, the *R* enantiomer of the phosphonic acid analogue of leucine is a significantly more potent inhibitor of leucine aminopeptidase than its *S* enantiomer [46].

In conclusion, we developed a simple and effective enantioselective route for the α-amidoalkylation of dimethyl phosphite with 1-(*N*-acylamino)alkyltriphenylphosphonium tetrafluoroborates in the presence of inexpensive and easily available chiral *Cinchona* alkaloid derivatives under PTC conditions. The methodology provided constitutes a convenient approach for the synthesis of enantiomerically enriched, especially sterically unhindered dimethyl *N*-protected α-aminophosphonates, with useful ee values above 80%. Taking into account that the starting phosphonium salts are readily available from the corresponding α-amino acids, the presented procedure can be considered a new strategy for the enantioselective construction of non-racemic α-aminophosphonates from *N*-acyl-α-amino acids and sets the basis for further developments.

## 3. Materials and Methods

### 3.1. General Information

Infrared spectra (IR) were recorded on a Nicolet 6700 FT-IR spectrophotometer, Thermo Scientific, USA (ATR method). ^1^H-NMR spectra were acquired on Varian 400 at 400 MHz. Data were given as follows: chemical shift in ppm with tetramethylsilane (TMS) as the internal standard, multiplicity (s = singlet; d = doublet; t = triplet; q = quartet; br = broad; m = multiplet), coupling constant (Hz), and integration. ^13^C-NMR spectra were measured on Varian 400 at 100 MHz. Chemical shifts were reported in ppm from the solvent resonance employed as the internal standard (CDCl_3_ at 77.0 ppm). ^31^P NMR spectra were measured on a Varian 400 spectrometer at 162 MHz without the resonance shift standard, with respect to H_3_PO_4_ as zero ppm. Melting points were determined in capillaries in a Stuart Scientific SMP3 melting point apparatus and were uncorrected. The high-resolution mass spectra (HRMS) were obtained by electrospray ionization (ESI) using a Waters Corporation Xevo G2 QTOF instrument. Sonication was carried out using Elmasonic 10H laboratory ultrasonic bath (37 kHz, 30 W).

α-Amidoalkylation reactions of dimethyl phospite were performed at −70°C using Julabo ultra-low refrigerated-circulator F81-ME. For TLC analysis, Merck TLC silica gel 60 F254 plates were used. The plates were visualized by UV light (254 nm) and/or dipped in a solution of cerium sulfate and tetrahydrate of ammonium heptamolybdate in H_2_SO_4aq_ and heated. Kieselgel 60 (Merck, 0.040–0.063 mm) was used for column chromatography. The enantiomeric excess (ee) of the products was determinated by HPLC using Hitachi, LaChrom Elite, HPLC Chromatograph, Tokyo, Japan (Diode Array L-2450 detector) or Knauer HPLC Chromatograph, Berlin, Germany (UV WellChrom K-2501 detector, RI WellChrom K-2301 detector) using Daicel Chiralpak IA, AD-H, OD-H column. Optical rotations were measured on a JASCO V-650 polarimeter and reported as follows: ${\left[\alpha \right]}_{D}^{T}$ (c in g/100 mL solvent).

Materials. All solvents and common reagents were obtained from commercial suppliers. Dimethyl phosphite was purchased from Alfa Aesar. 3-(1-Piperidino)propyl-functionalized silica gel (SiO2-Pip) was purchased from Sigma-Aldrich. 

Racemic samples rac **3a**–**k** were obtained using tetrabutylammonium bromide as the catalyst. The chiral catalysts **4** [36], **5** [32], **6** [31], and **7** [33] were obtained following literature procedures.

### 3.2. Substrate Synthesis

The starting phosphonium salts **2a**–**k** were synthesized by a previously described two-step protocol [25], which consists of electrochemical decarboxylative α-methoxylation of *N*-acyl-α-amino acids (Step 1) and transformation of the resulting *N*-(1-methoxyalkyl)carbamates to 1-(*N*-acylamino)alkyltriphenylphosphonium tetrafluoroborates **2** (Step 2). The analytical data and spectra of compounds **2a**–**f,j,k** were identical to those previously described [25,41]. For previously unknown phosphonium salts **2g**–**i**, analytical and spectroscopic data are reported below.

#### General Procedure for the Synthesis of 1-(*N*-acylamino)alkyltriphenylphosphonium Tetrafluoroborates 2

Step 1: Methanol (30 cm^3^), *N*-acyl-α-amino acid (3.0 mmol), and SiO_2_-Pip (200 mg, 0.22 mmol) were added to an undivided cylindrical glass electrolyzer (85 cm^3^) with a thermostatic jacket, equipped with a magnetic stirrer, a cylindrical Pt mesh anode (47 cm^2^), and a similar cathode (44 cm^2^), all arranged concentrically to one another at a distance of 2.5 ± 0.5 mm. The electrolysis was conducted while stirring at a current density of 0.3 A/dm^2^ at 10°C until 3.5–3.75 F/mol charge had passed [3.5 F/mol for *N*-Cbz-valine and 3.75 F/mol for other *N*-protected α-amino acids]. When the process of electrolysis was finished, SiO_2_-Pip was filtered out and methanol was evaporated under reduced pressure to afford the corresponding *N*-(1-methoxyalkyl)carbamates. Crude *N*-(1-methoxyalkyl)carbamates were used in the next step without purification.

Step 2: A mixture of the corresponding *N*-(1-methoxyalkyl)carbamates and Ph_3_P∙HBF_4_ (0.98 equiv.) in CH_2_Cl_2_ (2 cm^3^) was stirred at 25°C for 30 min and then the solvent was removed under reduced pressure. The crude 1-(*N*-acylamino)alkyltriphenylphosphonium salts **2g**–**i** were used in the reaction with dimethyl phosphite without further purification. Recrystallization from the mixture of CH_2_Cl_2_/Et_2_O failed.

*1-(N-Benzyloxycarbonylamino)butyltriphenylphosphonium tetrafluoroborate* (**2g**). White solid; 96% yield (1.60 g); mp 110°C to 111°C. ^1^H-NMR (400 MHz, CDCl_3_): δ 7.88–7.60 (m, 15H), 7.35–7.20 (m, 5H), 6.97 (br d, *J* = 9.2 Hz, 1H), 5.54–5.46 (m, 1H), 4.99 (d, *J* = 12.4 Hz, 1H), 4.89 (d, *J* = 12.4 Hz, 1H), 2.33–2.21 (m, 1H), 1.75–1.56 (m, 2H), 1.56–1.43 (m, 1H), 0.92 (t, *J* = 7.2 Hz, 3H). ^13^C-NMR (100 MHz, CDCl_3_): δ 156.6 (d, *J* = 3.0 Hz), 135.9, 135.1 (d, *J* = 2.9 Hz), 134.1 (d, *J* = 9.3 Hz), 130.3 (d, *J* = 12.1 Hz), 128.4, 127.9, 127.8, 116.9 (d, *J* = 80.7 Hz), 67.3, 50.6 (d, *J* = 53.1 Hz), 32.9 (d, *J* = 5.4 Hz), 19.7 (d, *J* = 13.0 Hz), 13.1 (d, *J* = 0.7 Hz). ^31^P NMR (162 MHz, CDCl_3_): δ 25.5. IR (ATR): 3321, 2964, 1716, 1521, 1438, 1234, 1051 cm^−1^. HMRS (ESI) *m*/*z*: calcd for C_30_H_31_NO_2_P [M^+^] 468.2092, found 468.2092.

*1-(N-Benzyloxycarbonylamino)pentyltriphenylphosphonium tetrafluoroborate* (**2h**). White solid; 92% yield (1.57 g); mp 114°C. ^1^H-NMR (400 MHz, CDCl_3_): δ 7.78–7.59 (m, 15H), 7.32–7.27 (m, 3H), 7.24–7.20 (m, 2H), 6.95 (br d, *J* = 9.2 Hz, 1H), 5.51–5.43 (m, 1H), 4.99 (d, *J* = 12.4 Hz, 1H), 4.89 (d, *J* = 12.4 Hz, 1H), 2.29–2.17 (m, 1H), 1.72–1.58 (m, 2H), 1.49–1.23 (m, 3H), 0.82 (t, *J* = 7.2 Hz, 3H). ^13^C-NMR (100 MHz, CDCl_3_): δ 156.6 (d, *J* = 3.0 Hz), 136.0, 135.1 (d, *J* = 3.0 Hz), 134.1 (d, *J* = 9.2 Hz), 130.4 (d, *J* = 12.3 Hz), 128.4, 127.9, 127.8, 116.9 (d, *J* = 80.7 Hz), 67.3, 50.9 (d, *J* = 52.6 Hz), 30.8 (d, *J* = 5.5 Hz), 28.5 (d, *J* = 12.5 Hz), 21.7 (d, *J* = 1.0 Hz), 13.7. ^31^P NMR (162 MHz, CDCl_3_): δ 25.5. IR (ATR): 3320, 2960, 1716, 1519, 1438, 1236, 1051. HMRS (ESI) *m*/*z*: calcd for C_31_H_33_NO_2_P [M^+^] 482.2249, found 482.2254.

*1-(N-Benzyloxycarbonylamino)cyclohexylmethyltriphenylphosphonium tetrafluoroborate* (**2i**). White solid; 90% yield (1.61 g); mp 119 °C to 120 °C. ^1^H-NMR (400 MHz, CDCl_3_): δ 7.80–7.67 (m, 9H), 7.65–7.56 (m, 6H), 7.31–7.25 (m, 3H), 7.17–7.12 (m, 2H), 7.00 (br d, *J* = 8.8 Hz, 1H), 5.34 (q, *J* = 8.9 Hz, 1H), 4.84 (d, *J* = 12.4 Hz, 1H), 4.75 (d, *J* = 12.4 Hz, 1H), 2.39–2.26 (m, 1H), 1.80–1.30 (m, 6H), 1.22–1.06 (m, 3H), 0.89–0.75 (m, 1H). ^13^C-NMR (100 MHz, CDCl_3_): δ 156.3 (d, *J* = 1.8 Hz), 135.7, 134.6 (d, *J* = 3.0 Hz), 134.5 (d, *J* = 9.2 Hz), 130.0 (d, *J* = 12.2 Hz), 128.4, 127.9, 127.8, 118.4 (d, *J* = 79.5 Hz), 67.1, 55.4 (d, *J* = 45.9 Hz) 38.8 (d, *J* = 5.8 Hz), 32.3 (d, *J* = 2.9 Hz), 29.6 (d, *J* = 7.9 Hz), 25.6 (d, *J* = 11.2 Hz), 25.6. ^31^P NMR (162 MHz, CDCl_3_): δ 27.4. IR (ATR): 3339, 2932, 1713, 1519, 1438, 1233, 1051 cm^−1^. HMRS (ESI) *m*/*z*: calcd for C_33_H_35_NO_2_P [M^+^] 508.2400, found 508.2410.

### 3.3. General Procedure for the PT-Catalyzed Reaction of 1-(N-acylamino)alkyltriphenylphosphonium Salts 2 with Dimethyl Phosphite 

1-(*N*-Acylamino)alkyltriphenylphosphonium salt **2** (0.2 mmol), catalyst **4** or **5** (0.01 mmol), and toluene (2 mL) were placed in a test tube. The mixture was sonicated in an ultrasonic bath for 10 min and dimethyl phosphite (55 μL, 66 mg, 0.6 mmol) was added. After cooling the resulting mixture to −70 °C, solid KOH (34 mg, 0.6 mmol) was added. After vigorously stirring at the same temperature (−70 °C) for 3 days, the reaction mixture was treated with saturated NH_4_Cl (4 to 5 mL). The mixture was allowed to warm to room temperature and then extracted three times with CH_2_Cl_2_. The organic layers were combined, dried over anhydrous Na_2_SO_4_, and concentrated under reduced pressure. The residue was purified by flash column chromatography on silica gel using mixtures of hexane/EtOAc/acetone (5:3:2) as the eluent.

*(R)-Benzyl N-[1-(dimethoxyphosphoryl)ethyl]carbamate* (**3a**) [36]. Colorless oil; 84% yield (48.4 mg). The enantiomeric excess (84% ee for catalyst **4**) was determined by HPLC using Chiralpak AD-H column (*n*-hexane/isopropanol = 90/10, flow rate 0.75 mL/min, λ = 210 nm) t (major) = 17.0 min, t (minor) = 20.8 min. ${\left[\alpha \right]}_{D}^{24}$ = −15 (CHCl_3_, c = 0.5). ^1^H-NMR (400 MHz, CDCl_3_): δ 7.38–7.31 (m, 5H), 5.23 (br d, *J* = 9.6 Hz, 1H), 5.12 (s, 2H), 4.25–4.14 (m, 1H), 3.76 (d, *J* = 10.8 Hz, 3H), 3.73 (d, *J* = 10.8 Hz, 3H), 1.39 (dd, *J*_1_ = 16.6 Hz, *J_2_* = 7.4 Hz, 3H). ^13^C-NMR (100 MHz, CDCl_3_): δ 155.6 (d, *J* = 6.0 Hz), 136.2, 128.4, 128.1, 128.0, 67.1, 53.2 (d, *J* = 7.0 Hz) 53.0 (d, *J* = 6.0 Hz), 42.8 (d, *J* = 158.0 Hz), 15.8. ^31^P NMR (162 MHz, CDCl_3_): δ 27.9. IR (ATR): 3239, 2955, 1714. 1537, 1225, 1022 cm^−1^.

The absolute configuration of compound **3a** was determined to be *R* by comparison of its optical rotation with a literature value:

Measured optical rotation (84% ee): ${\left[\alpha \right]}_{D}^{24}$ = −15 (CHCl_3_, c = 0.5)

Literature data: ${\left[\alpha \right]}_{D}^{20}$ = -17.5 (CHCl_3_, c = 1.0) for the *(R)-*isomer [47].

*(R)-Benzyl N-[1-(dimethoxyphosphoryl)-2-phenylethyl]carbamate* (**3b**) [48] Colorless oil; 84% yield (60.9 mg). The enantiomeric excess (73% ee for the catalyst **4**) was determined by HPLC using Chiralpak AD-H column (*n*-hexane/isopropanol = 85:15, flow rate 0.8 mL/min, λ = 210 nm) t (major) = 20.6 min, t (minor) = 28.7 min. ${\left[\alpha \right]}_{D}^{24}$ = −28 (CHCl_3_, c = 0.5). ^1^H-NMR (400 MHz, CDCl_3_): δ 7.34–7.20 (m, 10H), 5.05 (br d, *J* = 10.0 Hz, 1H), 5.00 (s, 2H), 4.48–4.37 (m, 1H), 3.74 (d, *J* = 10.4 Hz, 3H), 3.70 (d, *J* = 10.8 Hz, 3H), 3.22 (ddd, *J_1_* = 14.0 Hz, *J_2_* = 8.8 Hz, *J_3_* = 4.7 Hz, 1H), 2.87 (dt, *J_1_* = 20.2 Hz, *J_2_* = 10.0 Hz, 1H). ^13^C-NMR (100 MHz, CDCl_3_): δ 155.7 (d, *J* = 5.4 Hz), 136.4, 136.2, 129.2, 128.4, 128.4, 128.0, 127.8, 126.8, 67.0, 53.2 (d, *J* = 7.1 Hz), 53.0 (d, *J* = 6.6 Hz), 48.2 (d, *J* = 155.9 Hz) 35.8 (d, *J* = 3.6 Hz).^31^P NMR (162 MHz, CDCl_3_): δ 26.4. IR (ATR): 3223, 2954, 1708, 1542, 1255, 1222, 1028 cm^−1^.

*(R)-tert-Butyl N-[1-(dimethoxyphosphoryl)-2-phenylethyl]carbamate* (**3c**) [36]. White solid; 93% yield (61.1 mg); mp 77 °C to 79 °C. The enantiomeric excess (72% ee for the catalyst **4**) was determined by HPLC using Chiralpak AD-H column (*n*-hexane/isopropanol = 85:15, flow rate 0.5 mL/min, λ = 210 nm) t (major) = 26.4 min, t (minor) = 19.1 min. ${\left[\alpha \right]}_{D}^{25}$ = −21 (CHCl_3_, c = 0.5). Compound **3c** exists as a 85:15 mixture of rotamers at 25 °C in CDCl_3_. The spectra for the major rotamer are as follows: ^1^H-NMR (400 MHz, CDCl_3_): δ 7.31–7.20 (m, 5H), 4.70 (br d, *J* = 10.0 Hz, 1H), 4.44–4.33 (m, 1H), 3.75 (d, *J* = 10.4 Hz, 6H), 3.21 (ddd, *J_1_* = 13.6 Hz, *J_2_* = 8.8 Hz, *J_3_* = 4.8 Hz, 1H), 2.84 (dd, *J_1_* = 14.4 Hz, *J_2_* = 10.2 Hz, 1H), 1.32 (s, 9H). ^13^C-NMR (100 MHz, CDCl_3_): δ 154.9 (d, *J* = 7.0 Hz), 136.5 (d, *J* = 13.1 Hz), 129.2, 128.3, 126.7, 80.0, 53.2 (d, *J* = 7.1 Hz), 52.9 (d, *J* = 6.7 Hz), 47.5 (d, *J* = 155.6 Hz) 36.0 (d, *J* = 3.8 Hz), 28.1.^31^P NMR (162 MHz, CDCl_3_): δ 32.1. IR (ATR): 3255, 2981, 1706, 1525, 1237, 1155, 1043 cm^−1^.

*(R)-Benzyl N-[1-(dimethoxyphosphoryl)-2-methylpropyl]carbamate* (**3d**) [49]. Colorless oil; 85% yield (53.7 mg). The enantiomeric excess (56% ee for the catalyst **4**) was determined by HPLC using Chiralpak AD-H column (*n*-hexane/isopropanol = 90:10, flow rate 0.75 mL/min, λ = 210 nm) t (major) = 14.5 min, t (minor) = 23.0 min. ${\left[\alpha \right]}_{D}^{24}$ = −5 (CHCl_3_, c = 0.5). ^1^H-NMR (400 MHz, CDCl_3_): δ 7.37–7.31 (m, 5H), 5.16 (d, *J* = 12.0 Hz, 1H), 5.11 (d, *J* = 12.4 Hz, 1H), 5.08 (br d, *J* = 9.6 Hz, 1H), 4.05 (ddd, *J*_1_ = 18.6 Hz, *J*_2_ = 10.4 Hz, *J*_3_ = 4.2 Hz, 1H)_,_ 3.75 (d, *J* = 10.8 Hz, 3H), 3.71 (d, *J* = 10.8 Hz, 3H), 2.25–2.15 (m, 1H), 1.03 (d, *J* = 6.8 Hz, 3H), 1.00 (d, *J* = 6.8 Hz, 3H). ^13^C-NMR (100 MHz, CDCl_3_): δ 156.3 (d, *J* = 6.4 Hz), 136.2, 128.5, 128.2, 128.0, 67.2, 52.9 (d, *J* = 6.3 Hz), 52.8 (d, *J* = 7.2 Hz), 52.2 (d, *J* = 151.8 Hz), 28.9 (d, *J* = 4.4 Hz), 20.3 (d, *J* = 12.6 Hz), 17.7 (d, *J* = 4.1 Hz).^31^P NMR (162 MHz, CDCl_3_): δ 26.7. IR (ATR) 3239, 2958, 1710, 1533, 1289, 1228, 1024 cm^−1^.

*(R)-tert-Butyl N-[1-(dimethoxyphosphoryl)-2-methylpropyl]carbamate* (**3e**) [50]. White solid; 88% yield (49.4 mg); mp 83°C to 84°C. The enantiomeric excess (23% ee for the catalyst **4**) of the product **3e** was determined by HPLC, after transformation into its Cbz derivative **3e’** through Boc deprotection followed by CbzCl derivatization as described by Ricci et al. [36], using Chiralpak IA column (*n*-hexane/isopropanol = 90:10, flow rate 0.75 mL/min, λ = 210 nm) t (major) = 13.3 min, t (minor) = 19.8 min. ^1^H-NMR (400 MHz, CDCl_3_): δ 4.76 (br d, *J* = 9.6 Hz, 1H), 3.99 (ddd, *J*_1_ = 18.4 Hz, *J*_2_ = 10.8 Hz, *J*_3_ = 4.4 Hz, 1H), 3.76 (d, *J* = 10.8 Hz, 6H), 2.22–2.10 (m, 1H), 1.45 (s, 9H), 1.01 (dd, *J*_1_ = 6.8 Hz, *J*_2_ = 1.2 Hz, 3H), 0.99 (d, *J* = 7.2 Hz, 3H). ^13^C-NMR (100 MHz, CDCl_3_): δ 155.6 (d, *J* = 6.3 Hz), 80.0, 52.9 (d, *J* =7.2 Hz), 52.7 (d, *J* = 6.7 Hz), 51.5 (d, *J* = 151.4 Hz), 28.9 (d, *J* = 4.8 Hz), 28.2, 20.3 (d, *J* = 12.6 Hz), 17.8 (d, *J* = 4.2 Hz). ^31^P NMR (162 MHz, CDCl_3_): δ 27.2. IR (ATR): 3267, 2972, 1701, 1530, 1293, 1233, 1163, 1035 cm^−1^.

*(R)-Benzyl N-[1-(dimethoxyphosphoryl)-3-methylbutyl]carbamate* (**3f**) [36]. Colorless oil; 82% yield (54.1 mg). The enantiomeric excess (80% ee for the catalyst **4**) was determined by HPLC using Chiralpak IA column (*n*-hexane/isopropanol = 85/15, flow rate 0.75 mL/min, λ = 254 nm) t (major) = 10.1 min, t (minor) = 13.4 min. ${\left[\alpha \right]}_{D}^{25}$ = −32 (CHCl_3_, c = 1.0). ^1^H-NMR (400 MHz, CDCl_3_): δ 7.37–7.28 (m, 5H), 5.16 (d, *J* = 12.4 Hz, 1H), 5.08 (d, *J* = 12.4 Hz, 2H), 4.25–4.14 (m, 1H), 3.75 (d, *J* = 10.4 Hz, 3H), 3.70 (d, *J* = 10.4 Hz, 3H), 1.79–1.68 (m, 1H), 1.58 (q, *J* = 7.2 Hz, 2H), 0.94 (d, *J* = 2.4 Hz, 3H), 0.92 (d, *J* = 2.0 Hz, 3H). ^13^C-NMR (100 MHz, CDCl_3_): δ 155.9 (d, *J* = 4.6 Hz), 136.3, 128.4, 128.1, 127.9, 67.0, 53.1 (d, *J* = 7.1 Hz), 52.9 (d, *J* = 6.5 Hz), 45.5 (d, *J* = 155.6 Hz), 38.3 (d, *J* = 2.4 Hz), 24.3 (d, *J* = 13.2 Hz), 23.2, 21.0. ^31^P NMR (162 MHz, CDCl_3_): δ 29.0. IR (ATR): 3274, 2955, 1686, 1541, 1267, 1233, 1027 cm^−1^.

The absolute configuration of compound **3f** was determined to be *R* by comparison of its optical rotation with a literature value:

Measured optical rotation (80% ee): ${\left[\alpha \right]}_{D}^{25}$ = −32 (CHCl_3_, c = 1.0)

Literature data: ${\left[\alpha \right]}_{D}^{20}$ = −36.4 (CHCl_3_, c = 1.0) for the *(R)-*isomer [51].

*(R)-Benzyl N-[1-(dimethoxyphosphoryl)butyl]carbamate* (**3g**). Colorless oil; 85% yield (53.4 mg). The enantiomeric excess (80% ee for the catalyst **4**) was determined by HPLC using Chiralpak AD-H column (*n*-hexane/isopropanol = 90/10, flow rate 0.75 mL/min, λ = 210 nm) t (major) = 18.1 min, t (minor) = 24.9 min. ${\left[\alpha \right]}_{D}^{25}$ = −25 (CHCl_3_, c = 0.5). ^1^H-NMR (400 MHz, CDCl_3_): δ 7.38–7.30 (m, 5H), 5.15 (d, *J* = 12.4 Hz, 1H), 5.10 (d, *J* = 12.4 Hz, 1H), 4.93 (br d, *J* = 10.4 Hz, 1H), 4.17–4.07 (m, 1H), 3.76 (d, *J* = 10.8 Hz, 3H), 3.72 (d, *J* = 10.4 Hz, 3H), 1.87–1.80 (m, 1H), 1.63–1.33 (m, 3H), 0.93 (t, *J* = 7.2 Hz, 3H). ^13^C-NMR (100 MHz, CDCl_3_): δ 156.0 (d, *J* = 5.5 Hz), 136.2, 128.5, 128.2, 128.0, 67.2, 53.1 (d, *J* = 7.2 Hz), 53.0 (d, *J* = 6.5 Hz), 46.9 (d, *J* = 155.0 Hz), 31.8 (d, *J* = 2.4 Hz), 19.0 (d, *J* = 12.7 Hz), 13.5. ^31^P NMR (162 MHz, CDCl_3_): δ 27.5. IR (ATR): 3239, 2957, 1714, 1537, 1226, 1025 cm^−1^. HMRS (ESI) *m*/*z*: calcd for C_14_H_22_NO_5_NaP [M+Na]^+^ 338.1133, found 338.1135.

*(R)-Benzyl N-[1-(dimethoxyphosphoryl)pentyl]carbamate* (**3h**) [52]. Colorless oil; 80% yield (53.0 mg). The enantiomeric excess (92% ee for the catalyst **4**) was determined by HPLC using Chiralpak AD-H column (*n*-hexane/isopropanol = 90/10, flow rate 0.75 mL/min, λ = 210 nm) t (major) = 15.7 min, t (minor) = 23.2 min. ${\left[\alpha \right]}_{D}^{24}$ = −23 (CHCl_3_, c = 0.5).^1^H-NMR (400 MHz, CDCl_3_): δ 7.36–7.29 (m, 5H), 5.15 (d, *J* = 12.0 Hz, 1H), 5.11 (d, *J* = 12.4 Hz, 1H), 5.02 (br d, *J* = 10.4 Hz, 1H), 4.15–4.05 (m, 1H), 3.75 (d, *J* = 10.8 Hz, 3H), 3.72 (d, *J* = 10.4 Hz, 3H), 1.87–1.80 (m, 1H), 1.62–1.26 (m, 5H), 0.89 (t, *J* = 7.0 Hz, 3H). ^13^C-NMR (100 MHz, CDCl_3_): δ 156.0 (d, *J* = 5.7 Hz), 136.2, 128.5, 128.1, 128.0, 67.1, 53.1 (d, *J* = 7.2 Hz), 52.9 (d, *J* = 6.5 Hz), 47.2 (d, *J* = 155.0 Hz), 29.5 (d, *J* = 2.7 Hz), 27.8 (d, *J* = 12.4 Hz), 22.1, 13.8. ^31^P NMR (162 MHz, CDCl_3_): δ 27.4. IR (ATR): 3242, 2955, 1715, 1537, 1223, 1026 cm^−1^.

*(R)-Benzyl N-[cyclohexyl(dimethoxyphosphoryl)methyl]carbamate* (**3i**) [36]. White solid; 71% yield (50.6 mg); mp 61.5°C to 62.5°C. The enantiomeric excess (65% ee for the catalyst **4**) was determined by HPLC using Chiralpak IA column (*n*-hexane/isopropanol = 85:15, flow rate 0.75 mL/min, λ = 205 nm) t (major) = 15.6 min, t (minor) = 20.9 min. ${\left[\alpha \right]}_{D}^{24}$ = −3 (CHCl_3_, c = 0.5). ^1^H-NMR (400 MHz, CDCl_3_): δ 7.37–7.30 (m, 5H), 5.16 (d, *J* = 12.4 Hz, 1H), 5.10 (d, *J* = 12.0 Hz, 1H), 5.02 (br d, *J* = 10.4 Hz, 1H), 4.04 (ddd, *J*_1_ = 18.7 Hz, *J*_2_ = 10.8 Hz, *J*_3_ = 4.3 Hz, 1H), 3.75 (d, *J* = 10.4 Hz, 3H), 3.70 (d, *J* = 10.8 Hz, 3H), 1.89–1.63 (m, 6H), 1.33–1.00 (m, 5H). ^13^C-NMR (100 MHz, CDCl_3_): δ 156.2 (d, *J* = 6.4 Hz), 136.2, 128.5, 128.2, 128.0, 67.2, 52.9 (d, *J* = 6.5 Hz), 52.8 (d, *J* = 7.2 Hz), 52.0 (d, *J* = 151.3 Hz), 38.6 (d, *J* = 4.2 Hz), 30.5 (d, *J* = 11.4 Hz), 28.0 (d, *J* = 4.3 Hz), 26.1 (d, *J* = 1.7 Hz), 25.9 (d, *J* = 10.9 Hz). ^31^P NMR (162 MHz, CDCl_3_): δ 26.7. IR (ATR): 3243, 2927, 1715, 1545, 1219, 1030 cm^−1^.

*(R)-Benzyl N-[2-(tert-butoxy)-1-(dimethoxyphosphoryl)ethyl]carbamate* (**3j**). White solid; 98% yield (70.6 mg); mp 67°C to 69°C. The enantiomeric excess (53% ee for the catalyst **4**) was determined by HPLC using Chiralpak AD-H column (*n*-hexane/isopropanol = 90:10 flow rate 0.75 mL/min, λ = 210 nm) t (major) = 18.8 min, t (minor) = 22.3 min. ${\left[\alpha \right]}_{D}^{25}$ = 3 (CHCl_3_, c = 0.5). ^1^H-NMR (400 MHz, CDCl_3_): δ 7.37–7.30 (m, 5H), 5.30 (br d, *J* = 9.6 Hz, 1H), 5.13 (s, 2H), 4.33–4.21 (m, 1H), 3.79–3.74 (m, 1H), 3.76 (d, *J* = 10.8 Hz, 6H), 3.58 (ddd, *J*_1_ = 28.0 Hz, *J*_2_ = 9.4 Hz, *J*_3_ = 3.8 Hz, 1H), 1.18 (s, 9H). ^13^C-NMR (100 MHz, CDCl_3_): 155.8 (d, *J* = 6.4 Hz), 136.2, 128.5, 128.2, 128.1, 73.7, 67.2, 60.6, 53.3 (d, *J* = 5.9 Hz), 52.7 (d, *J* = 6.4 Hz), 48.3 (d, *J* = 155.5 Hz), 27.3. ^31^P NMR (162 MHz, CDCl_3_): δ 26.1. IR (ATR): 3229, 2971, 1706, 1551, 1277, 1223, 1183, 1033 cm^−1^. HMRS (ESI) *m*/*z*: calcd for C_16_H_26_NO_6_NaP [M+Na]^+^ 382.1395, found 382.1398.

*(R)-tert-Butyl N-[2-(benzyloxy)-1-(dimethoxyphosphoryl)ethyl]carbamate* (**3k**). Colorless oil; 84% yield (60.2 mg). The enantiomeric excess (53% ee for the catalyst **5**) was determined by HPLC using Chiralpak OD-H column (*n*-hexane/isopropanol = 97:3, flow rate 0.5 mL/min, λ = 212 nm) t (major) = 54.1 min, t (minor) = 62.0 min. ${\left[\alpha \right]}_{D}^{25}$ = 2 (CHCl_3_, c = 1.0). ^1^H-NMR (400 MHz, CDCl_3_): δ 7.35–7.27 (m, 5H), 5.06 (br d, *J* = 9.2 Hz, 1H), 4.56 (s, 2H), 4.36–4.24 (m, 1H), 3.85–3.79 (m, 1H), 3.75 (d, *J* = 10.4 Hz, 3H), 3.75 (d, *J* = 10.4 Hz, 3H), 3.70 (ddd, *J*_1_ = 24.0 Hz, *J*_2_ = 9.8 Hz, *J*_3_ = 3.8 Hz 1H), 1.45 (s, 9H). ^13^C-NMR (100 MHz, CDCl_3_): 155.1 (d, *J* = 7.4 Hz), 137.6, 128.4, 127.8, 127.7, 80.2, 73.3, 68.8 (d, *J* = 2.3 Hz), 53.3 (d, *J* = 6.3 Hz), 52.8 (d, *J* = 6.3 Hz), 47.4 (d, *J* = 155.0 Hz), 28.3. ^31^P NMR (162 MHz, CDCl_3_): δ 25.7. IR (ATR): 3263, 2956, 1708, 1497, 1365, 1242, 1165, 1026 cm^−1^. HMRS (ESI) *m*/*z*: calcd for C_16_H_26_NO_6_NaP [M+Na]^+^ 382.1395, found 382.1390.

## Figures and Tables

**Table 1:** Screening of the reaction conditions for PT-catalyzed stereoselective α-amidoalkylation reaction ^1^.

Entry	Salt	R	Catalyst (5 mol%)	Base (equiv.)	Time [days]	Yield ^2^ [%]	ee ^3^ [%]
1	**2a**	Me	**4**	K_2_CO_3_ (3)	3	62	49
**2**	**2a**	**Me**	**4**	**KOH (3)**	**3**	**84**	**84**
3 ^4^	**2a**	Me	**4**	KOH (3)	3	71	59
4	**2a**	Me	**4**	KOH (1.5)	3	64	78
**5**	**2a**	**Me**	**5**	**KOH (3)**	**3**	**84**	**83**
6	**2a**	Me	**6**	KOH (3)	3	75	81
7	**2a**	Me	**7**	KOH (3)	3	82	72
8	**2b**	PhCH_2_	**4**	KOH (3)	3	87	65
9	**2b**	PhCH_2_	**4**	KOH (3)	1	78	68
10	**2b**	PhCH_2_	**4**	KOH (1.5)	3	47 ^5^	78
11	**2b**	PhCH_2_	**4**	KOH (2)	1	70	73
12	**2b**	PhCH_2_	**4**	KOH (1.5)	1	63	70
**13**	**2b**	**PhCH_2_**	**5**	**KOH (3)**	**3**	**84**	**73**
14	**2b**	PhCH_2_	**5**	KOH (3)	2	79	70
15	**2b**	PhCH_2_	**5**	KOH (3)	1	71	74

^1^ Reaction conditions: Phosphonium salt **2** (0.2 mmol), dimethyl phosphite (0.6 mmol, 3 equiv.), catalyst **4**–**7** (0.01 mmol, 5 mol%), toluene (2 mL), −70 °C. ^2^ Isolated yield. ^3^ The enantiomeric excess (ee) was determined by HPLC using a column with a chiral stationary phase. ^4^ The reaction was carried out in a mixture of toluene/CH_2_Cl_2_ 9:1 (2 mL) ^5^ The reaction mixture additionally contained benzyl *N*-(1-hydroxy-2-phenylethyl)carbamate.

**Table 2:** Substrate scope of the stereoselective reaction of phosphonium salts **2** with dimethyl phosphite ^1^.

Entry	Product 3		Catalyst	Yield ^2^ [%]	ee ^3^ [%]
1	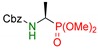	**3a**	**4**	84	84
			**5**	84	83
2	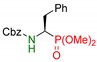	**3b**	**4**	84	73
3	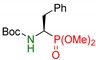	**3c**	**4**	93	72
			**5**	73	61
4	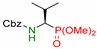	**3d**	**4**	82	56
5	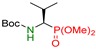	**3e**	**4**	88	23 ^4^
6	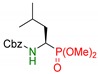	**3f**	**4**	82	80
			**5**	60	79
7	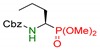	**3g**	**4**	85	80
8	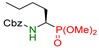	**3h**	**4**	80	92
9	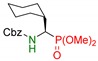	**3i**	**4**	79	65
			**5**	69	55
10	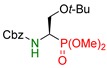	**3j**	**4**	98	53
			**5**	88	54
11	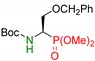	**3k**	**4**	69	42
			**5**	84	53

^1^ The reaction was carried out with phosphonium salt **2** (0.2 mmol) and dimethyl phosphite (0.6 mmol, 3 equiv.) in the presence of catalyst **4** or **5** (0.01 mmol, 5 mol%) and KOH (0.6 mmol, 3 equiv.) in toluene (2 mL), at −70 °C. ^2^ Isolated yield. ^3^ The enantiomeric excess (ee) was determined by HPLC using a column with a chiral stationary phase. ^4^ Determined after Boc deprotection and Cbz derivatisation.

## Supplementary Materials

The following are available online. Supporting information includes ^1^H-, ^13^C-, ^31^P-NMR, HRMS spectra of all new compounds **2**, **3**, and HPLC data for compounds **3** and results of the SDE test.
