# Supplementary material for: The Synthesis of α-Aminophosphonates via Enantioselective Organocatalytic Reaction of 1-(N-Acylamino)alkylphosphonium Salts with Dimethyl Phosphite
Source: Molecules. 2020 Jan 18;25(2):405. doi: 10.3390/molecules25020405 (PMC7024258; doi:10.3390/molecules25020405)

molecules

MDPI

(Article)

The Synthesis of α-Aminophosphonates via Enantioselective Organocatalytic Reaction of 1-(*N*-Acylamino)alkylphosphonium Salts with Dimethyl Phosphite

Alicja Walęcka-Kurczyk [1,2], Krzysztof Walczak[1], Anna Kuźnik [1,2], Sebastian Stecko [3] and Agnieszka Październiok-Holewa [1,2,*]

[1] Department of Organic Chemistry, Bioorganic Chemistry and Biotechnology, Silesian University of Technology, B. Krzywoustego 4, 44-100 Gliwice, Poland; alicja.walecka-kurczyk@polsl.pl, krzysztof.walczak@polsl.pl, anna.kuznik@polsl.pl

[2] Biotechnology Center of Silesian University of Technology, B. Krzywoustego 8, 44-100 Gliwice, Poland

[3] Institute of Organic Chemistry, Polish Academy of Sciences, Kasprzaka 44/52, 01-224 Warsaw, Poland, sebastian.stecko@icho.edu.pl

* Correspondence: agnieszka.pazdzierniok-holewa@polsl.pl; Tel.: +48 032-237-2218; fax: +48 032-237-2094

Supporting information

Table of contents

1. ^{1}H NMR, ^{13}C NMR, ^{31}P NMR and HRMS of 1-(*N*-acylamino)alkyltriphenyl-phosphonium tetrafluorobrates **2g-i**……………………………………………………..…..S2
2. ^{1}H NMR, ^{13}C NMR, ^{31}P NMR and HRMS of *N*-[1-(dimethoxyphosphoryl)-alkyl]carbamates **3**…………………………………………………………………....…..S14
3. HPLC data of *N*-[1-(dimethoxyphosphoryl)alkyl]carbamates **3**…………..……….........S50
4. Results of the SDE test via achiral flash chromatography...S80

^1H NMR [400 MHz/$CDCl_3$/TMS]: 1-(*N*-Benzyloxycarbonylamino)butyltriphenylphosphonium tetrafluoroborate (**2g**)

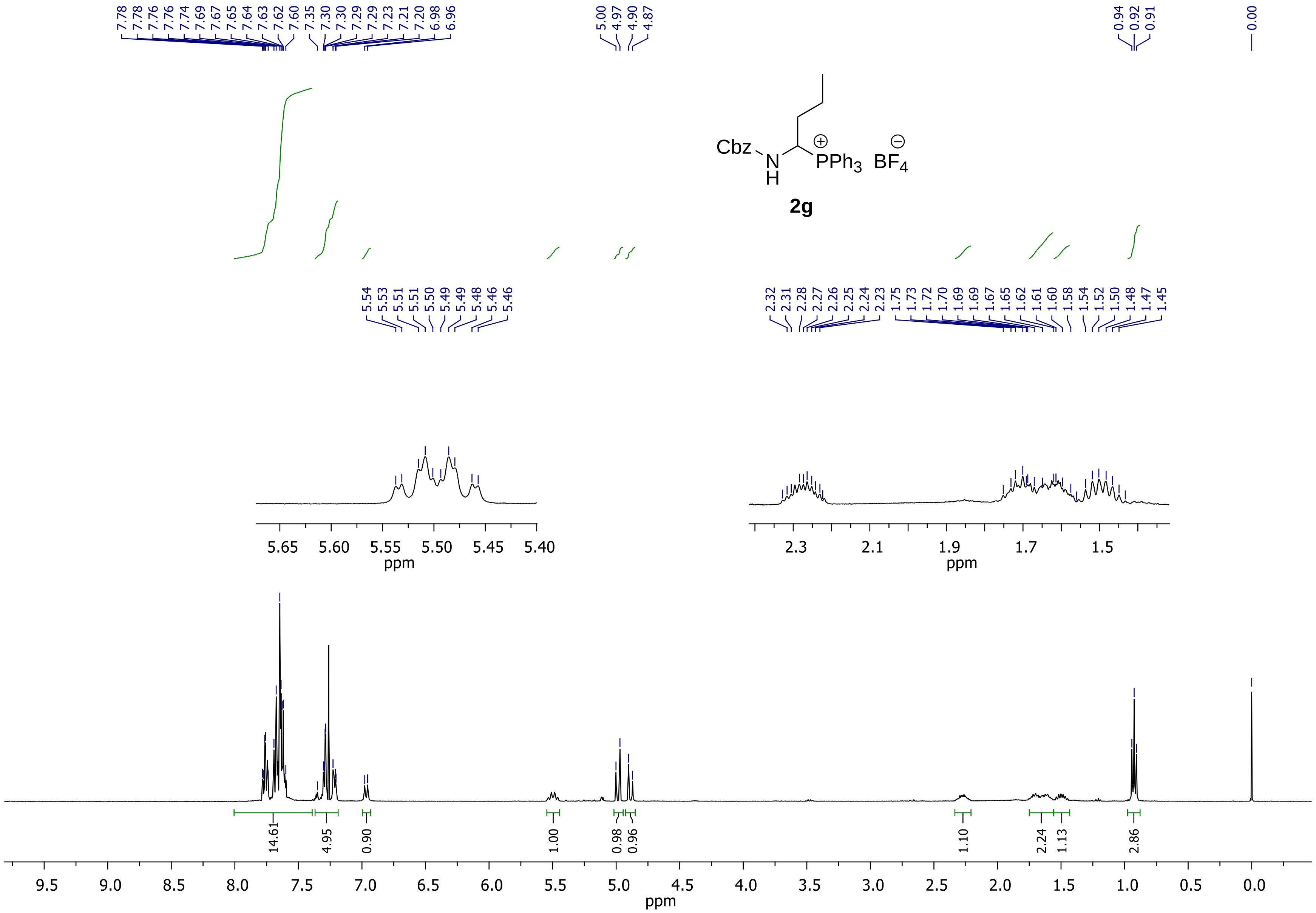

^{13}C NMR [100 MHz/$CDCl_3$]: 1-(*N*-Benzyloxycarbonylamino)butyltriphenylphosphonium tetrafluoroborate (**2g**)

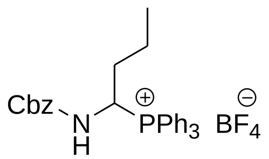

2g

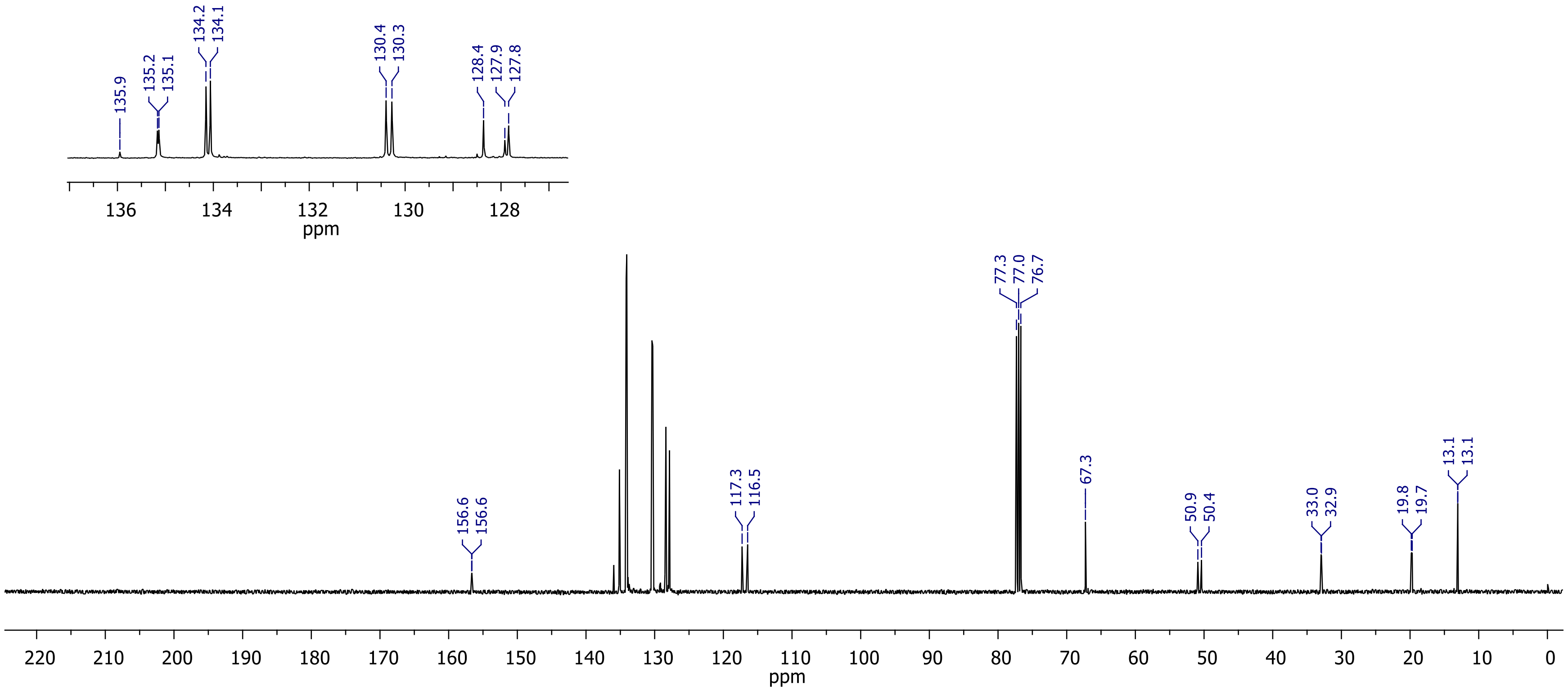

^{31}P NMR [162 MHz/$CDCl_3$]: 1-(*N*-Benzyloxycarbonylamino)butyltriphenylphosphonium tetrafluoroborate (**2g**)

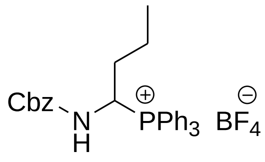

2g

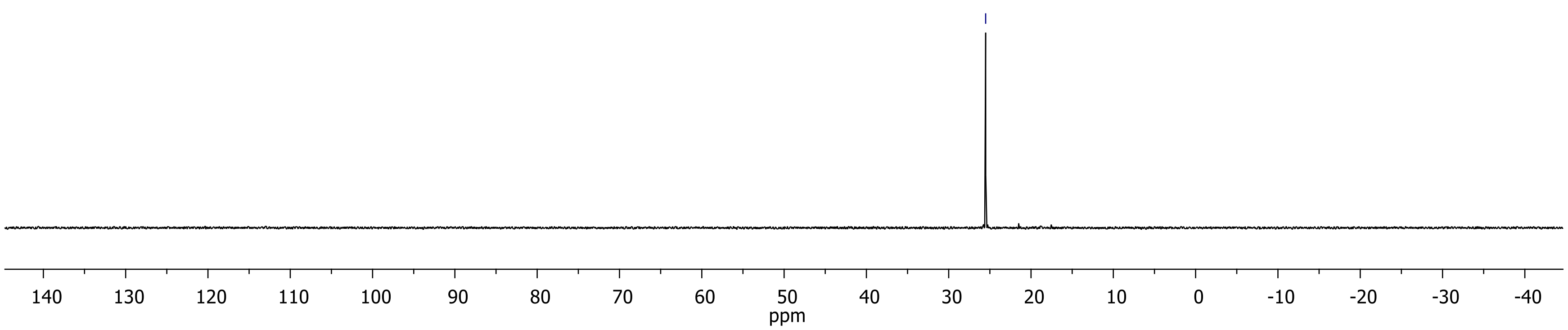

HRMS: 1-(*N*-Benzyloxycarbonylamino)butyltriphenylphosphonium tetrafluoroborate (**2g**)

Tolerance = 10.0 PPM / DBE: min = -20.0, max = 100.0
Element prediction: Off
Number of isotope peaks used for i-FIT = 2
Monoisotopic Mass, Even Electron Ions
52 formula(e) evaluated with 1 results within limits (up to 5 closest results for each mass)
Elements Used:

Mass	RA	Calc. Mass	mDa	PPM	DBE	Formula	i-FIT	i-FIT Norm	Fit Conf %	C	H	N	O	P
468.2092	100.00	468.2092	0.0	0.0	16.5	C30 H31 N O2 P	338.5	n/a	n/a	30	31	1	2	1

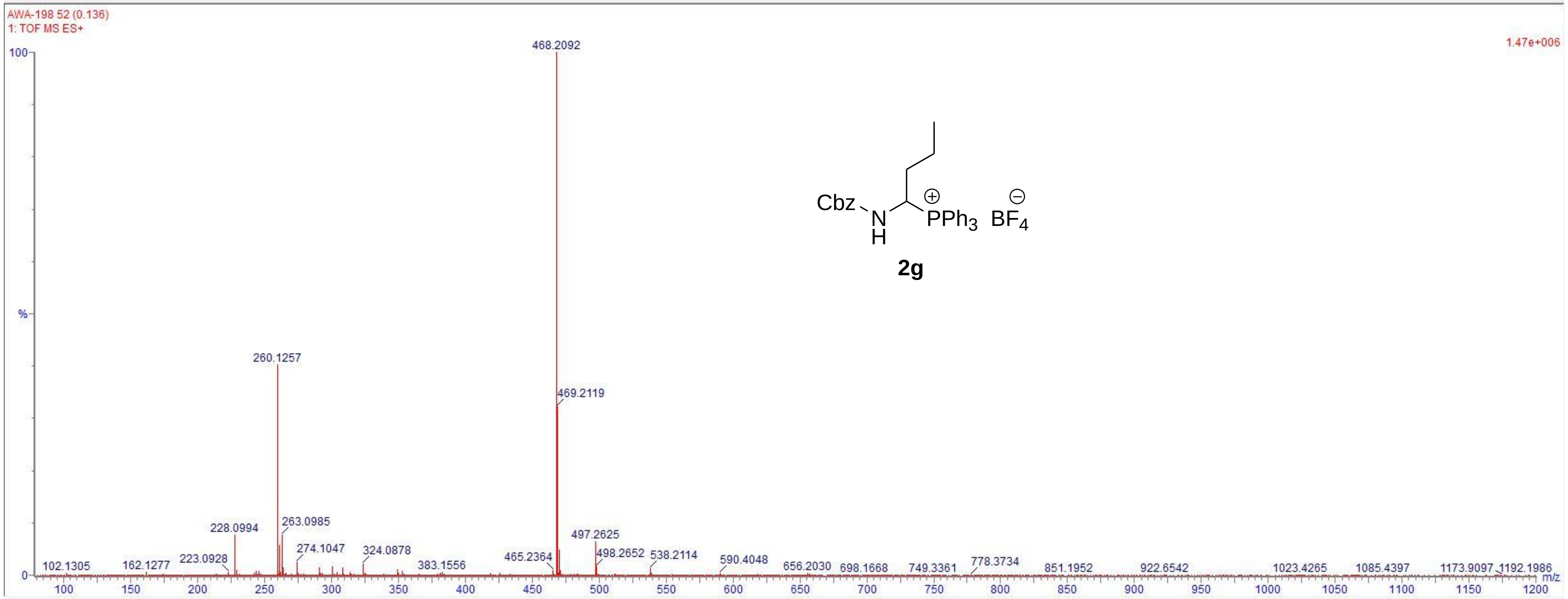

^{1}H NMR [400 MHz/$CDCl_3$/TMS]: 1-(*N*-Benzyloxycarbonylamino)pentyltriphenylphosphonium tetrafluoroborate (**2h**)

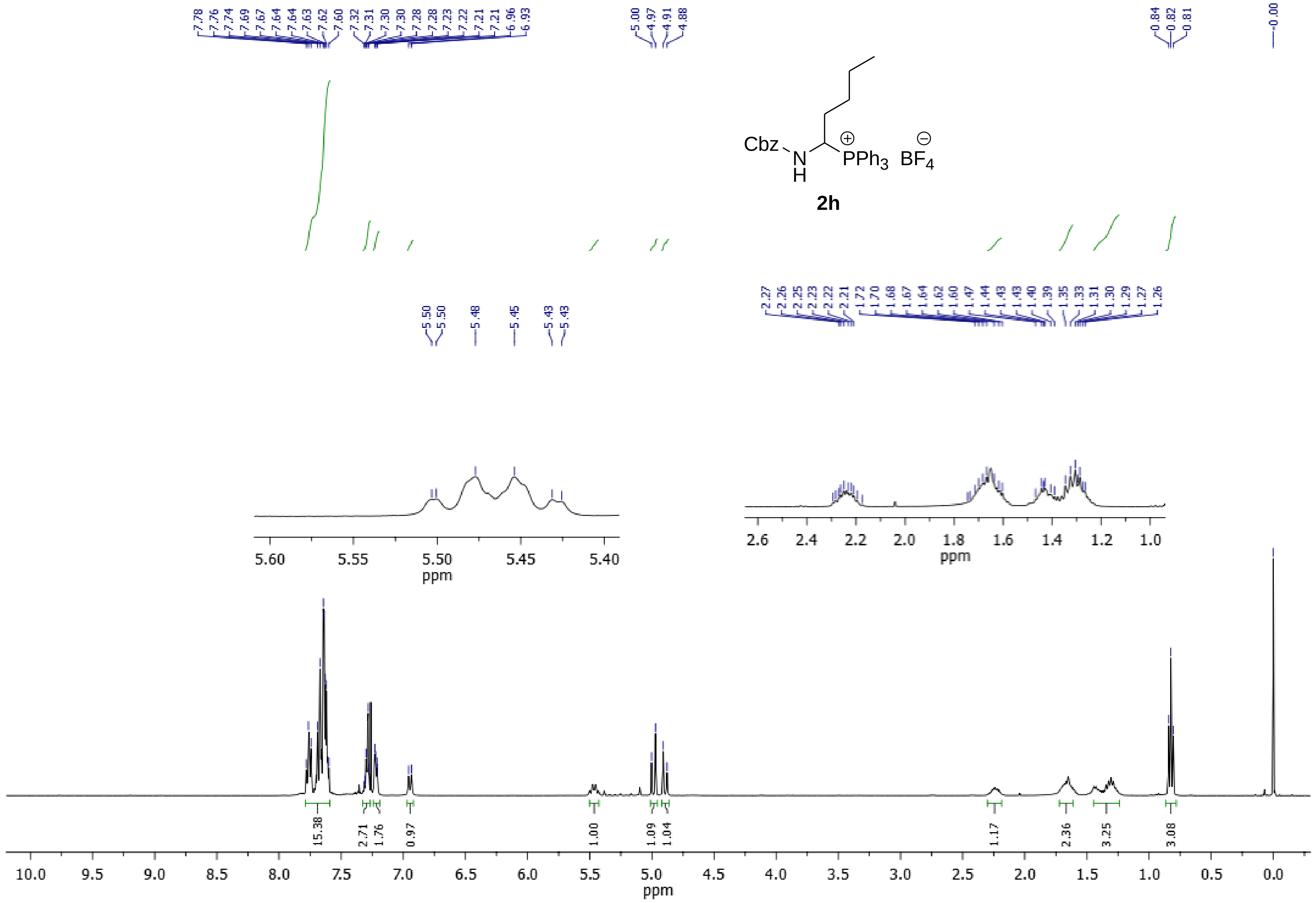

^{13}C NMR [100 MHz/$CDCl_3$]: 1-(*N*-Benzyloxycarbonylamino)pentyltriphenylphosphonium tetrafluoroborate (**2h**)

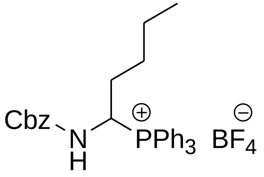

2h

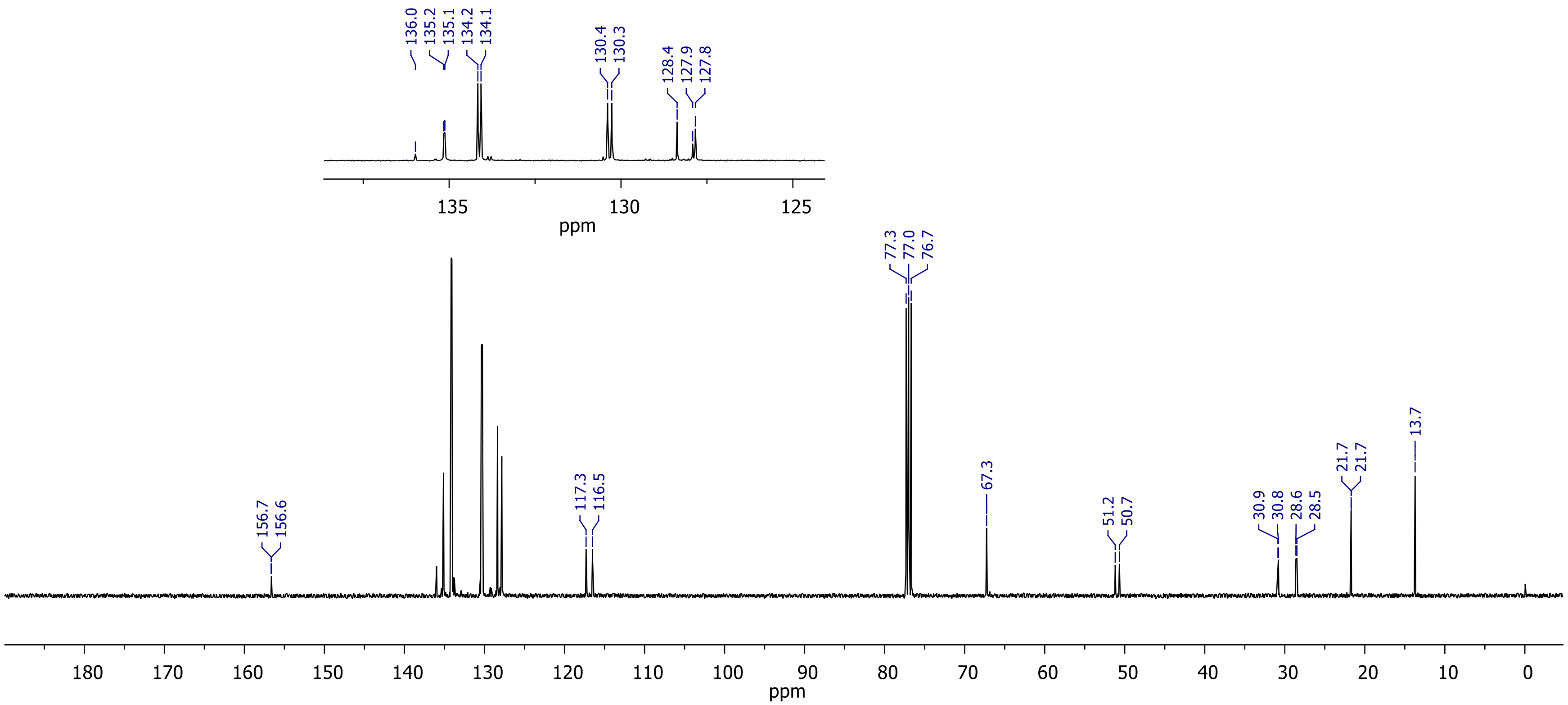

^{31}P NMR [162 MHz/$CDCl_3$]: 1-(*N*-Benzyloxycarbonylamino)pentyltriphenylphosphonium tetrafluoroborate (**2h**)

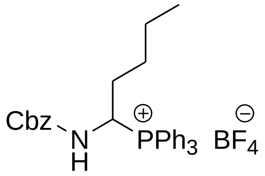

2h

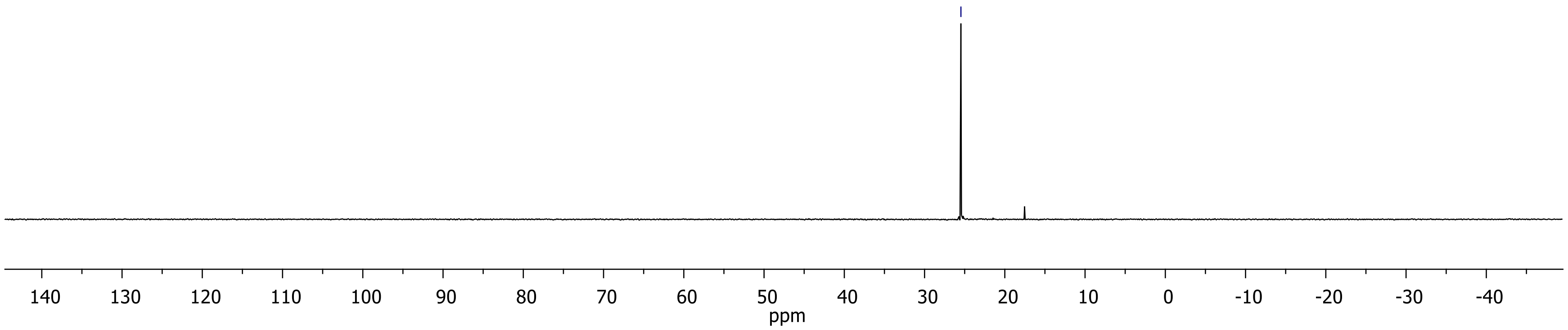

HRMS: 1-(*N*-Benzyloxycarbonylamino)pentyltriphenylphosphonium tetrafluoroborate (**2h**)

Tolerance = 10.0 PPM / DBE: min = -20.0, max = 100.0
Element prediction: Off
Number of isotope peaks used for i-FIT = 2
Monoisotopic Mass, Even Electron Ions
56 formula(e) evaluated with 1 results within limits (up to 5 closest results for each mass)
Elements Used:

Mass	RA	Calc. Mass	mDa	PPM	DBE	Formula	i-FIT	i-FIT Norm	Fit Conf %	C	H	N	O	P
482.2254	100.00	482.2249	0.5	1.0	16.5	C31 H33 N O2 P	529.5	n/a	n/a	31	33	1	2	1

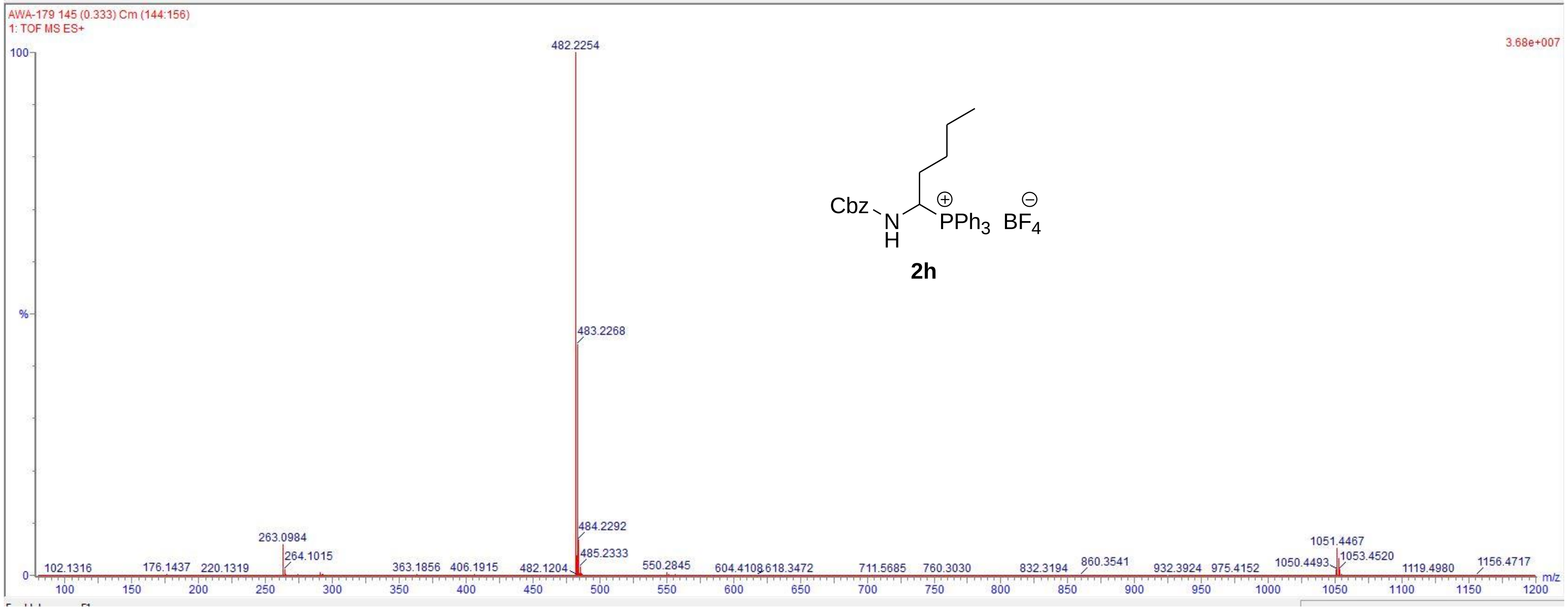

^{1}H NMR [400 MHz/$CDCl_3$/TMS]: 1-(*N*-Benzyloxycarbonylamino)cyclohexylmethyltriphenylphosphonium tetrafluoroborate (**2i**)

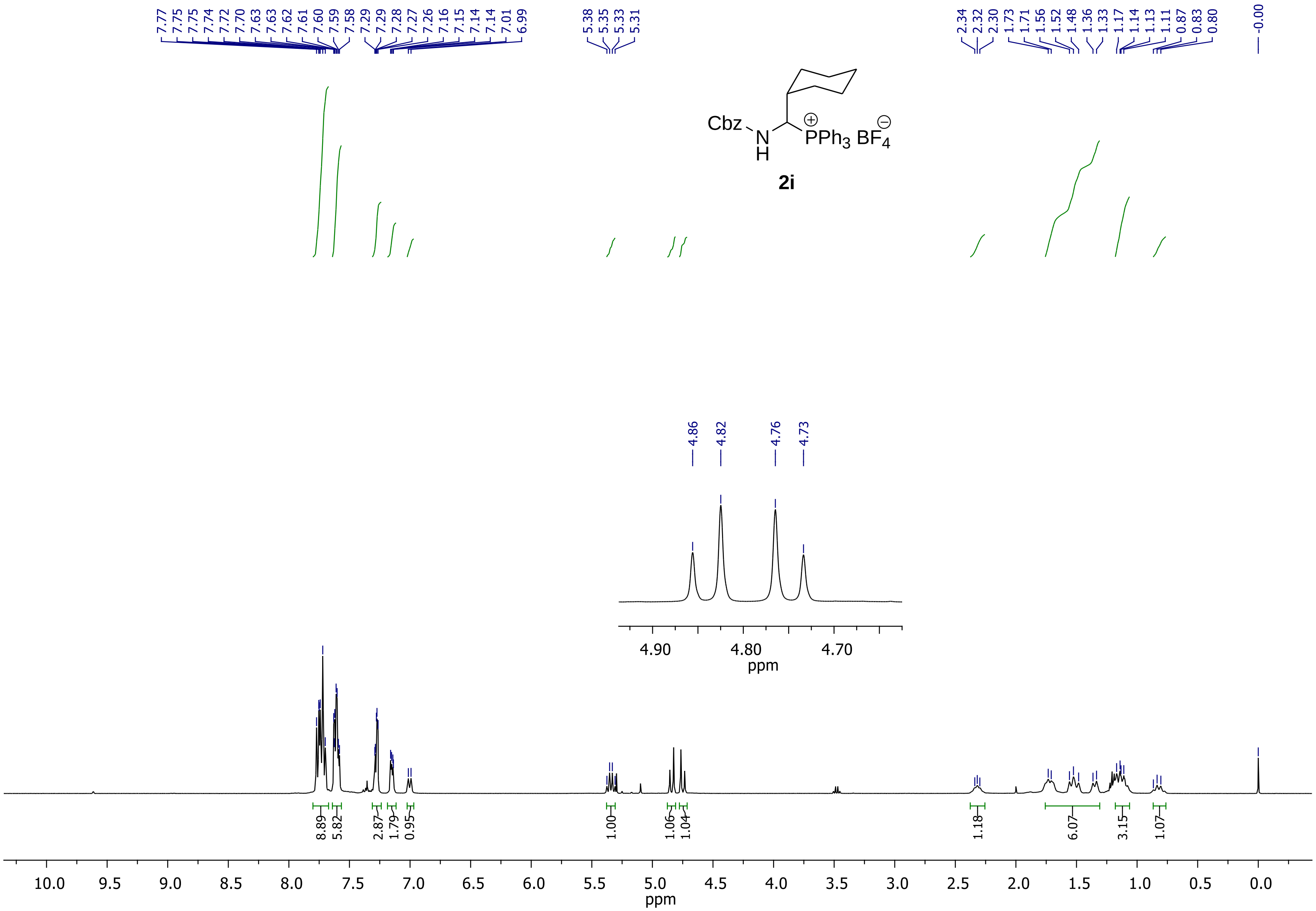

^{13}C NMR [100 MHz/$CDCl_3$]: 1-(*N*-Benzyloxycarbonylamino)cyclohexylmethyltriphenylphosphonium tetrafloroborate (**2i**)

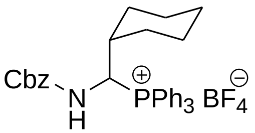

2i

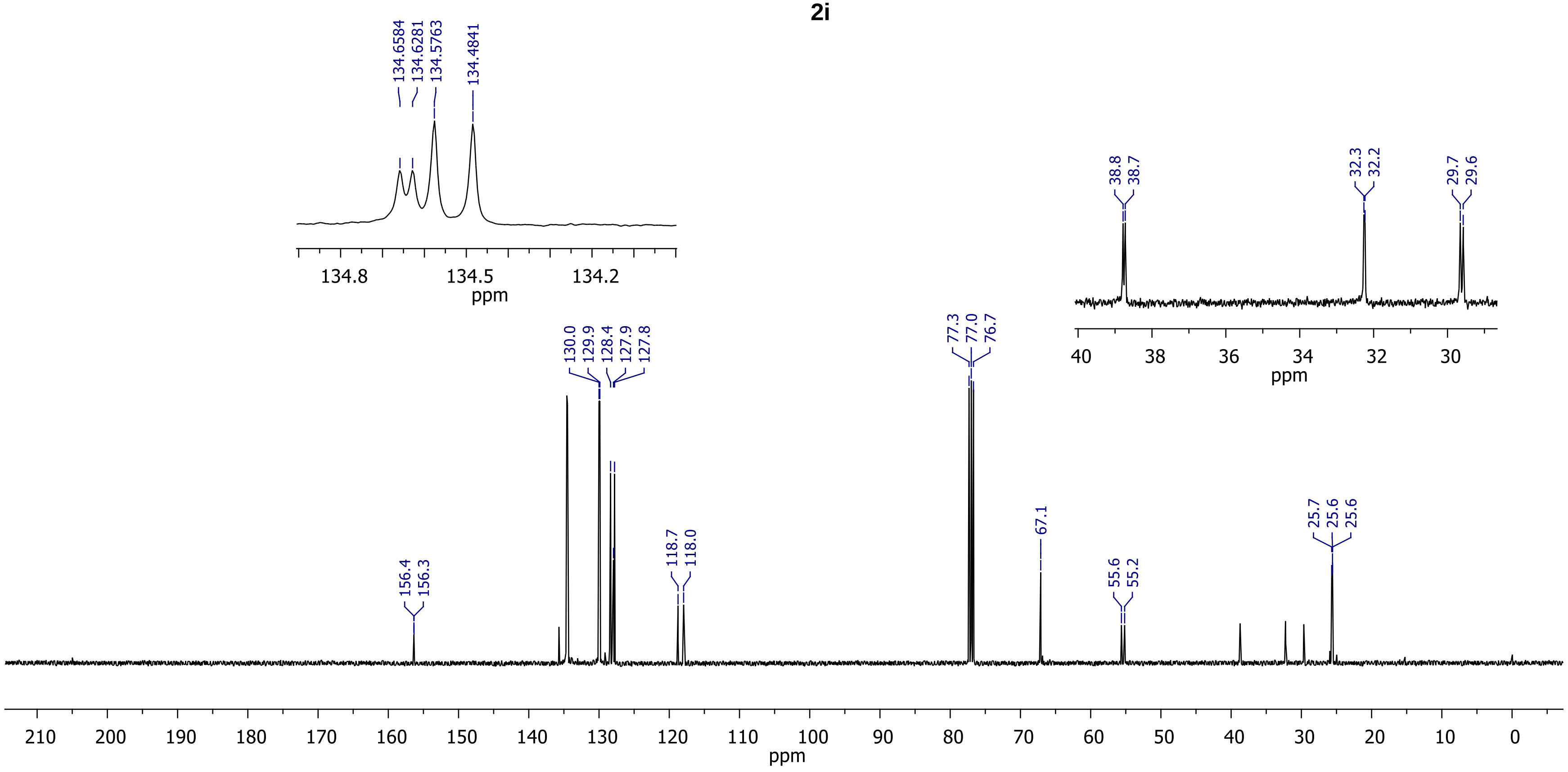

^{31}P NMR [162 MHz/$CDCl_3$]: 1-(*N*-Benzyloxycarbonylamino)cyclohexylmethyltriphenylphsphonium tetrafloroborate (**2i**)

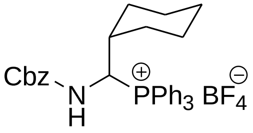

2i

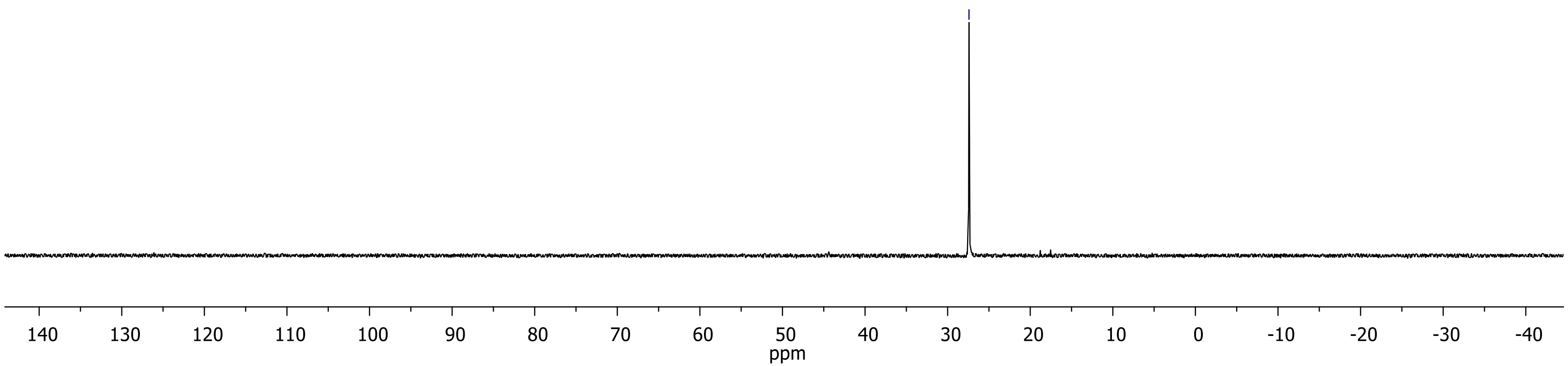

HRMS: 1-(*N*-Benzyloxycarbonylamino)cyclohexylmethyltriphenylphsphonium tetrafloroborate (**2i**)

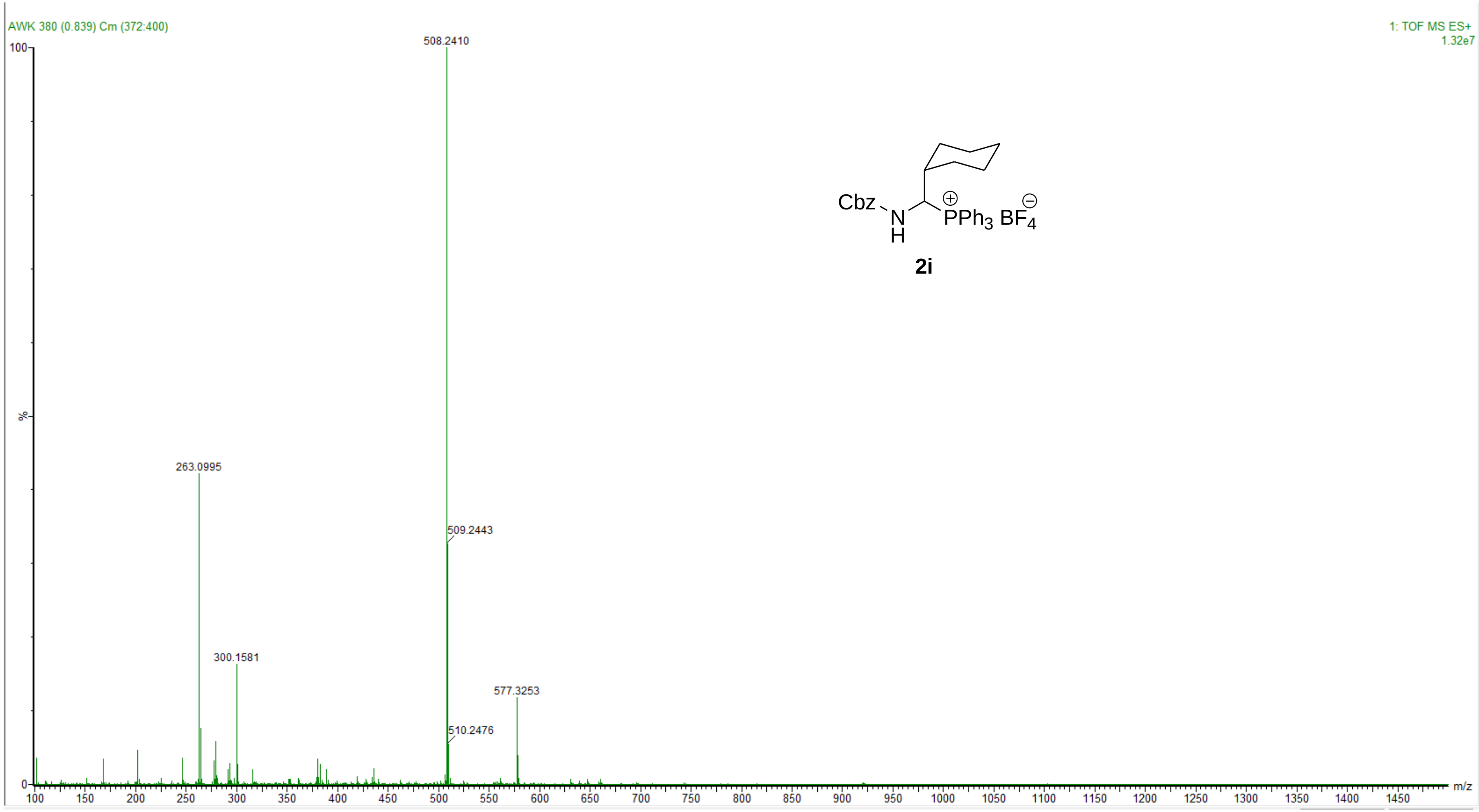

^{1}H NMR [400 MHz/$CDCl_3$/TMS]: (*R*)-Benzyl *N*-[1-(dimethoxyphosphoryl)ethyl]carbamate (**3a**)

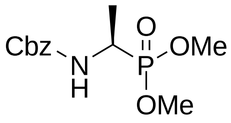

3a

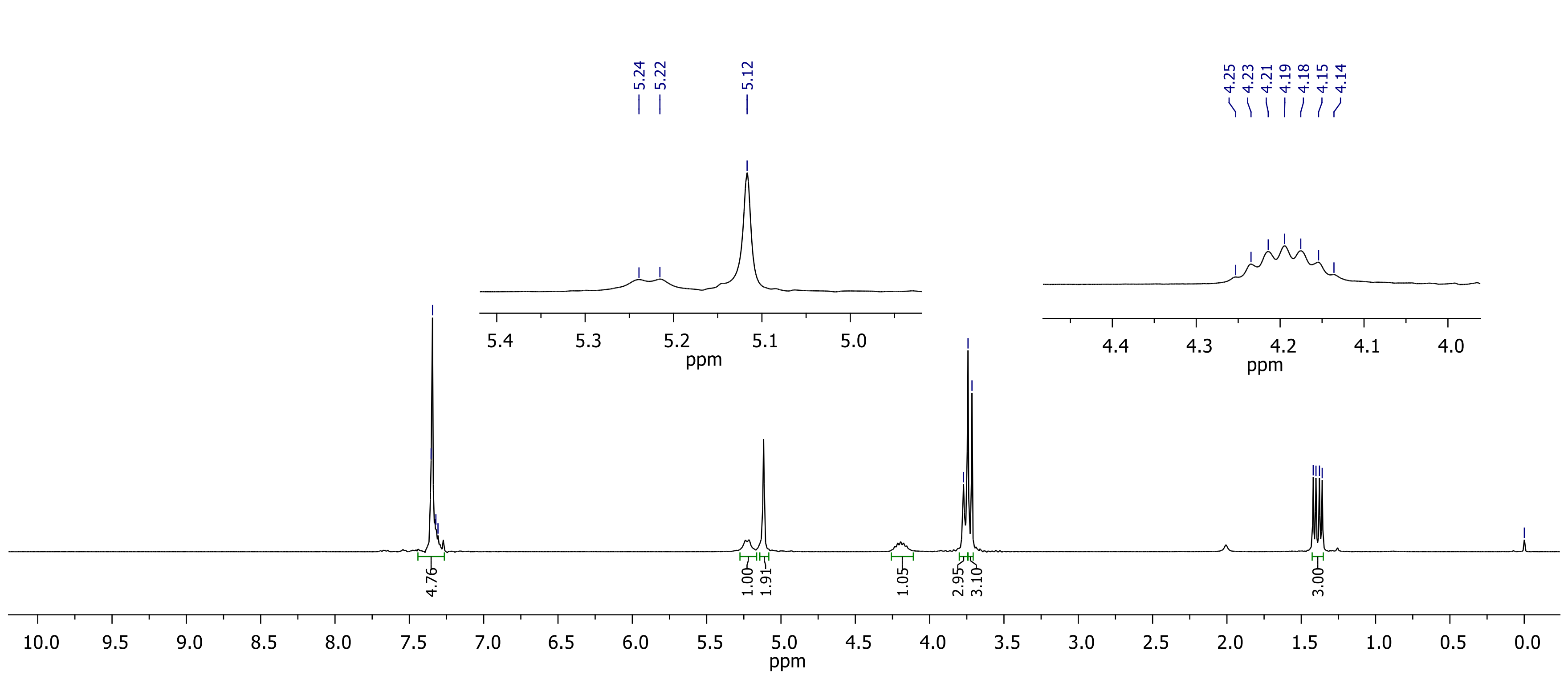

^{13}C NMR [100 MHz/$CDCl_3$]: (*R*)-Benzyl *N*-[1-(dimethoxyphosphoryl)ethyl]carbamate (**3a**)

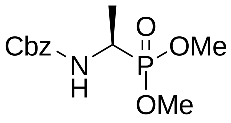

3a

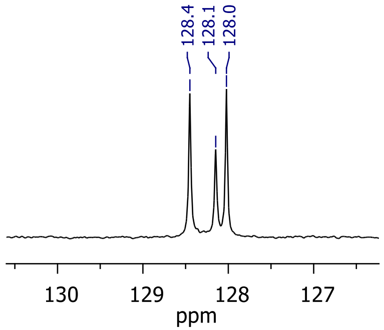

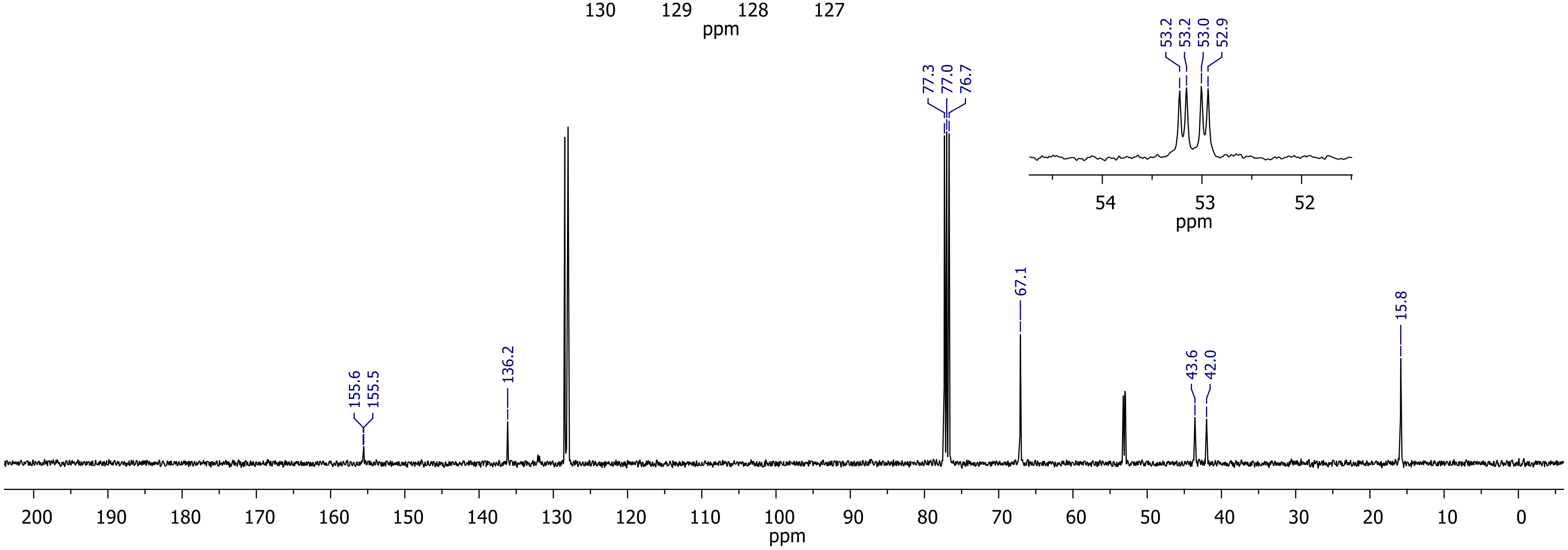

^{31}P NMR [162 MHz/$CDCl_3$]: (*R*)-Benzyl *N*-[1-(dimethoxyphosphoryl)ethyl]carbamate (**3a**)

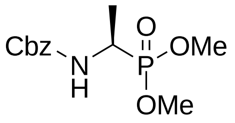

3a

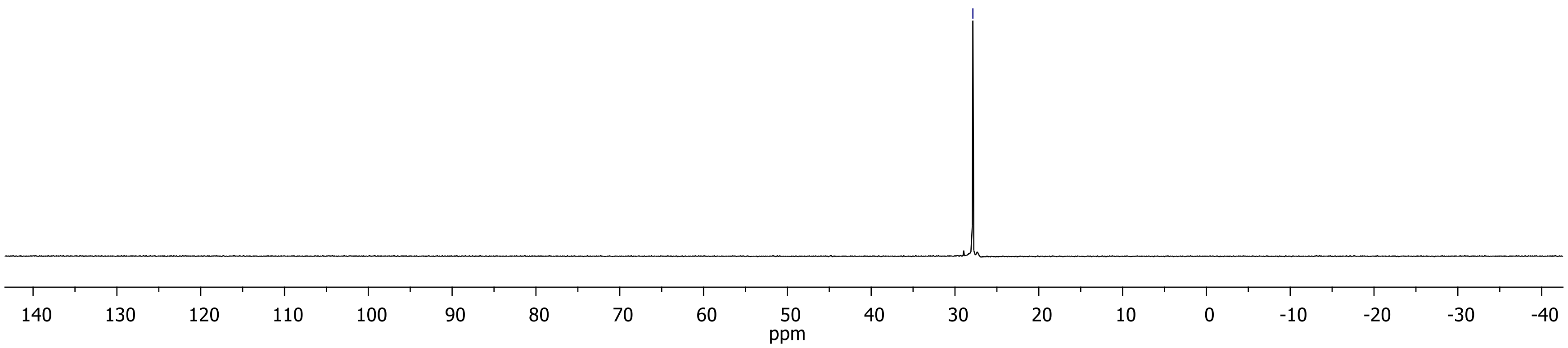

^{1}H NMR [400 MHz/$CDCl_3$/TMS]: (*R*)-Benzyl *N*-[1-(dimethoxyphosphoryl)-2-phenylethyl]carbamate (**3b**)

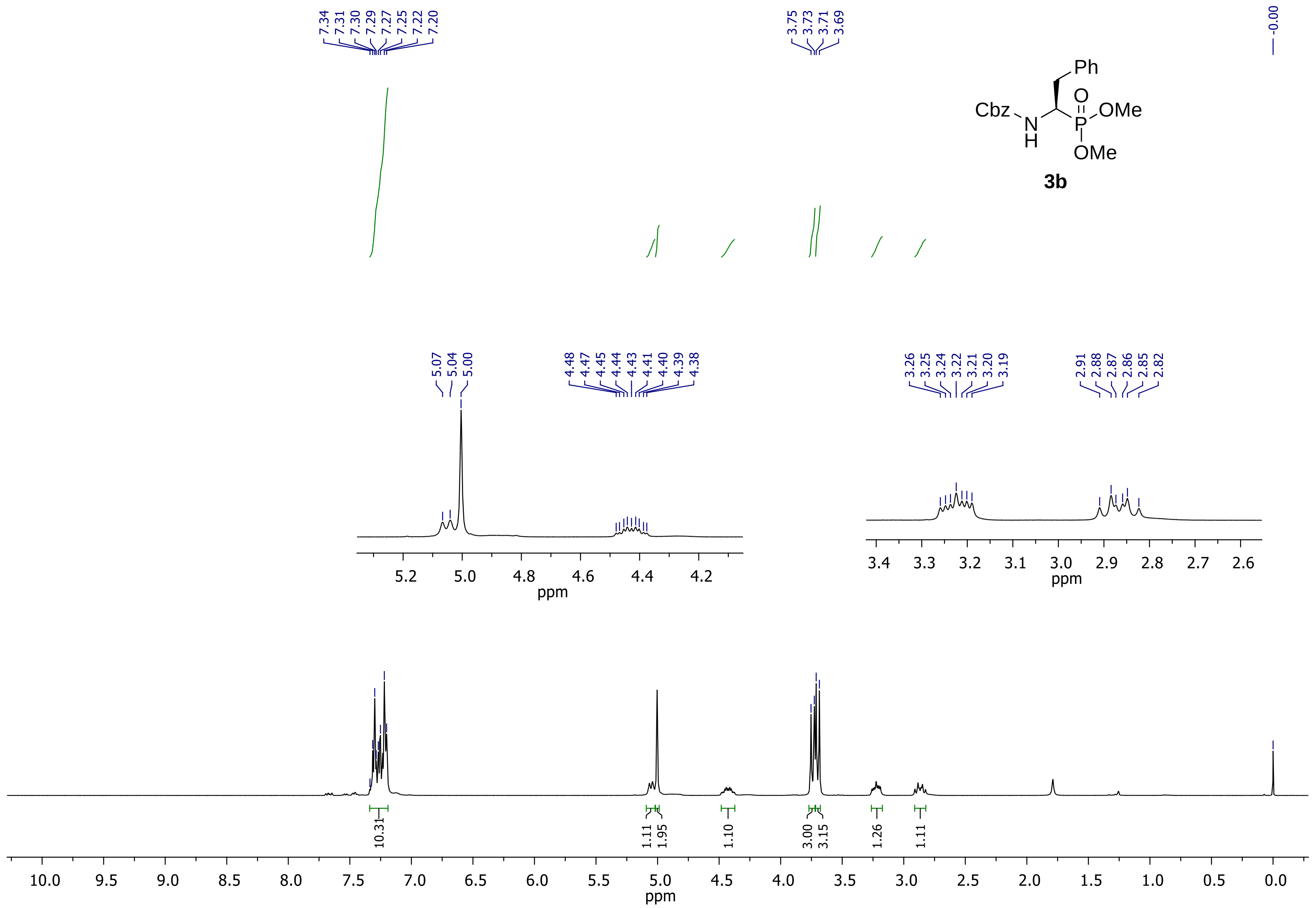

^{13}C NMR [100 MHz/$CDCl_3$]: (*R*)-Benzyl *N*-[1-(dimethoxyphosphoryl)-2-phenylethyl]carbamate (**3b**)

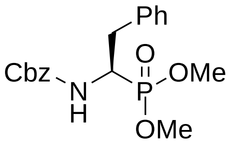

3b

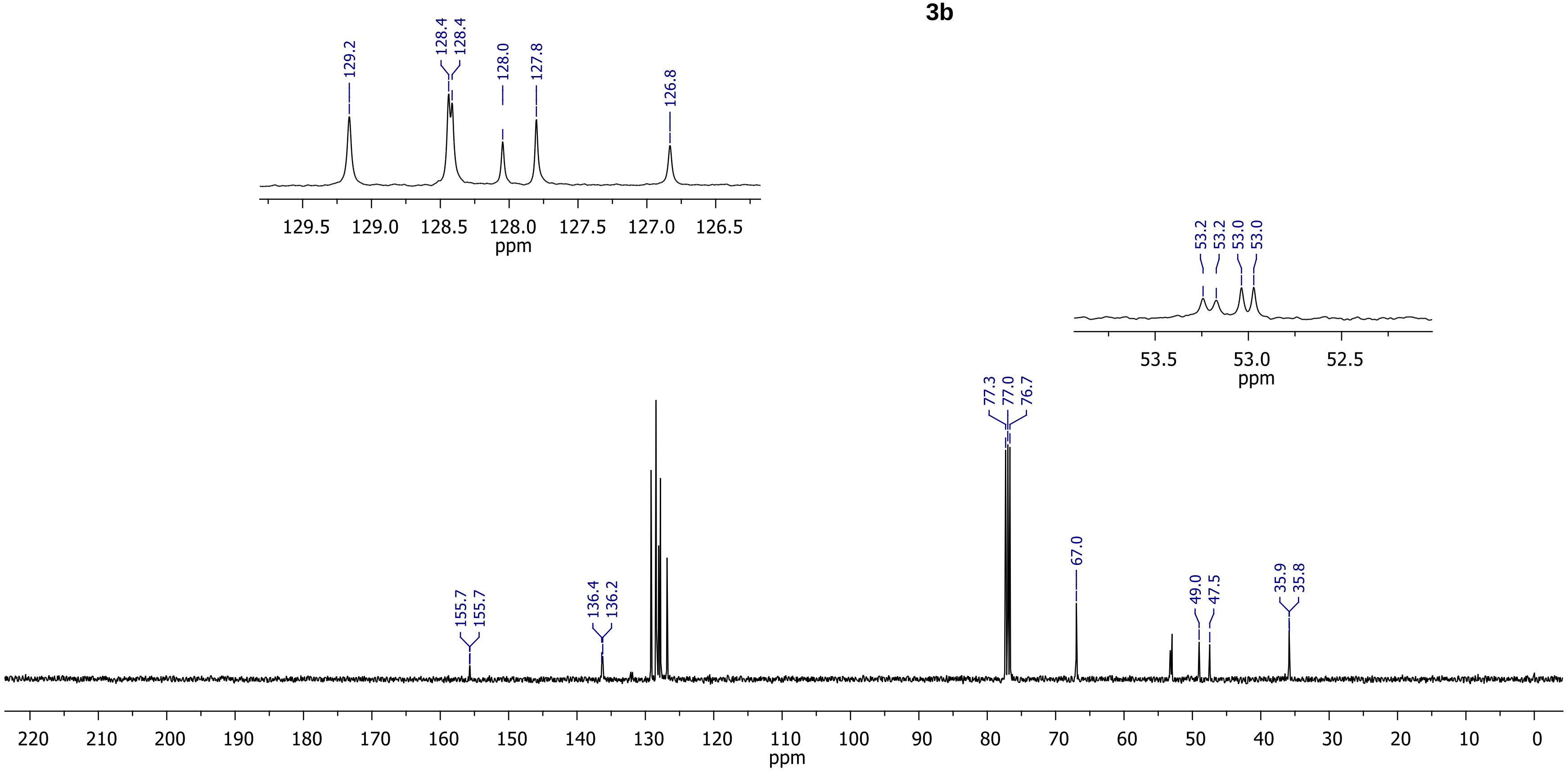

^{31}P NMR [162 MHz/CDCl$_3$]: (*R*)-Benzyl *N*-[1-(dimethoxyphosphoryl)-2-phenylethyl]carbamate (**3b**)

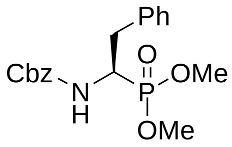

3b

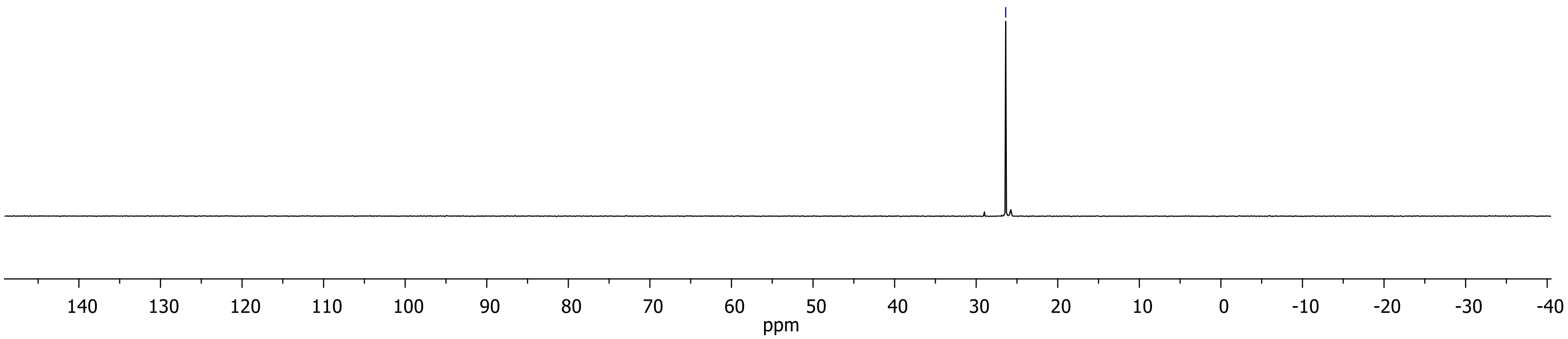

^{1}H NMR [400 MHz/CDCl$_3$/TMS]: (*R*)-*tert*-Butyl *N*-[1-(dimethoxyphosphoryl)-2-phenylethyl]carbamate (**3c**)

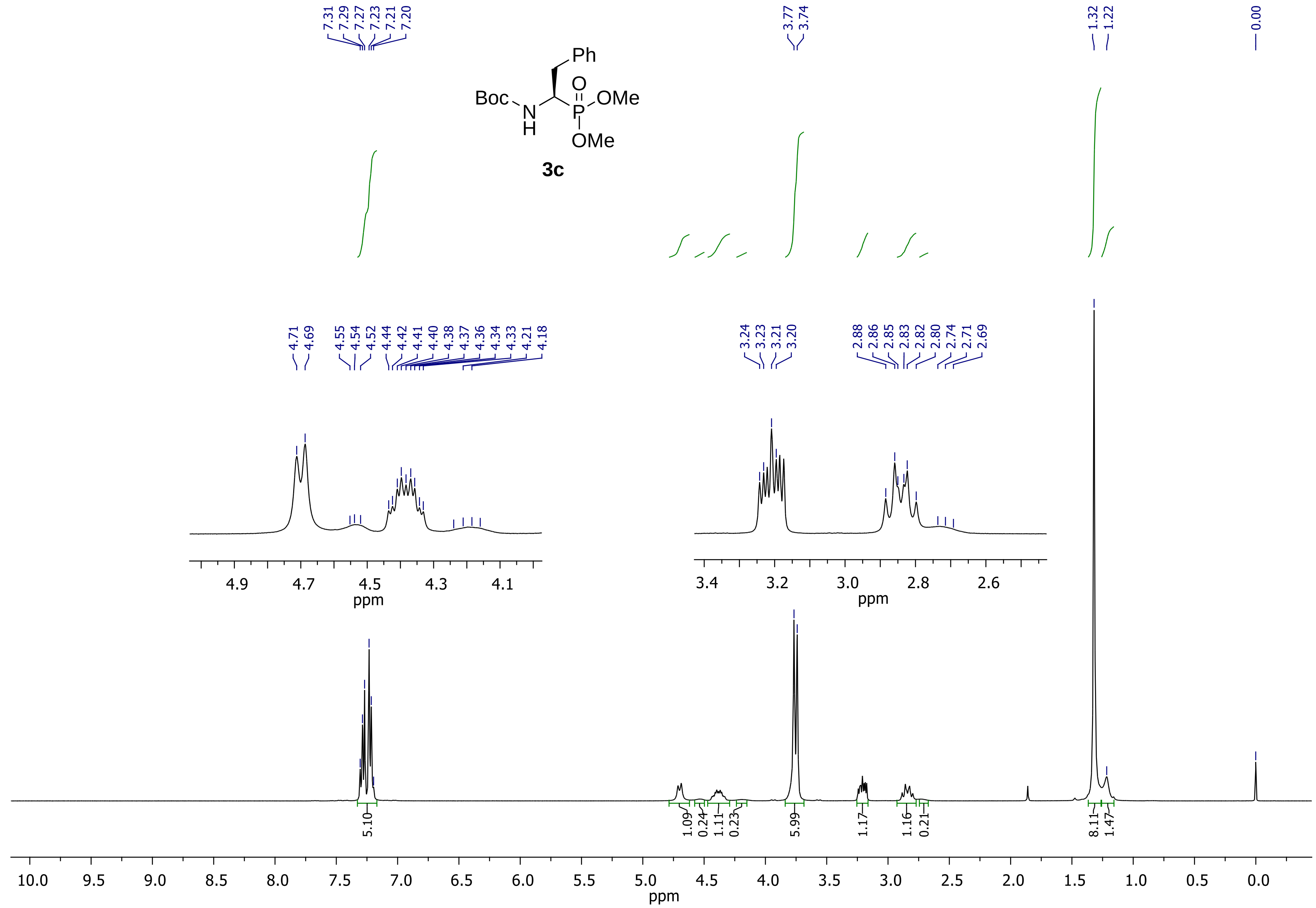

^{13}C NMR [100 MHz/CDCl$_3$]: (*R*)-*tert*-Butyl *N*-[1-(dimethoxyphosphoryl)-2-phenylethyl]carbamate (**3c**)

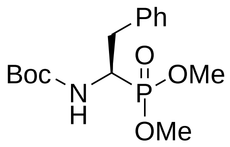

3c

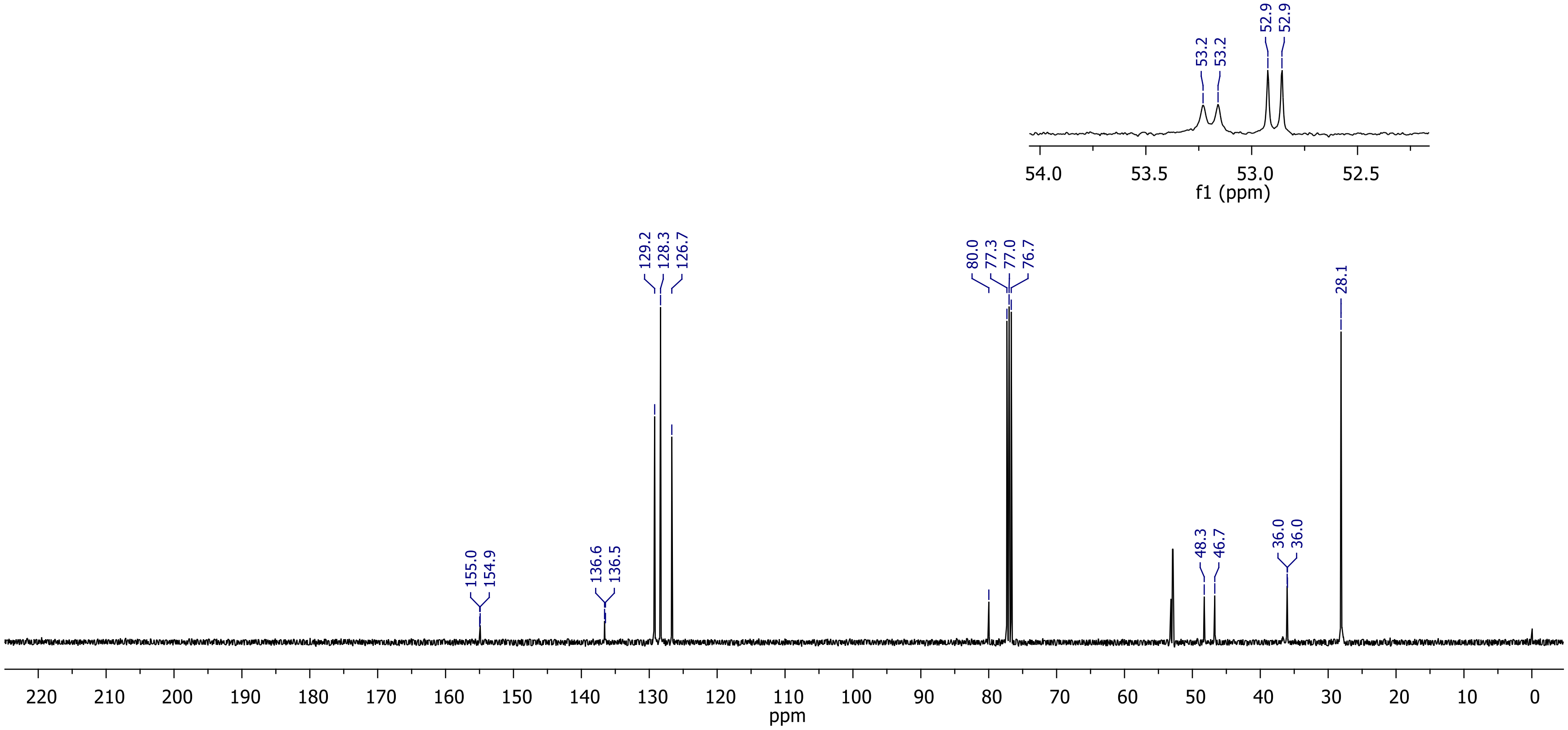

^{31}P NMR [162 MHz/$CDCl_3$]: (*R*)-*tert*-Butyl *N*-[1-(dimethoxyphosphoryl)-2-phenylethyl]carbamate (**3c**)

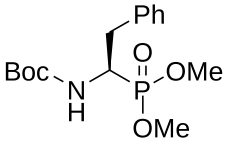

3c

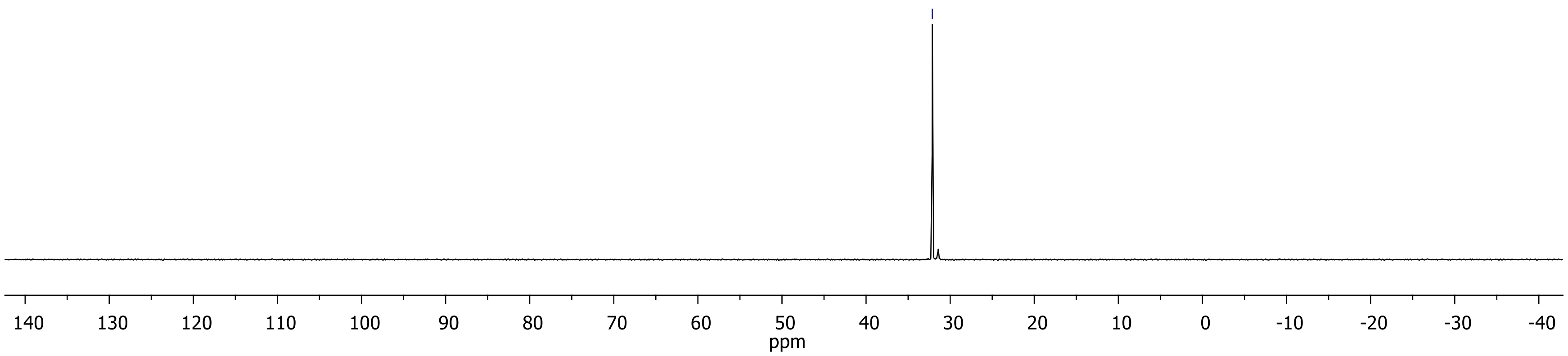

^{1}H NMR [400 MHz/$CDCl_3$/TMS]: (*R*)-Benzyl *N*-[1-(dimethoxyphosphoryl)-2-methylpropyl]carbamate (**3d**)

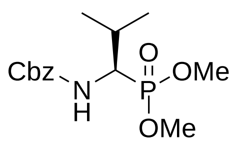

3d

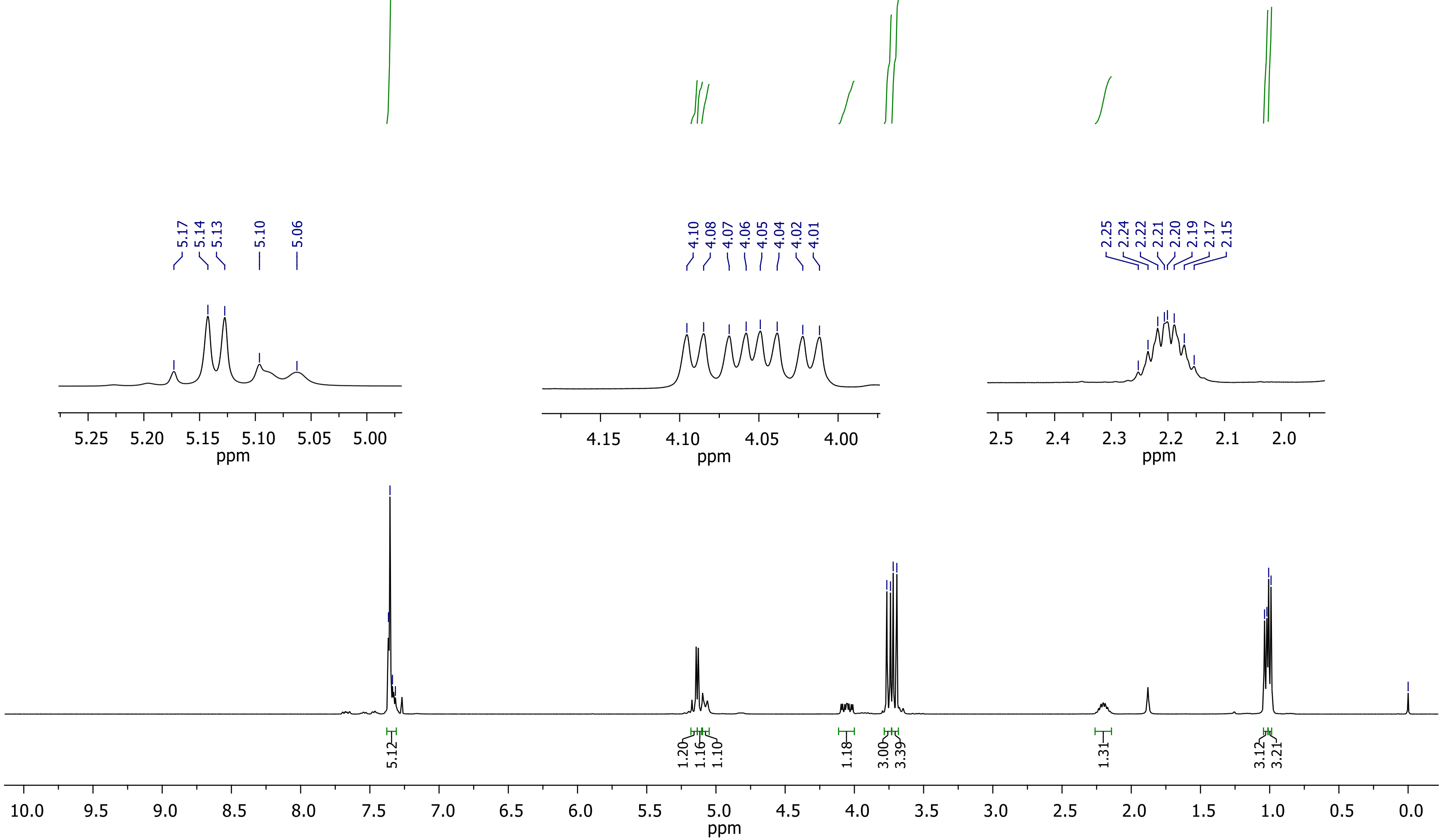

^{13}C NMR [100 MHz/CDCl$_3$] (*R*)-Benzyl *N*-[1-(dimethoxyphosphoryl)-2-methylpropyl]carbamate (**3d**)

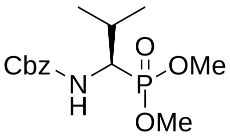

3d

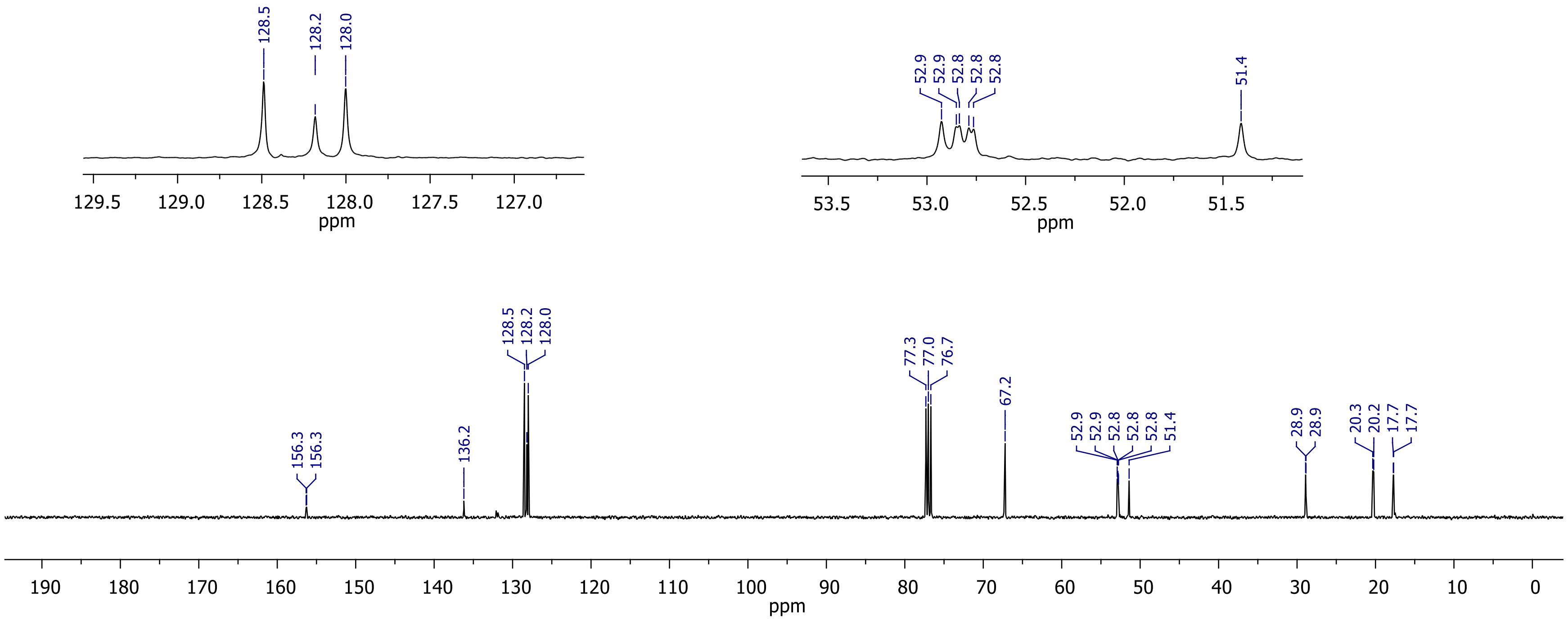

^{31}P NMR [162 MHz/$CDCl_3$]: (*R*)-Benzyl *N*-[1-(dimethoxyphosphoryl)-2-methylpropyl]carbamate (**3d**)

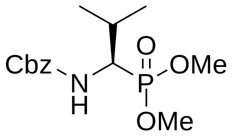

3d

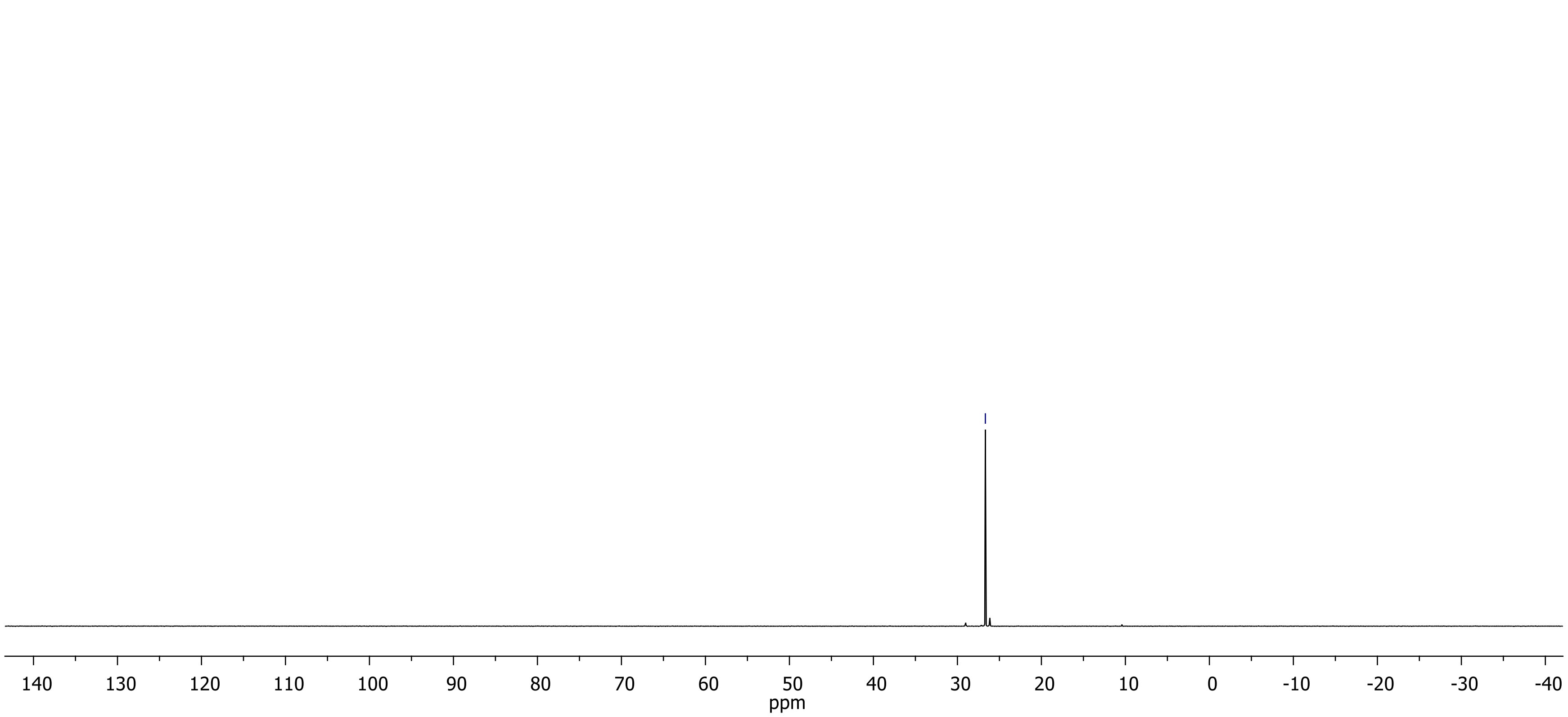

^{1}H NMR [400 MHz/CDCl$_3$/TMS]: (*R*)-*tert*-Butyl *N*-[1-(dimethoxyphosphoryl)-2-methylpropyl]carbamate (**3e**)

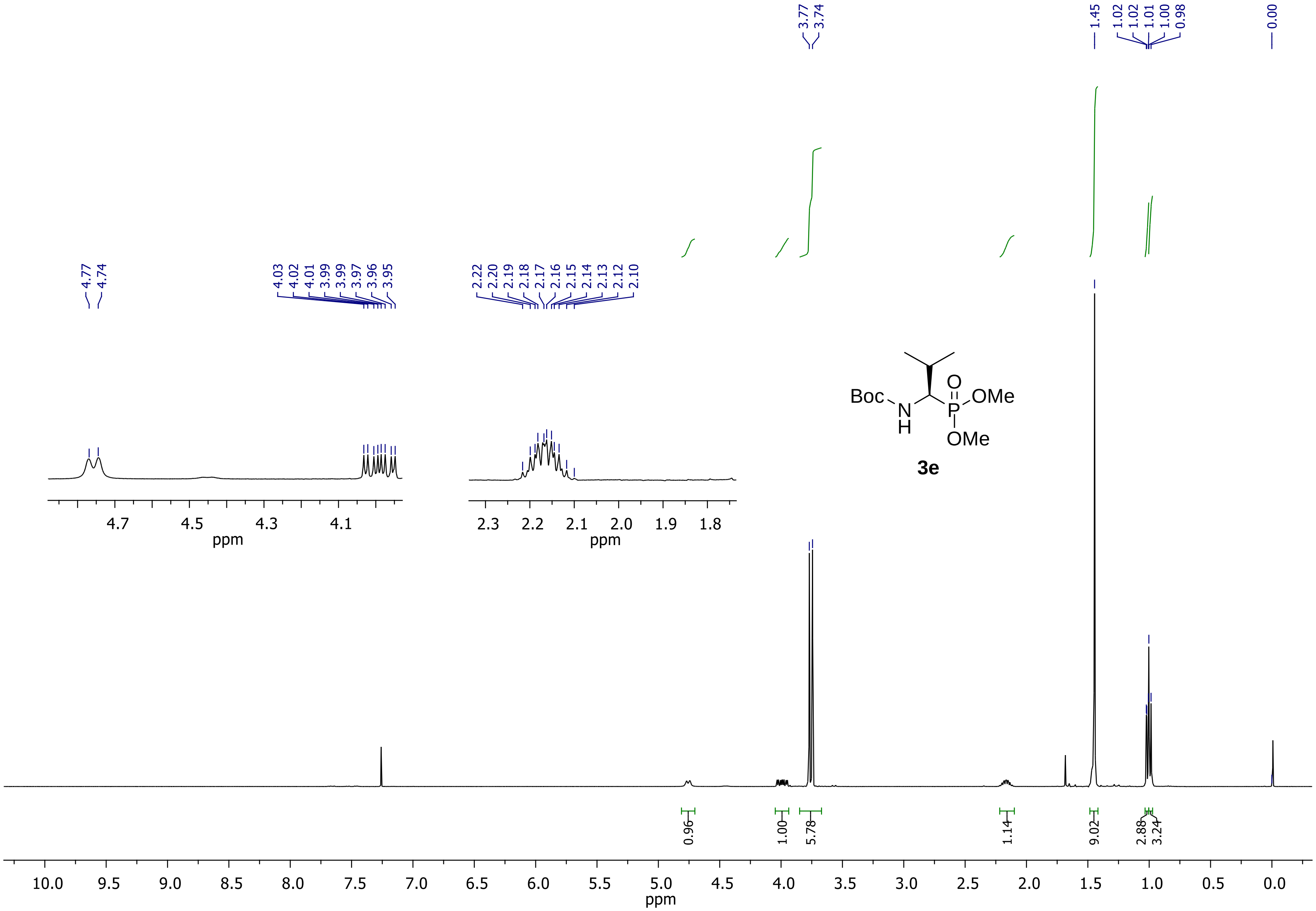

^{13}C NMR [100 MHz/CDCl$_3$]: (*R*)-*tert*-Butyl *N*-[1-(dimethoxyphosphoryl)-2-methylpropyl]carbamate (**3e**)

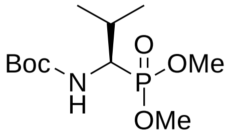

3e

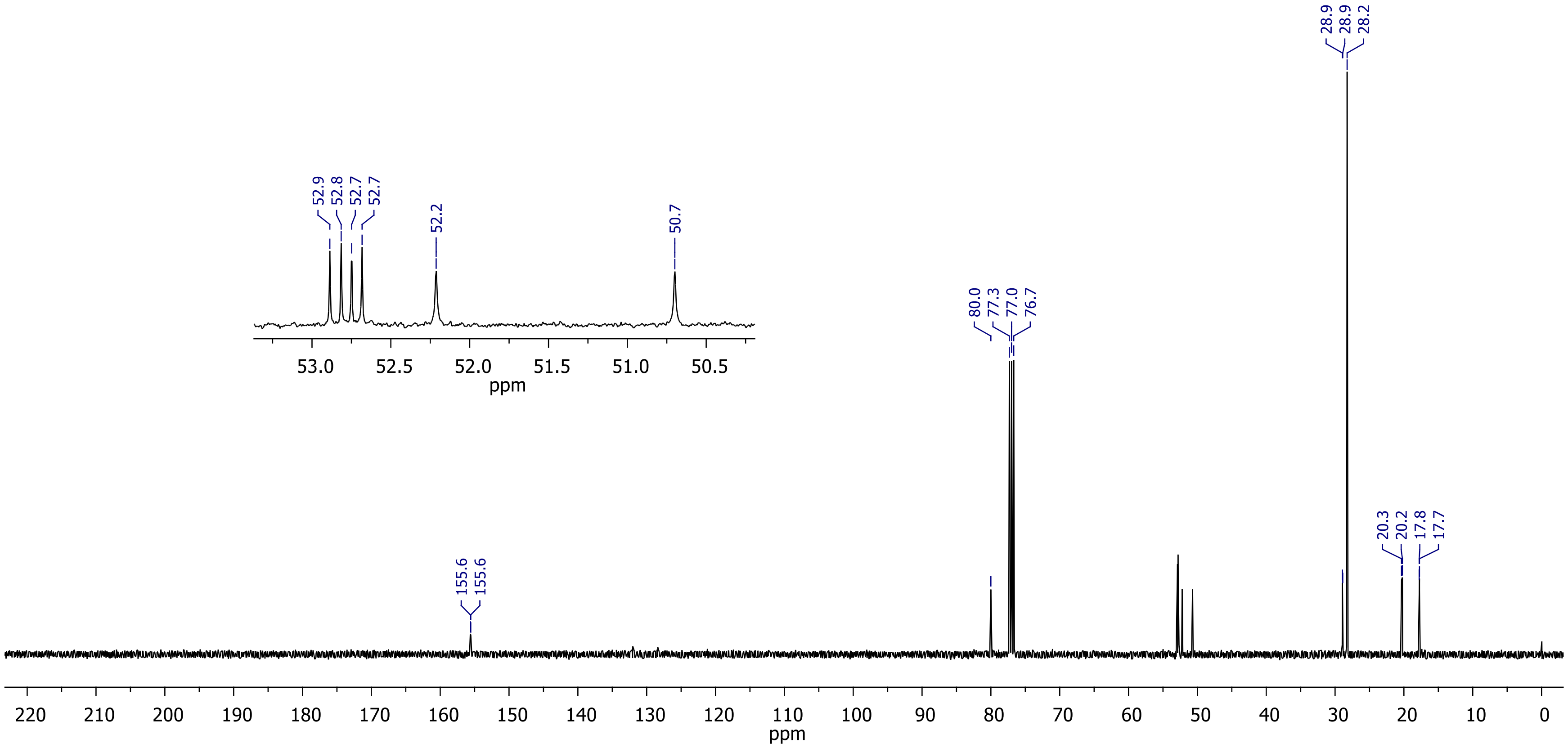

^{31}P NMR [162 MHz/$CDCl_3$]: (*R*)-*tert*-Butyl *N*-[1-(dimethoxyphosphoryl)-2-methylpropyl]carbamate (**3e**)

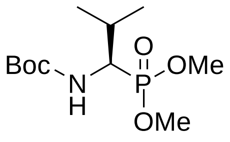

3e

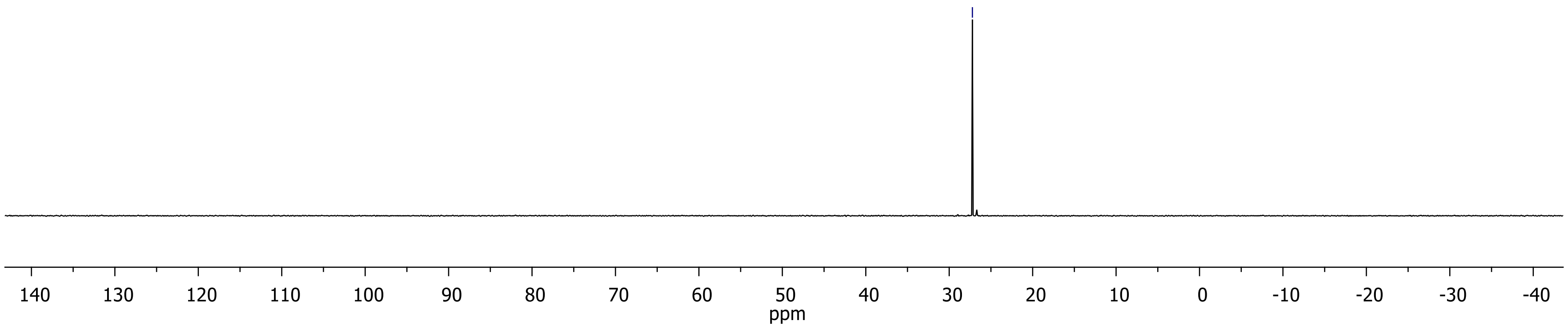

^{1}H NMR [400 MHz/CDCl$_3$/TMS]: (*R*)-Benzyl *N*-[1-(dimethoxyphosphoryl)-3-methylbutyl]carbamate (**3f**)

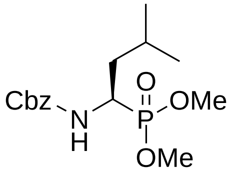

3f

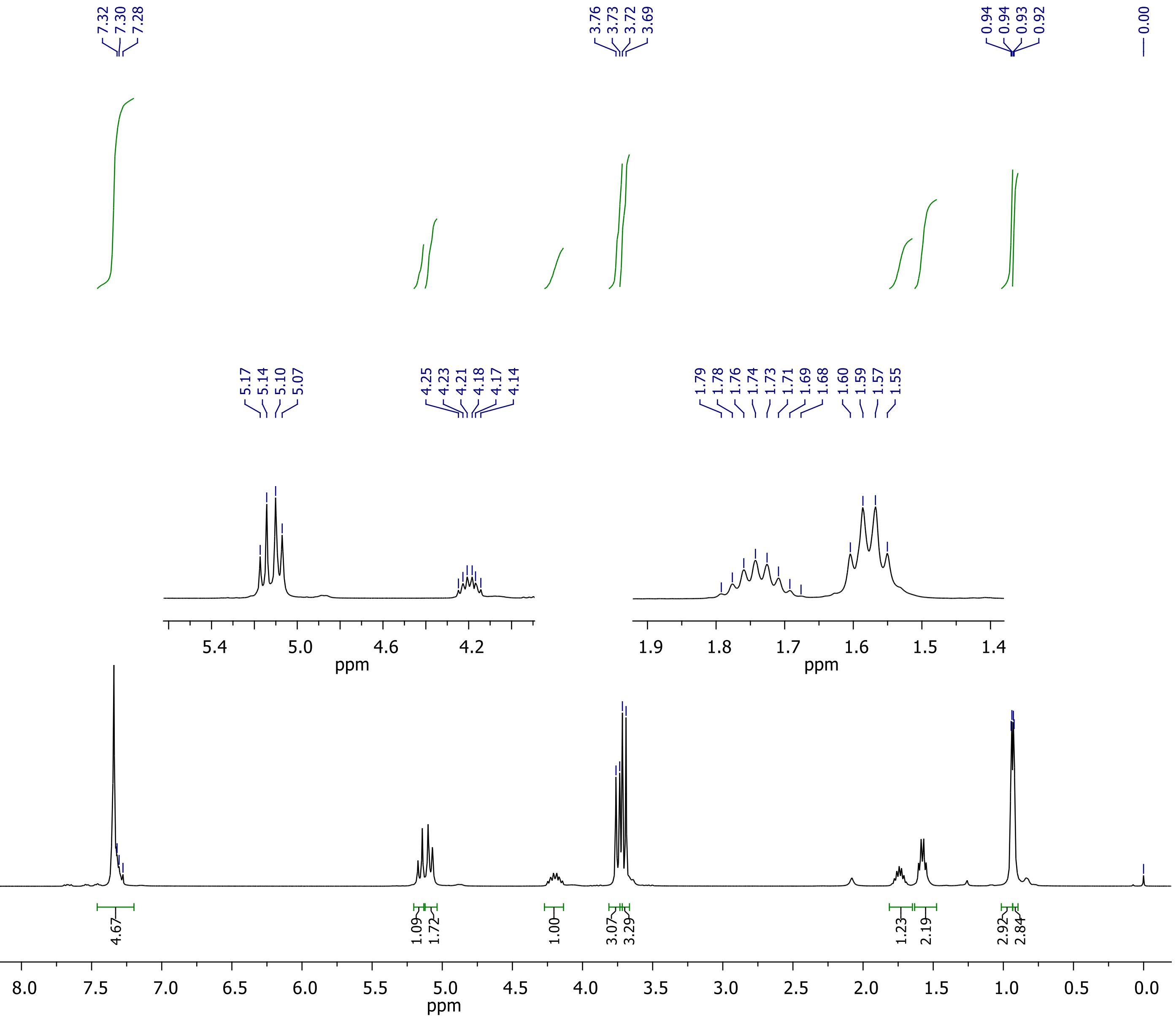

^{13}C NMR [100 MHz/CDCl$_3$]: (*R*)-Benzyl *N*-[1-(dimethoxyphosphoryl)-3-methylbutyl]carbamate (**3f**)

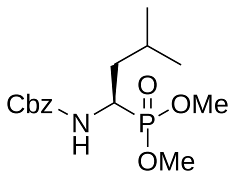

3f

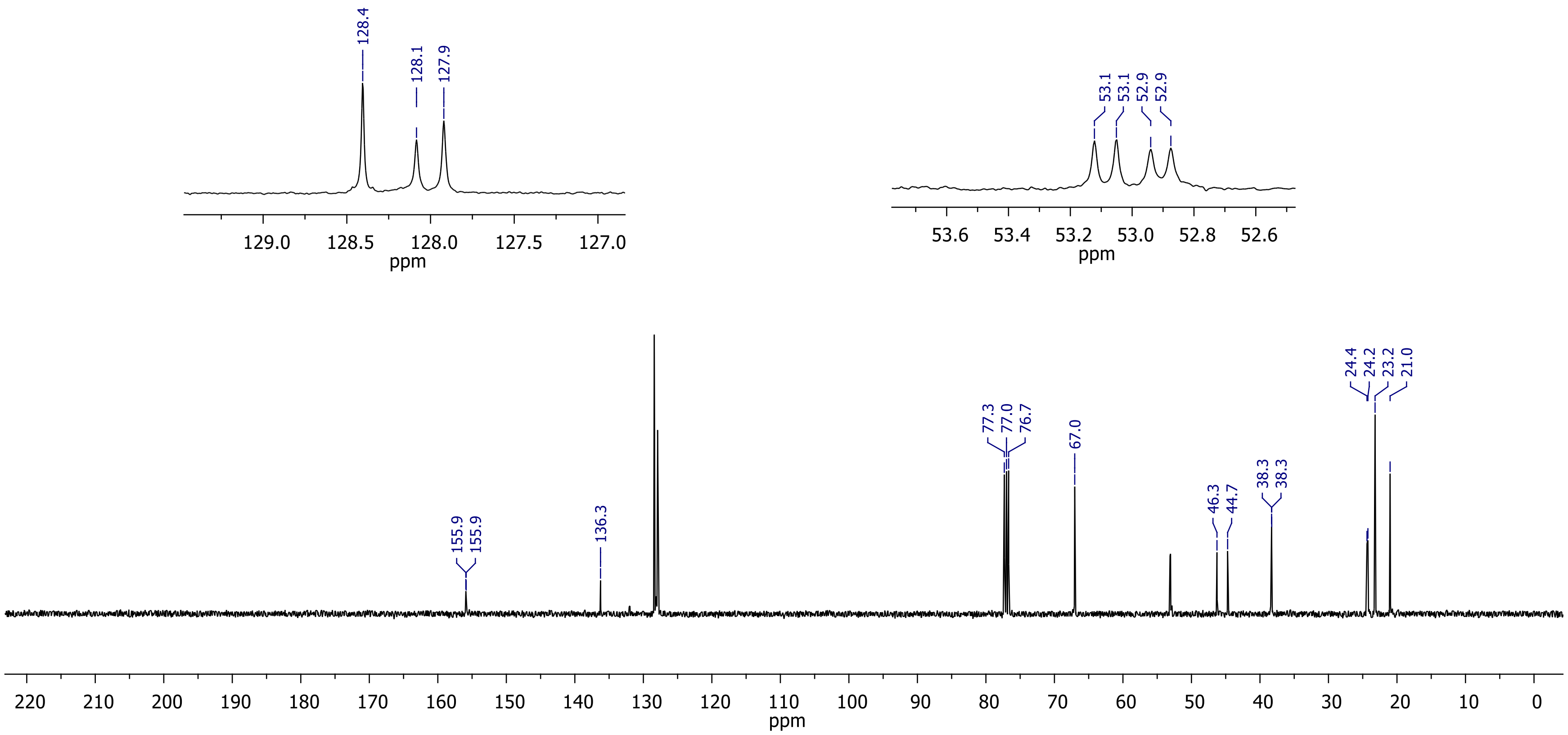

^{31}P NMR [162 MHz/$CDCl_3$]: (*R*)-Benzyl *N*-[1-(dimethoxyphosphoryl)-3-methylbutyl]carbamate (**3f**)

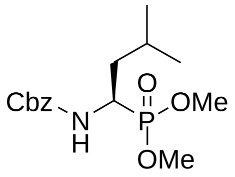

3f

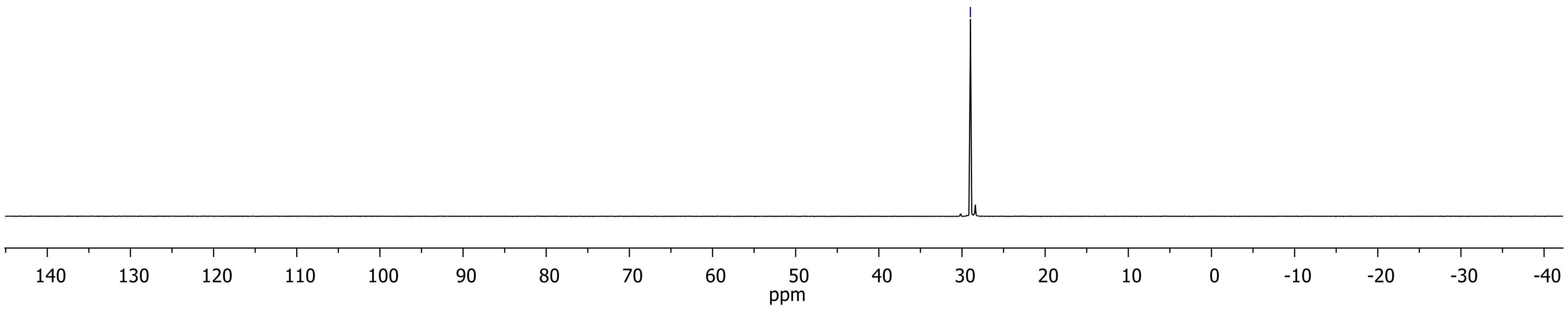

^{1}H NMR [400 MHz/$CDCl_3$/TMS]: (*R*)-Benzyl *N*-[1-(dimethoxyphosphoryl)butyl]carbamate (**3g**)

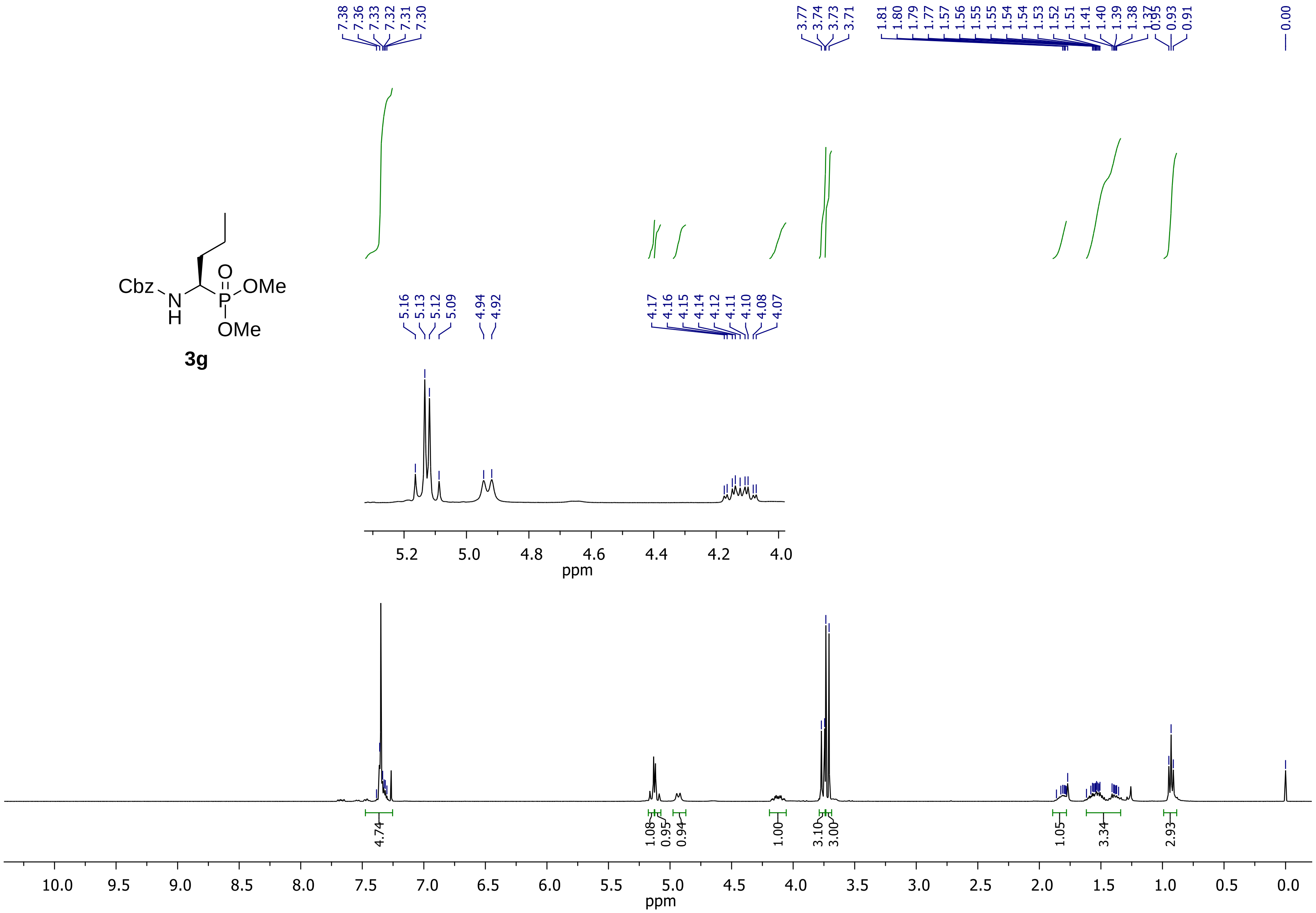

^{13}C NMR [100 MHz/CDCl$_3$]: (*R*)-Benzyl *N*-[1-(dimethoxyphosphoryl)butyl]carbamate (**3g**)

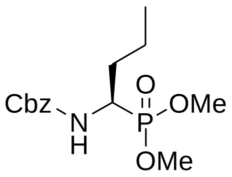

3g

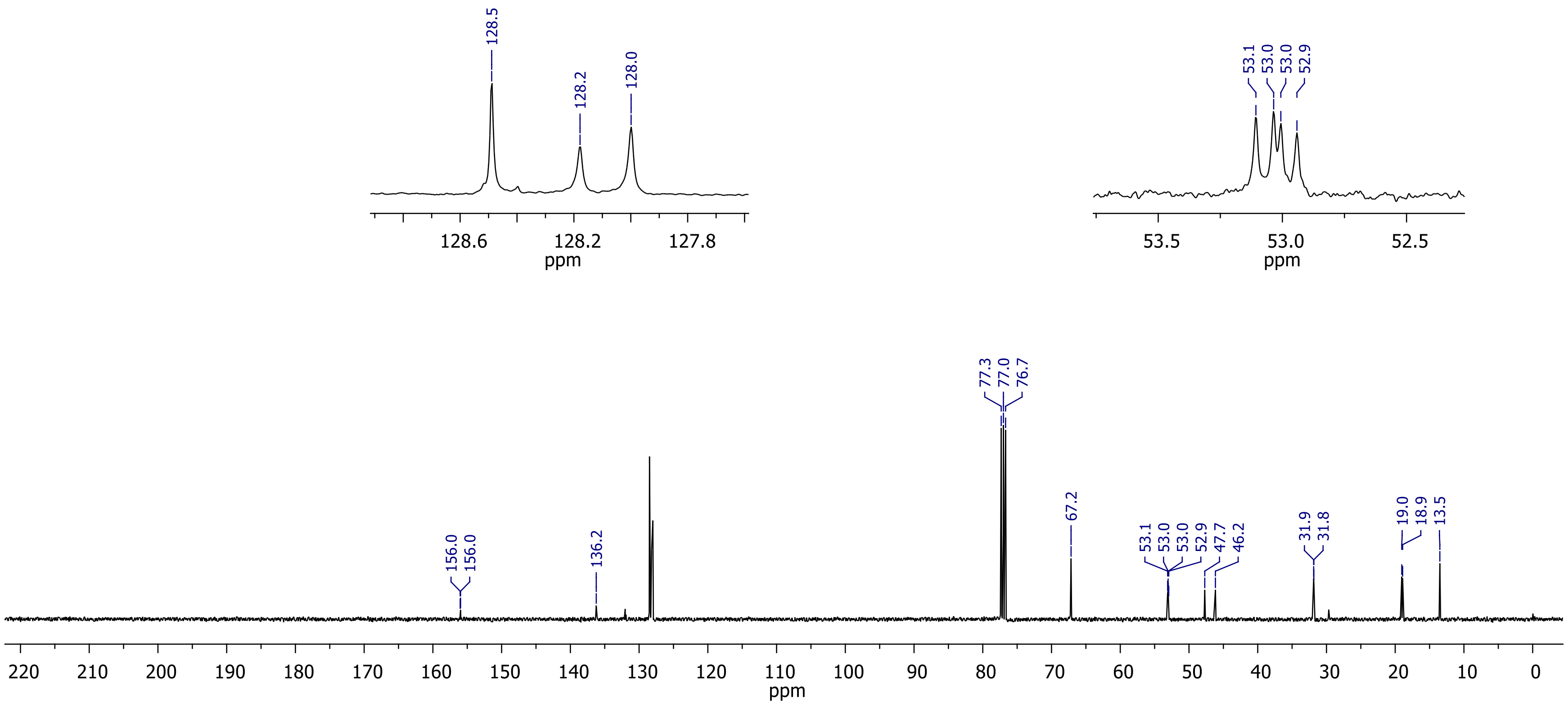

^{31}P NMR [162 MHz/$CDCl_3$]: (*R*)-Benzyl *N*-[1-(dimethoxyphosphoryl)butyl]carbamate (**3g**)

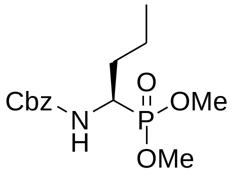

3g

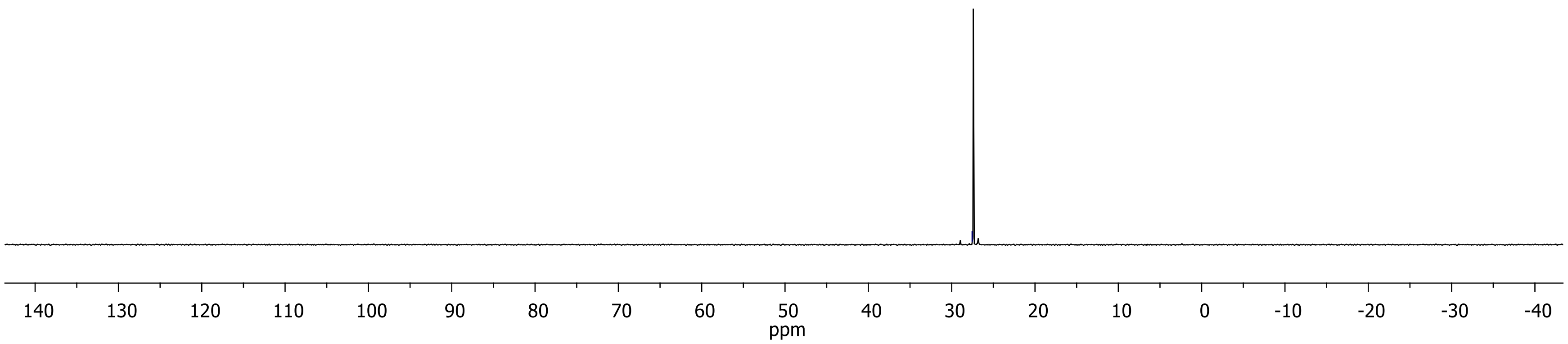

HRMS: (*R*)-Benzyl *N*-[1-(dimethoxyphosphoryl)butyl]carbamate (**3g**)

Tolerance = 10.0 PPM / DBE: min = -20.0, max = 100.0
Element prediction: Off
Number of isotope peaks used for i-FIT = 2
Monoisotopic Mass, Even Electron Ions
56 formula(e) evaluated with 1 results within limits (up to 5 closest results for each mass)
Elements Used:

Mass	RA	Calc. Mass	mDa	PPM	DBE	Formula	i-FIT	i-FIT Norm	Fit Conf %	C	H	N	O	Na	P
338.1135	100.00	338.1133	0.2	0.6	4.5	C14 H22 N O5 Na P	311.9	n/a	n/a	14	22	1	5	1	1

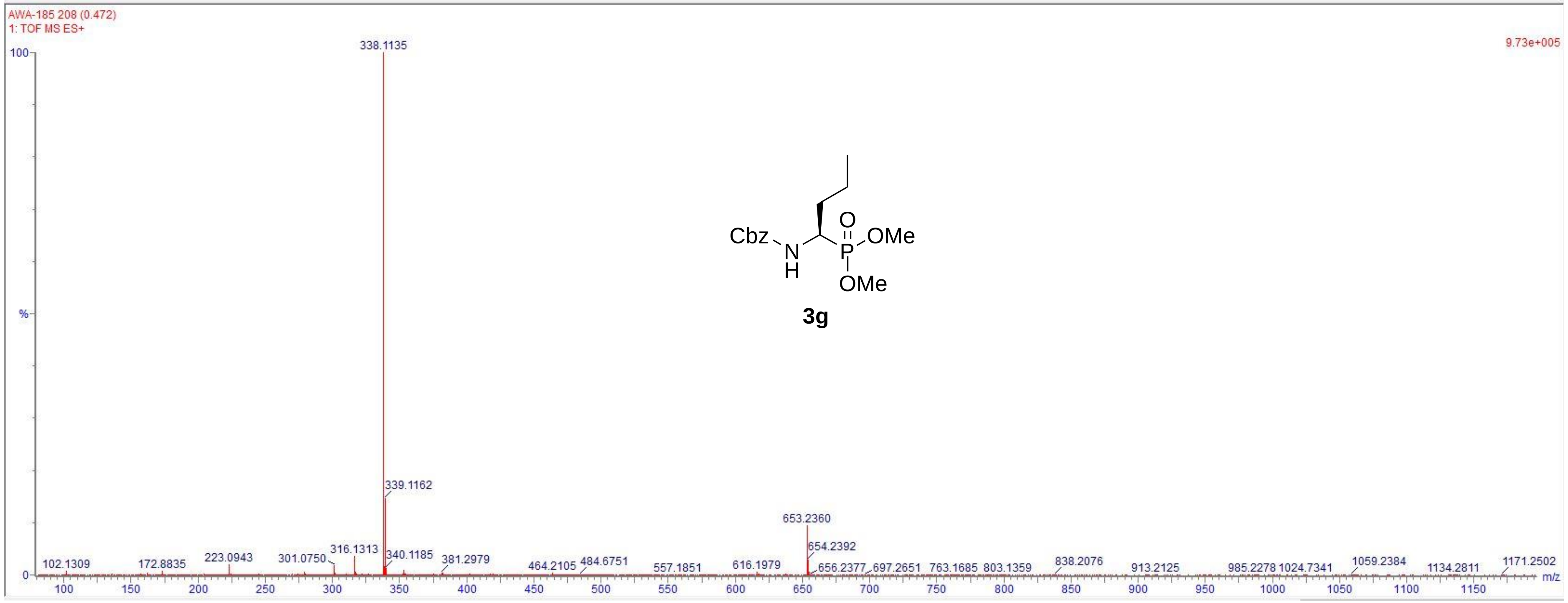

^{1}H NMR [400 MHz/$CDCl_3$/TMS]: (*R*)-Benzyl *N*-[1-(dimethoxyphosphoryl)pentyl]carbamate (**3h**)

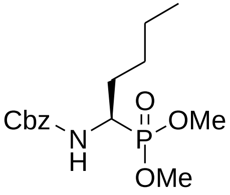

3h

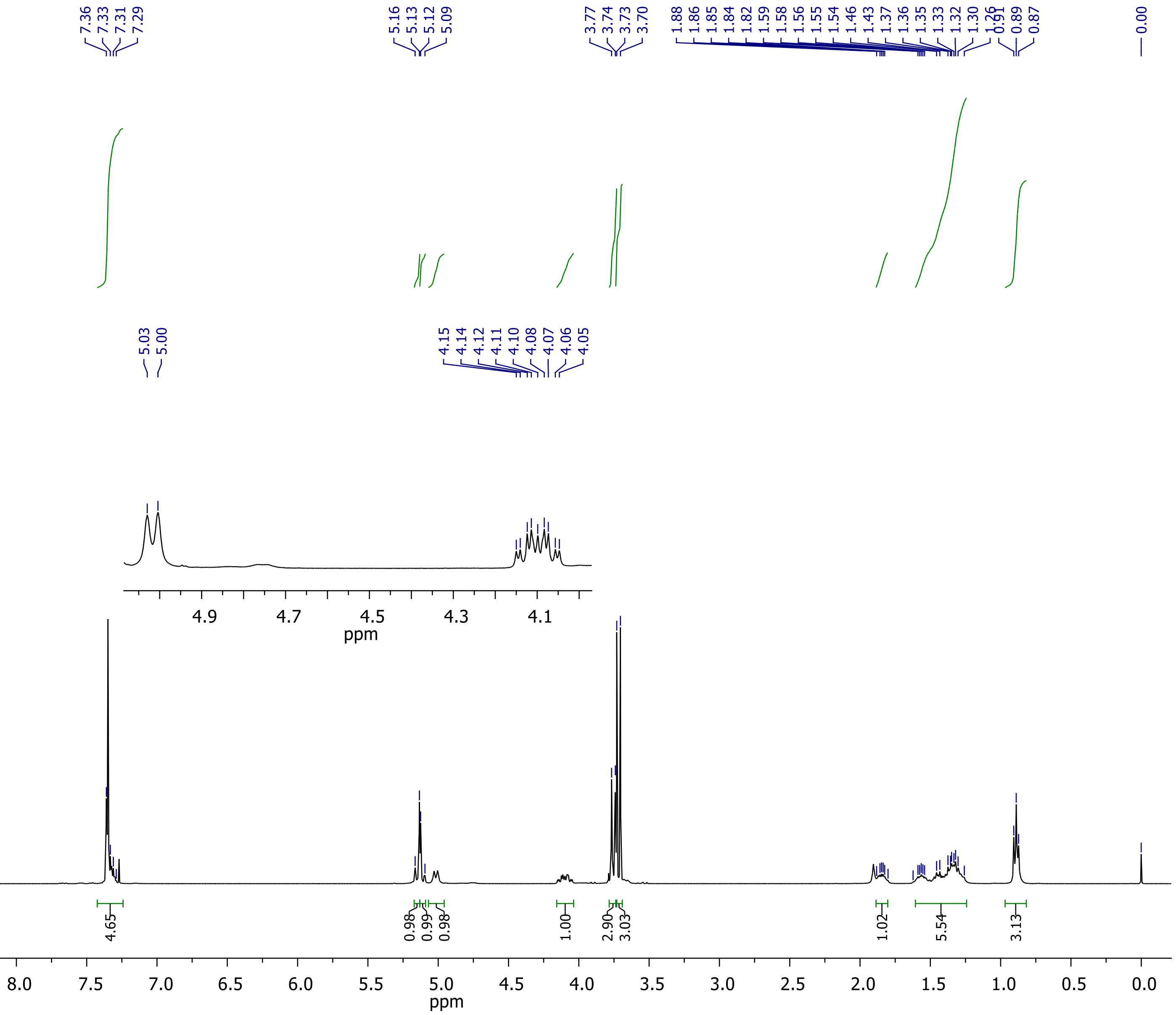

^{13}C NMR [100 MHz/$CDCl_3$]: (*R*)-Benzyl *N*-[1-(dimethoxyphosphoryl)pentyl]carbamate (**3h**)

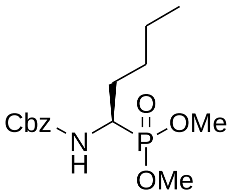

3h

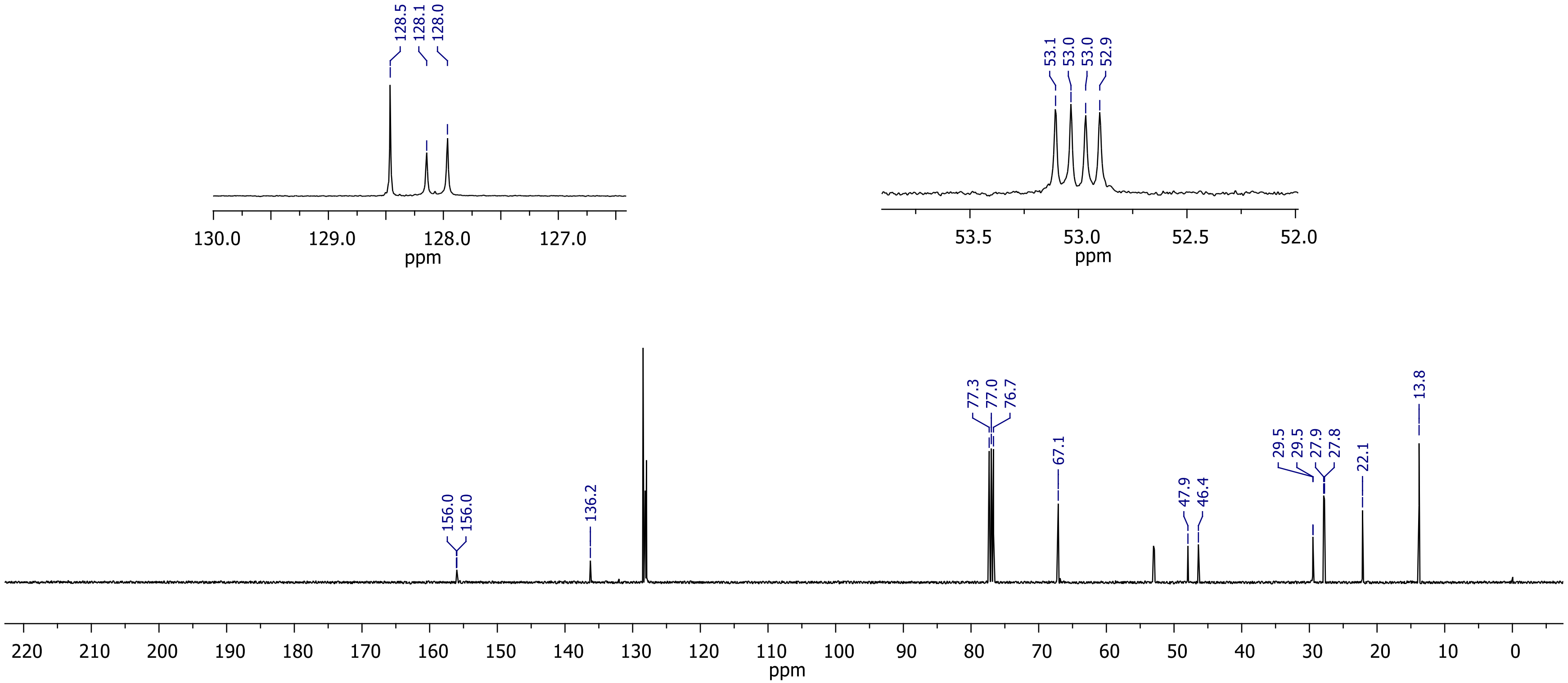

^{31}P NMR [162 MHz/$CDCl_3$]: (*R*)-Benzyl *N*-[1-(dimethoxyphosphoryl)pentyl]carbamate (**3h**)

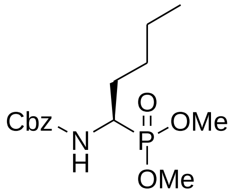

3h

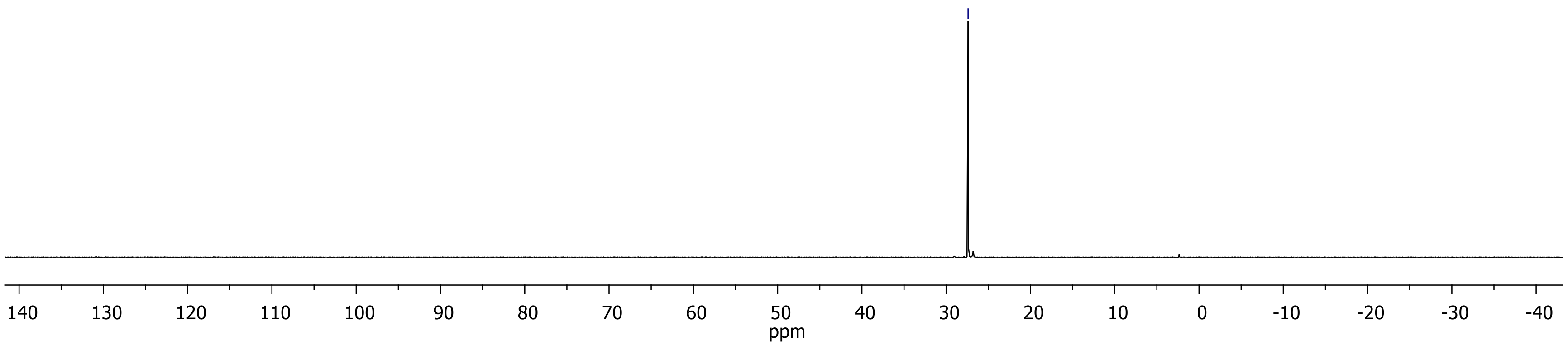

^{1}H NMR [400 MHz/$CDCl_3$/TMS]: (*R*)-Benzyl *N*-[cyclohexyl(dimethoxyphosphoryl)methyl]carbamate (**3i**)

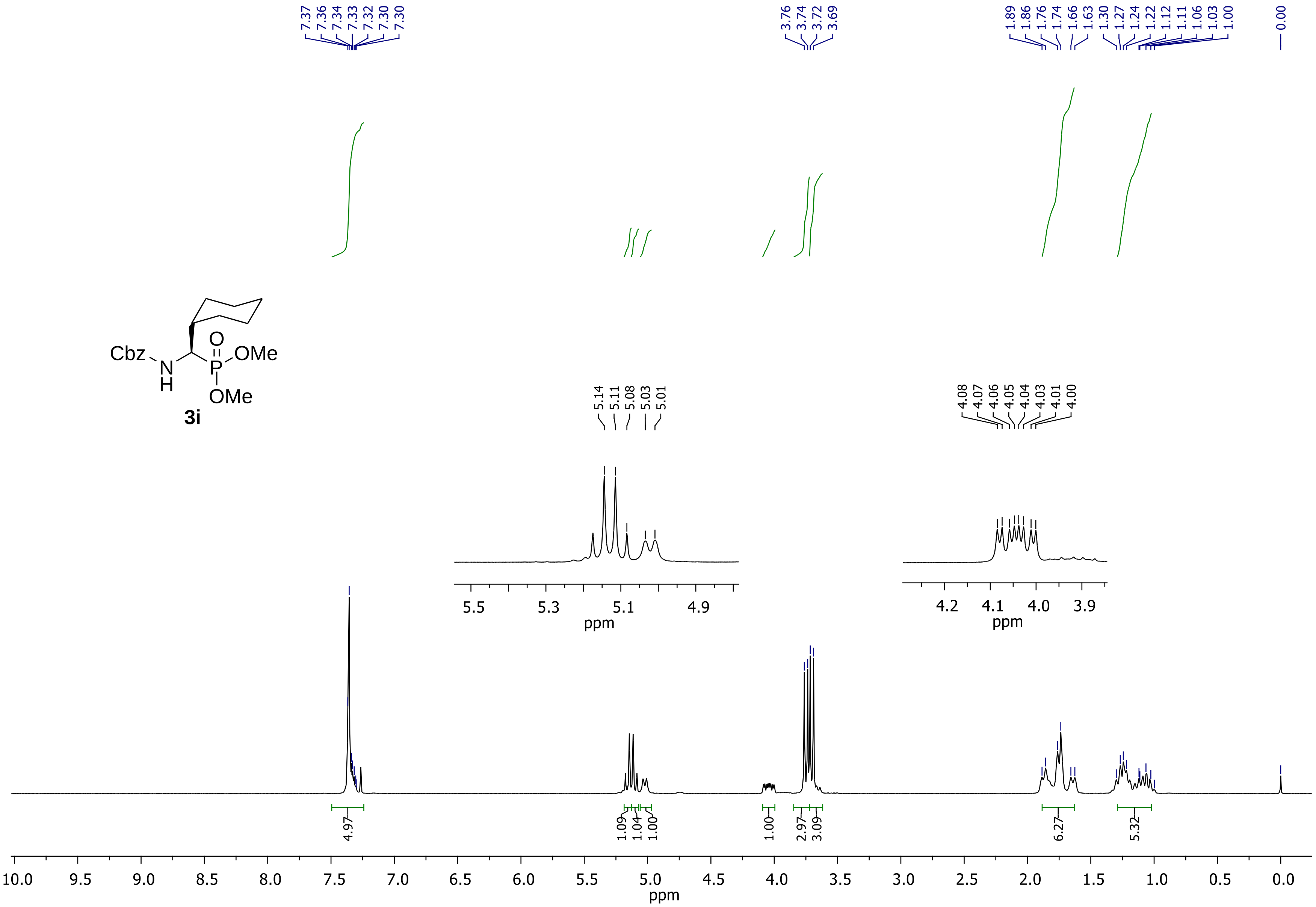

^{13}C NMR [100 MHz/$CDCl_3$]: (*R*)-Benzyl *N*-[cyclohexyl(dimethoxyphosphoryl)methyl]carbamate (**3i**)

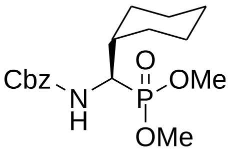

3i

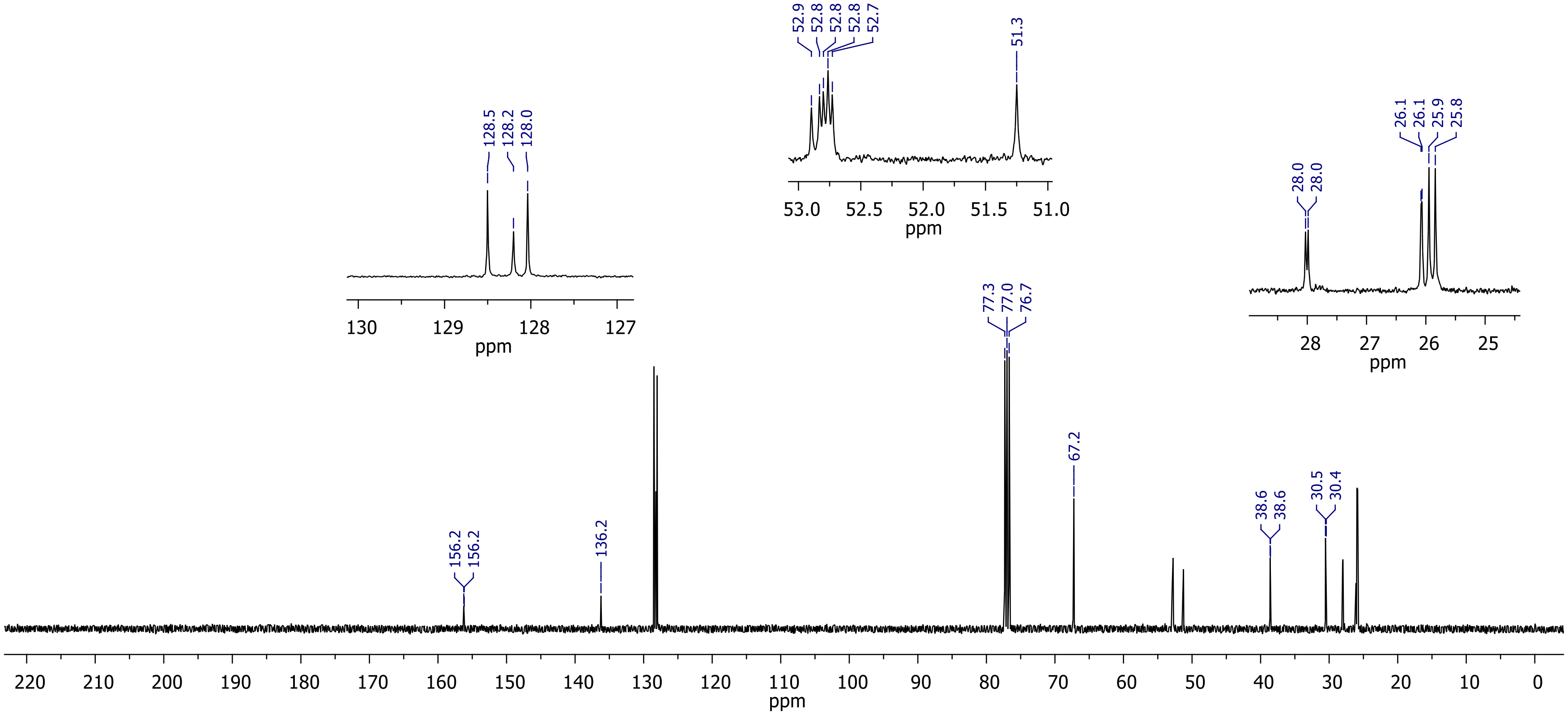

^{31}P NMR [162 MHz/$CDCl_3$]: (*R*)-Benzyl *N*-[cyclohexyl(dimethoxyphosphoryl)methyl]carbamate (**3i**)

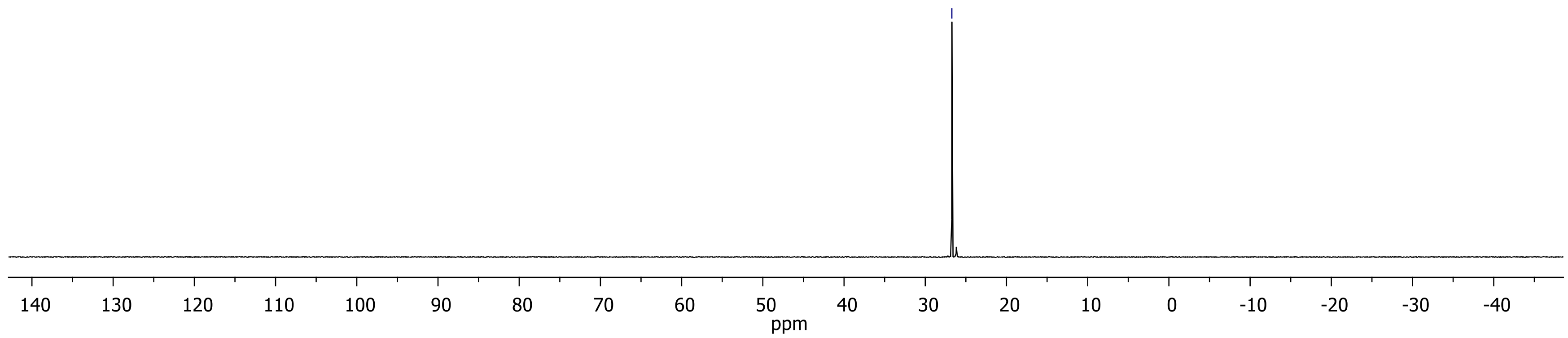

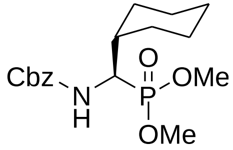

3i

^{1}H NMR [400 MHz/$CDCl_3$/TMS]: (*R*)-Benzyl *N*-[2-(*tert*-butoxy)-1-(dimethoxyphosphoryl)ethyl]carbamate (**3j**)

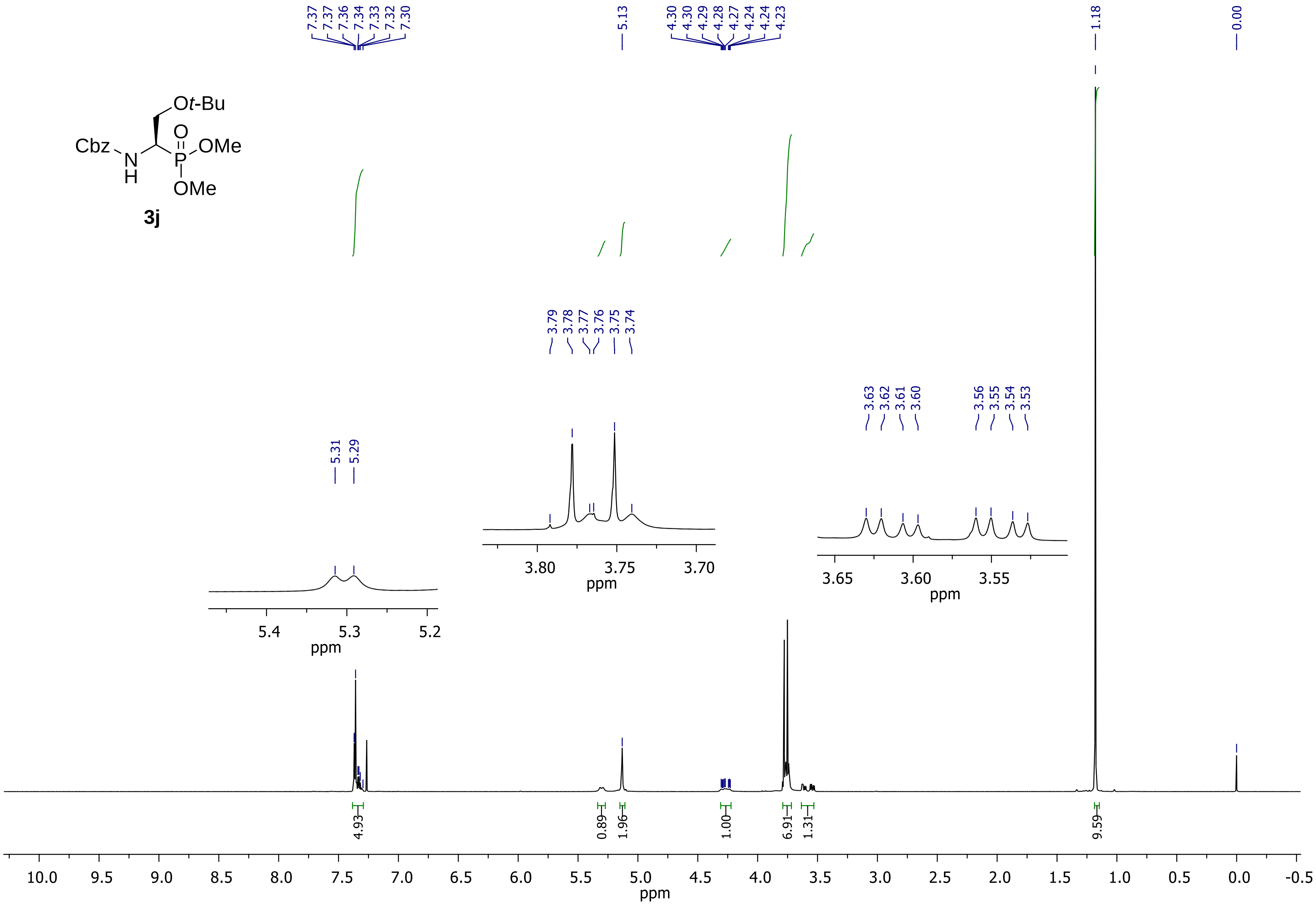

^{13}C NMR [100 MHz/$CDCl_3$]: (*R*)-Benzyl *N*-[2-(*tert*-butoxy)-1-(dimethoxyphosphoryl)ethyl]carbamate (**3j**)

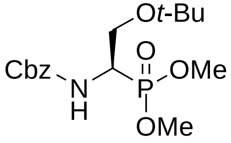

3j

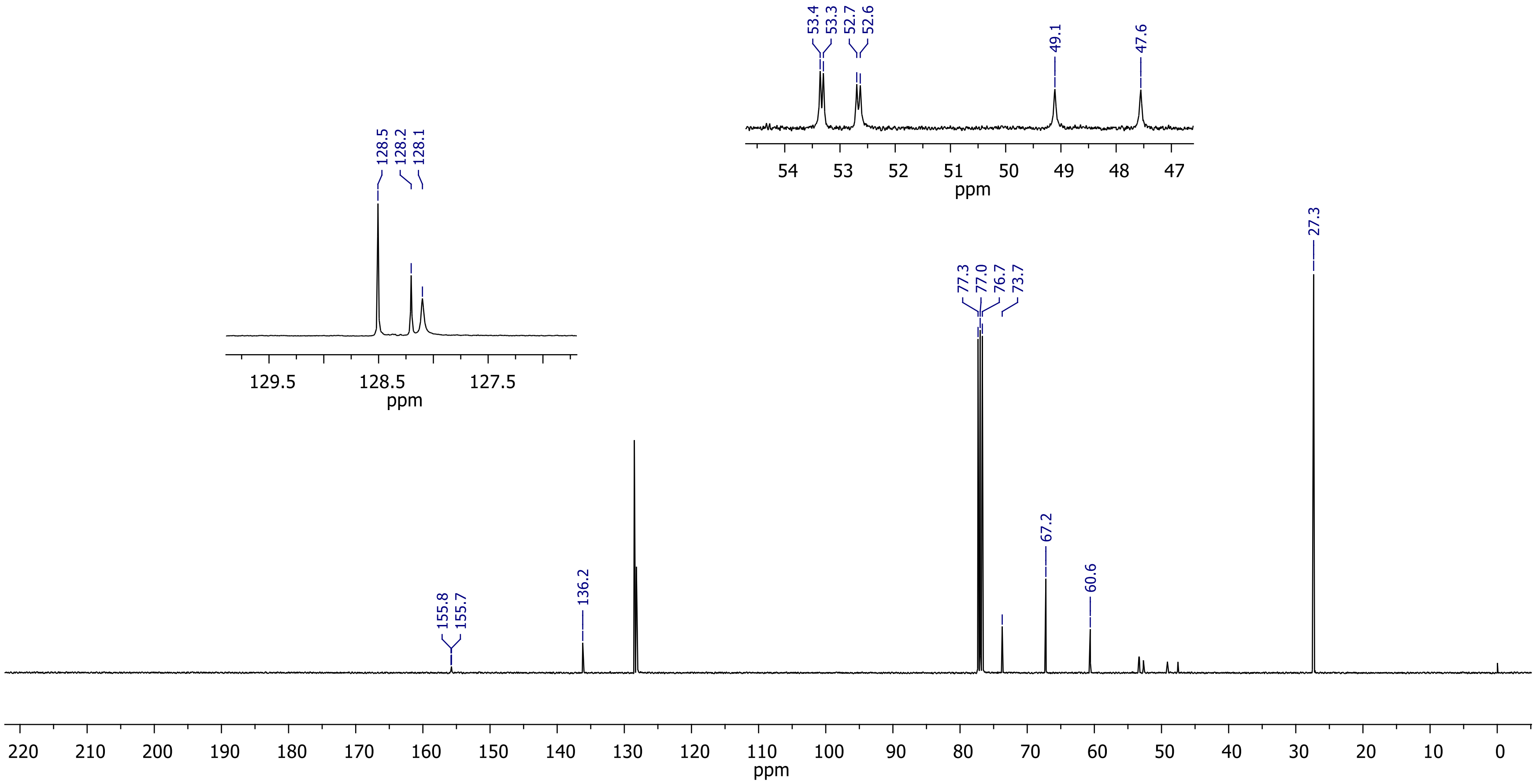

^{31}P NMR [162 MHz/CDCl$_3$]: (*R*)-Benzyl *N*-[2-(*tert*-butoxy)-1-(dimethoxyphosphoryl)ethyl]carbamate (**3j**)

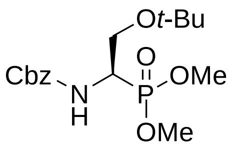

3j

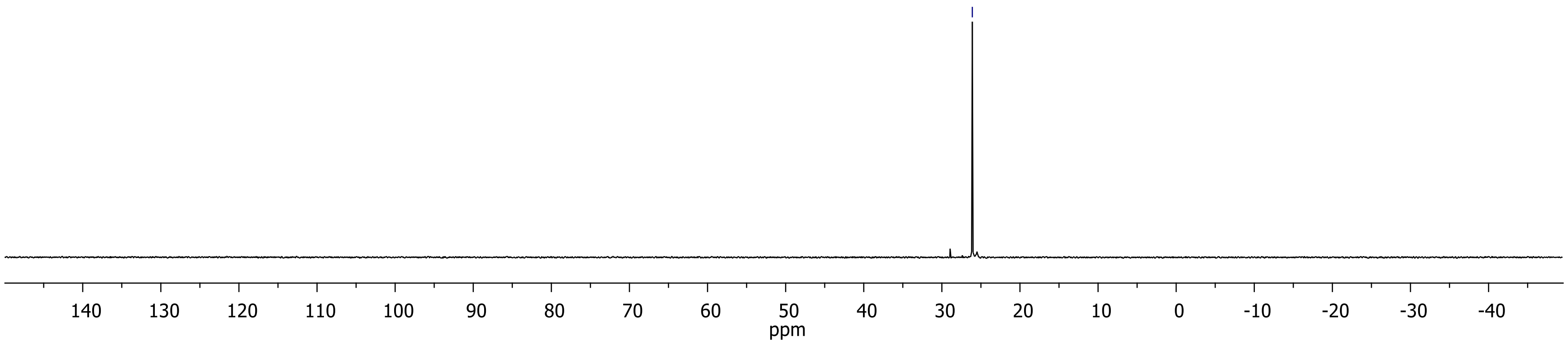

HRMS: (*R*)-Benzyl *N*-[2-(*tert*-butoxy)-1-(dimethoxyphosphoryl)ethyl]carbamate (**3j**)

Tolerance = 10.0 PPM / DBE: min = -20.0, max = 100.0
Element prediction: Off
Number of isotope peaks used for i-FIT = 2
Monoisotopic Mass, Even Electron Ions
57 formula(e) evaluated with 1 results within limits (up to 5 closest results for each mass)
Elements Used:

Mass	RA	Calc. Mass	mDa	PPM	DBE	Formula	i-FIT	i-FIT Norm	Fit Conf %	C	H	N	O	Na	P
382.1398	100.00	382.1395	0.3	0.8	4.5	C16 H26 N O6 Na P	508.8	n/a	n/a	16	26	1	6	1	1

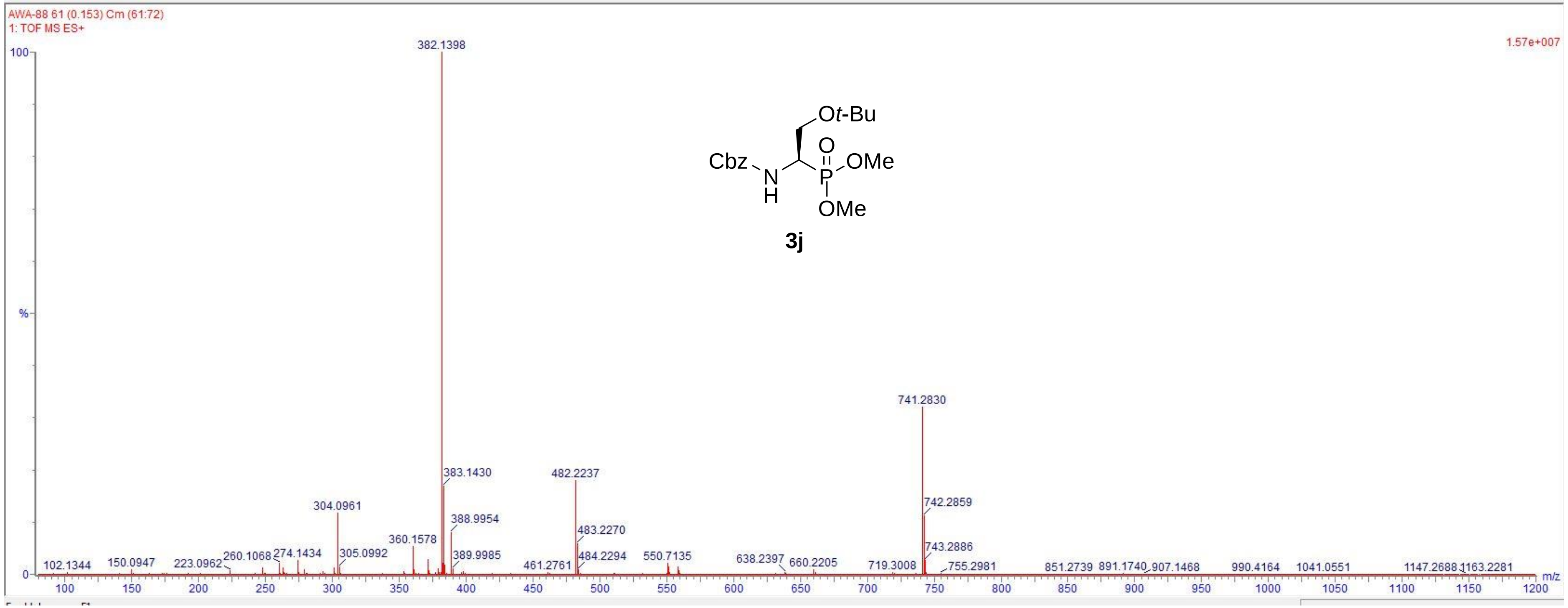

3j

^{1}H NMR [400 MHz/$CDCl_3$/TMS]: (*R*)-*tert*-Butyl *N*-[2-(benzyloxy)-1-(dimethoxyphosphoryl)ethyl]carbamate (**3k**)

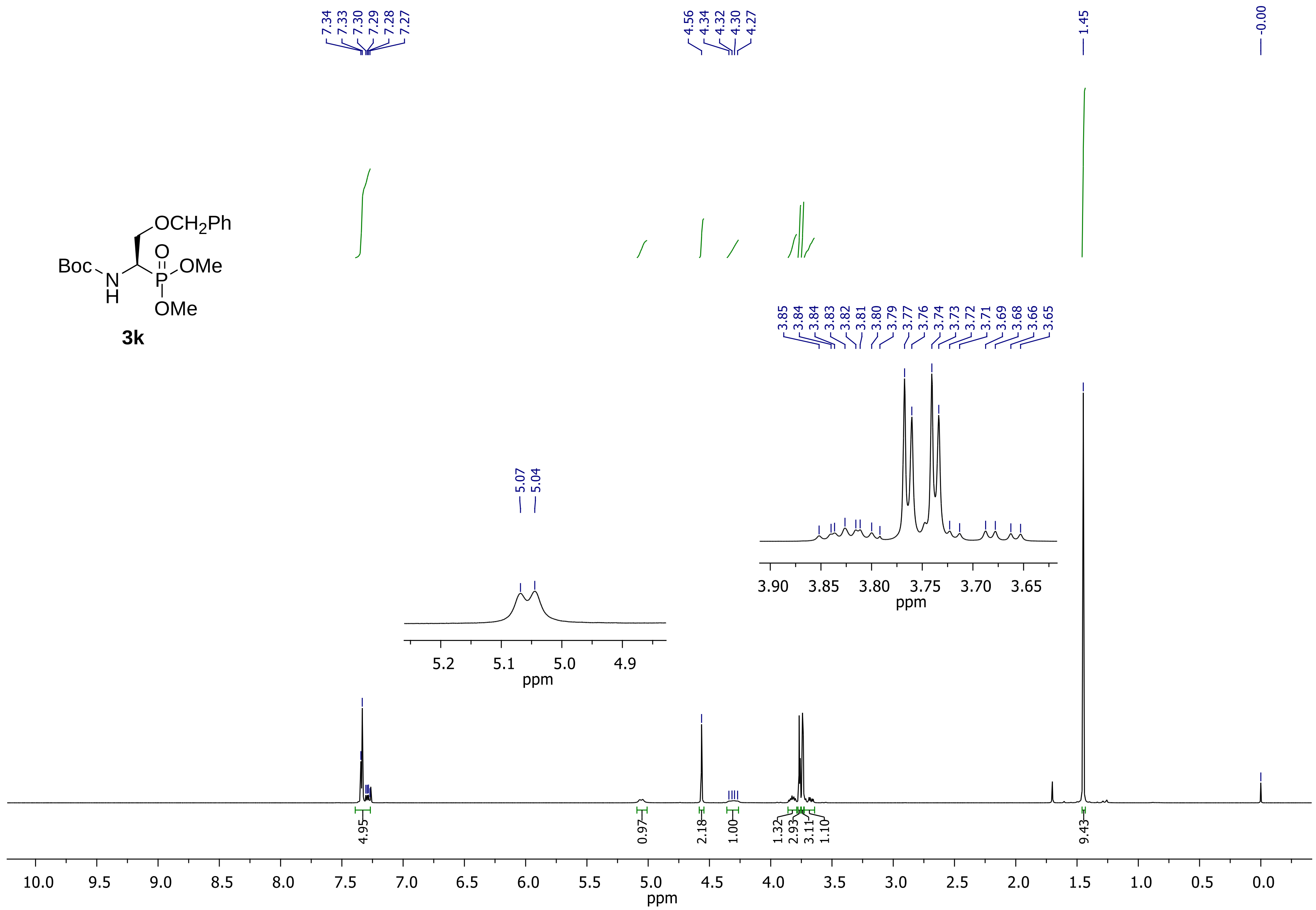

^{13}C NMR [100 MHz/$CDCl_3$]: (*R*)-*tert*-Butyl *N*-[2-(benzyloxy)-1-(dimethoxyphosphoryl)ethyl]carbamate (**3k**)

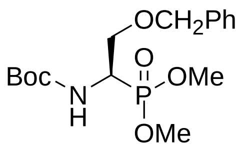

3k

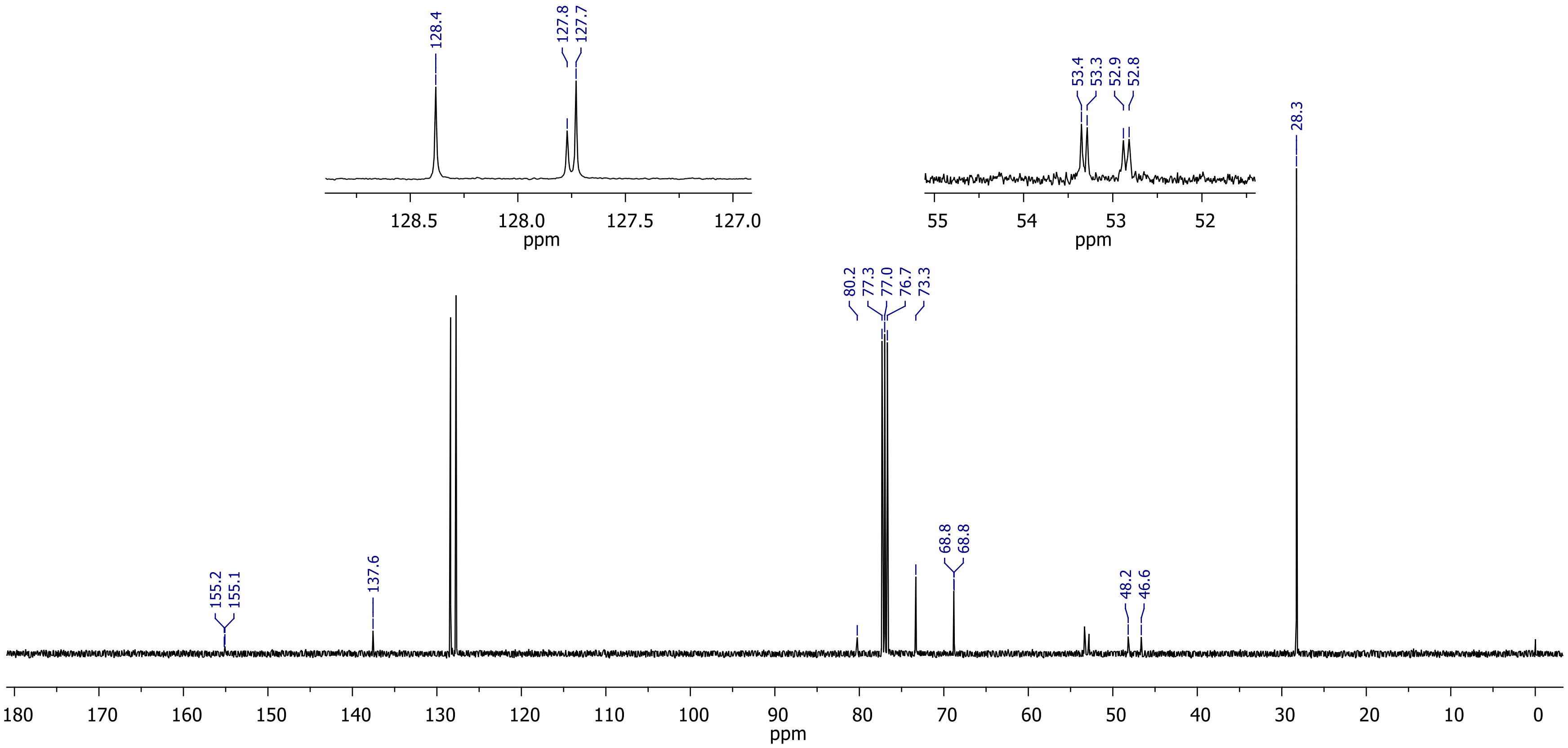

^{31}P NMR [162 MHz/CDCl$_3$]: (*R*)-*tert*-Butyl *N*-[2-(benzyloxy)-1-(dimethoxyphosphoryl)ethyl]carbamate (**3k**)

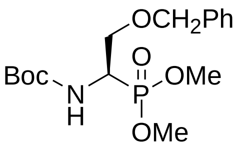

3k

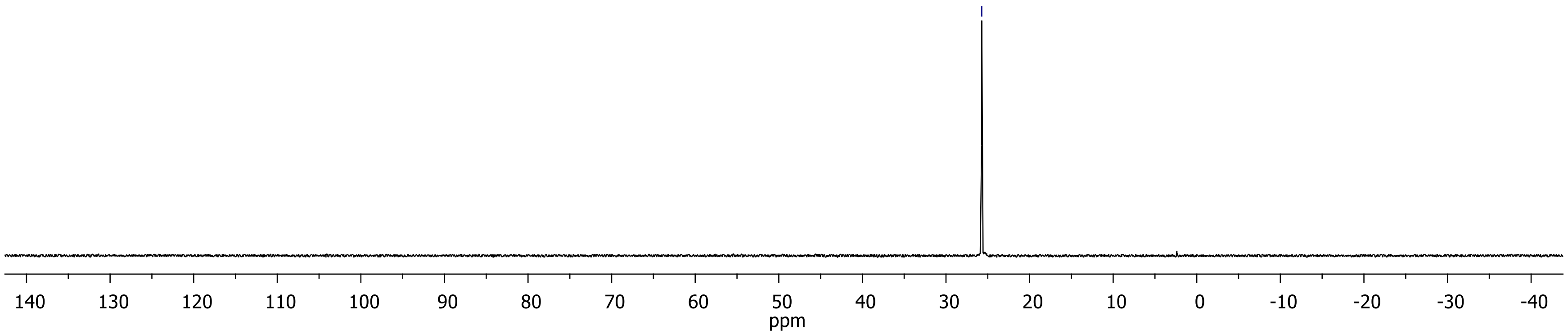

HRMS: (*R*)-*tert*-Butyl *N*-[2-(benzyloxy)-1-(dimethoxyphosphoryl)ethyl]carbamate (**3k**).

Tolerance = 10.0 PPM / DBE: min = -20.0, max = 100.0
Element prediction: Off
Number of isotope peaks used for i-FIT = 2
Monoisotopic Mass, Even Electron Ions
57 formula(e) evaluated with 1 results within limits (up to 5 closest results for each mass)
Elements Used:

Mass	RA	Calc. Mass	mDa	PPM	DBE	Formula	i-FIT	i-FIT Norm	Fit Conf %	C	H	N	O	Na	P
382.1390	100.00	382.1395	-0.5	-1.3	4.5	C16 H26 N O6 Na P	434.3	n/a	n/a	16	26	1	6	1	1

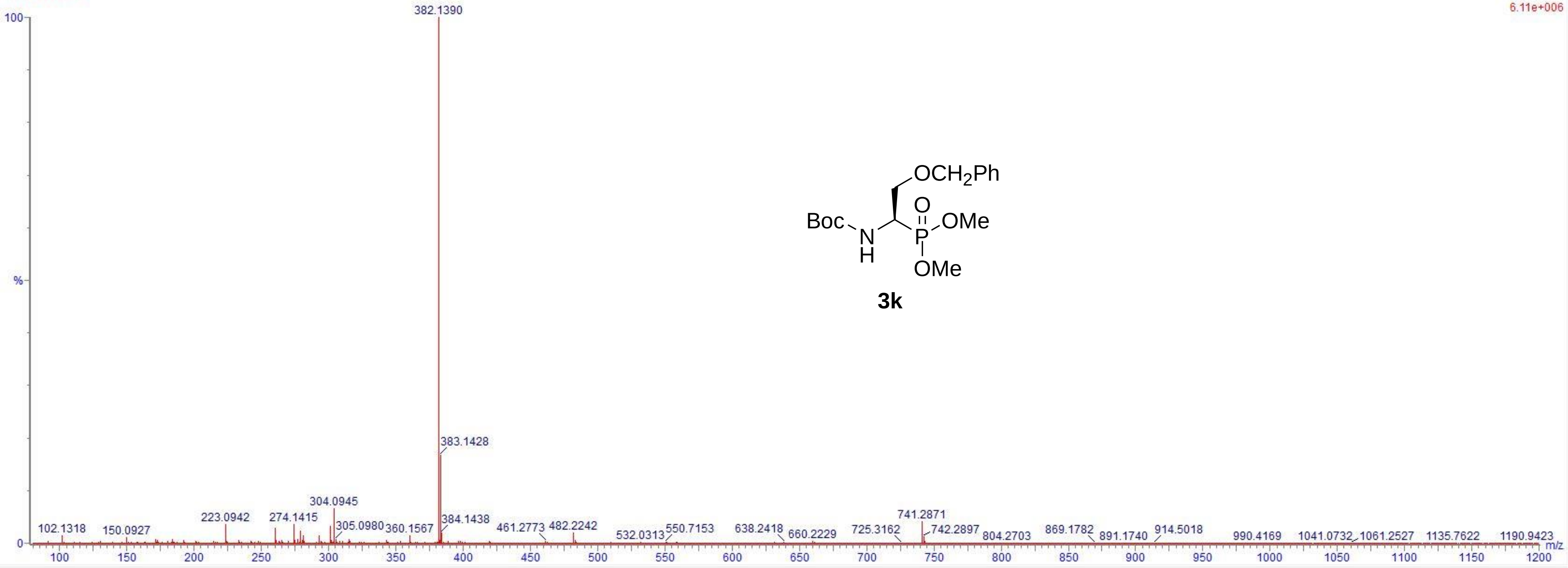

3k

HPLC conditions:
Column: Chiralpak AD-H
Eluent: 10% *i*-PrOH/hexane
Flow: 0.75mL/min
Detection: UV, 210 nm

Signal	Retention time	% Area
1	18.0	49.6
2	21.8	50.4

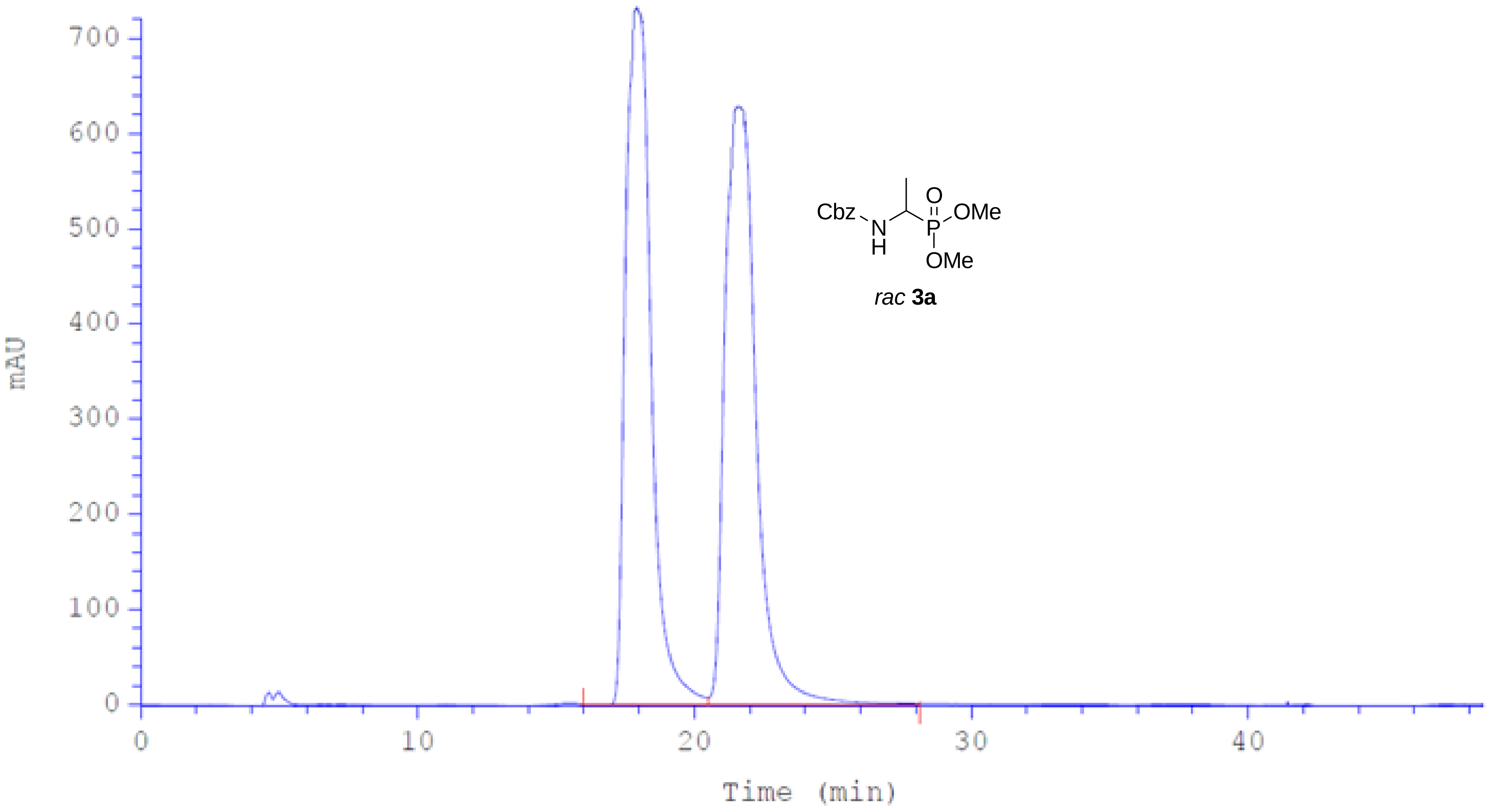

rac **3a**

HPLC conditions:
Column: Chiralpak AD-H
Eluent: 10% *i*-PrOH/hexane
Flow: 0.75mL/min
Detection: UV, 210 nm

HPLC results for the catalyst **4**		
Signal	Retention time	% Area
1	17.0	91.9
2	20.8	8.1

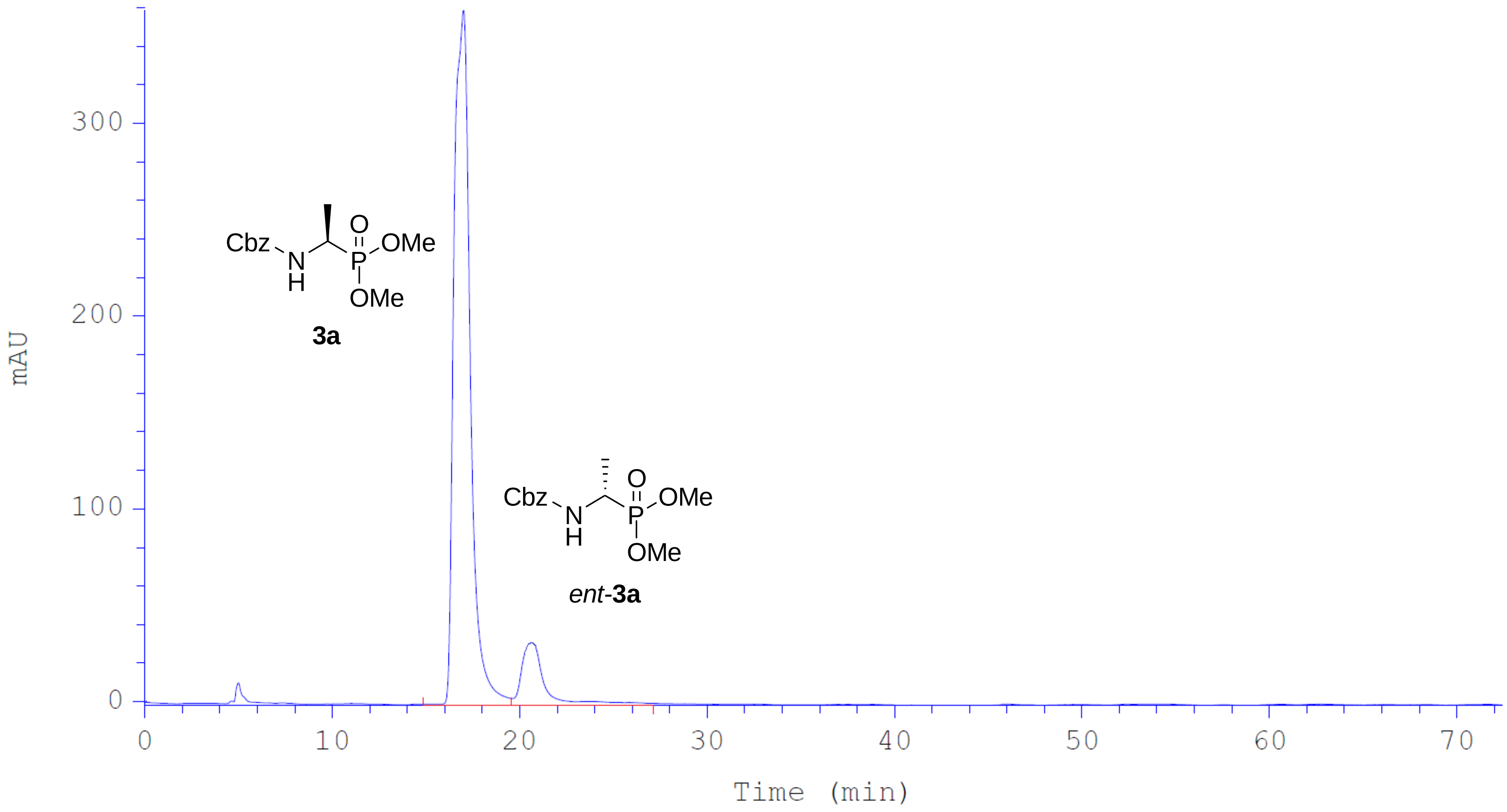

HPLC conditions:
Column: Chiralpak IA
Eluent: 10% *i*-PrOH/hexane
Flow: 0.75mL/min
Detection: UV, 210 nm

Signal	Retention time	% Area
1	16.3	49.3
2	19.0	50.7

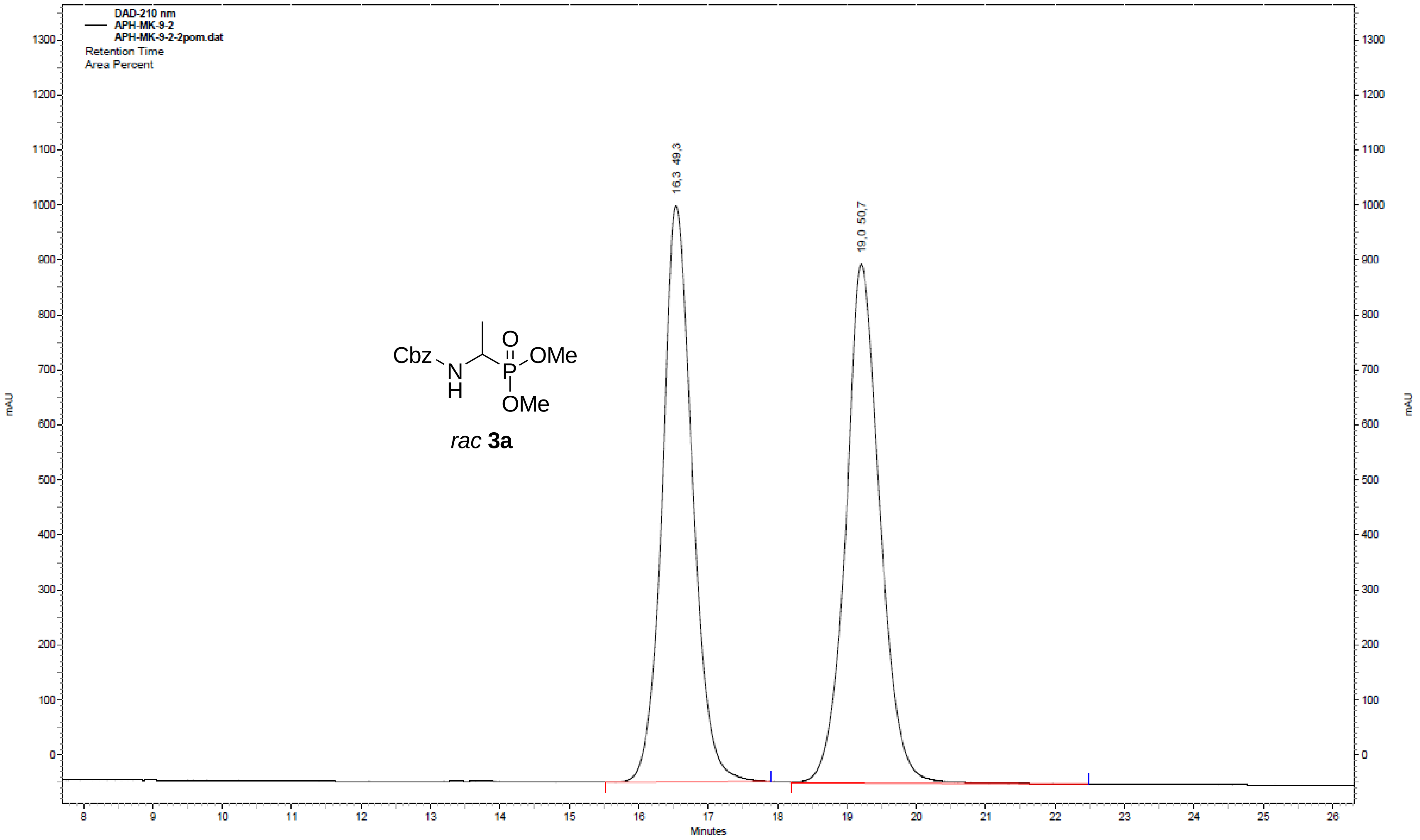

HPLC conditions:

Column: Chiralpak IA

Eluent: 10% *i*-PrOH/hexane

Flow: 0.75mL/min

Detection: UV, 210 nm

HPLC results for the catalyst **5**		
Signal	Retention time	% Area
1	15.5	91.3
2	18.8	8.7

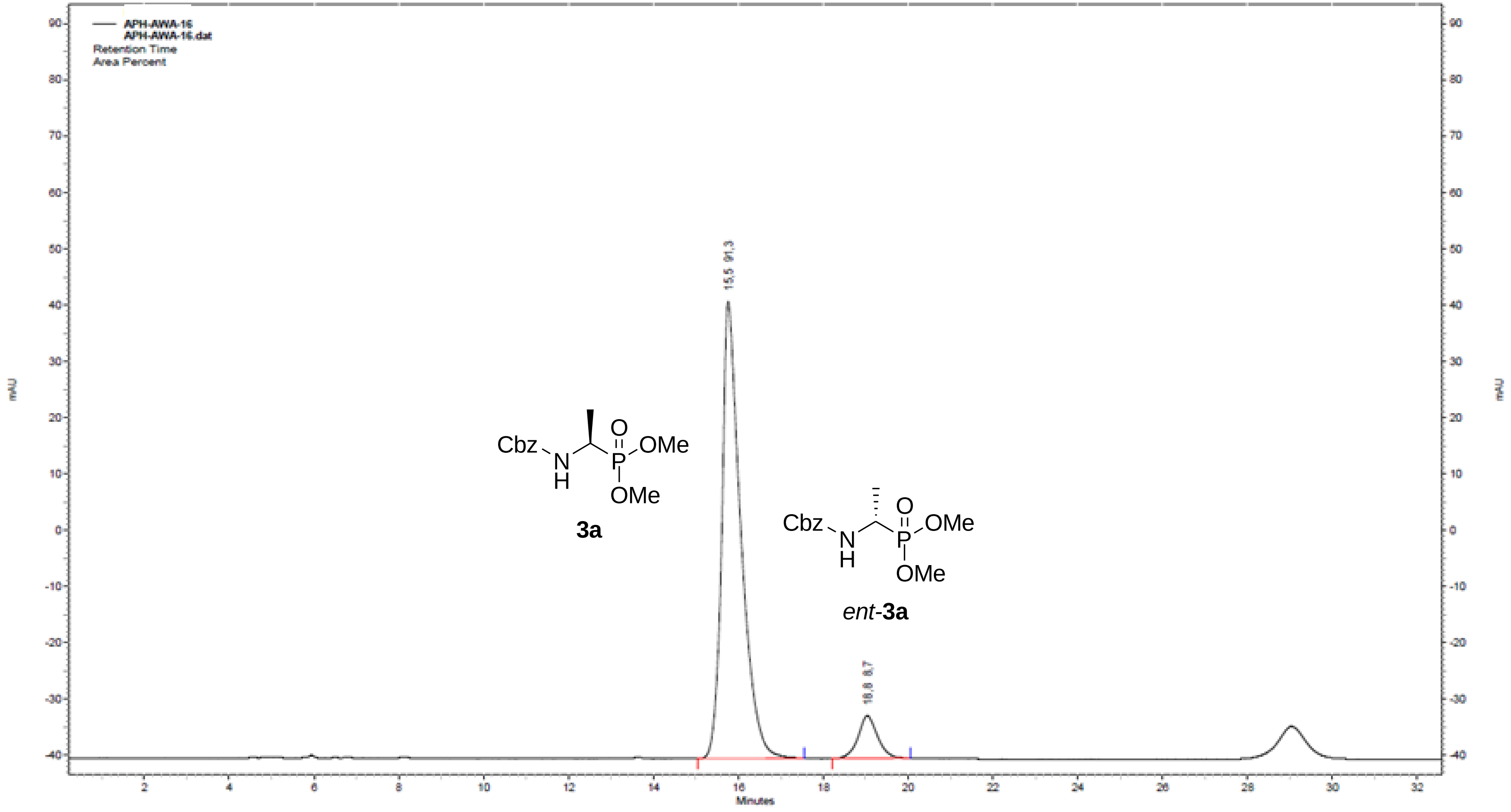

HPLC conditions:
Column: Chiralpak AD-H
Eluent: 15% *i*-PrOH/hexane
Flow: 0.8mL/min
Detection: UV, 210 nm

Signal	Retention time	% Area
1	19.7	49.2
2	27.8	50.8

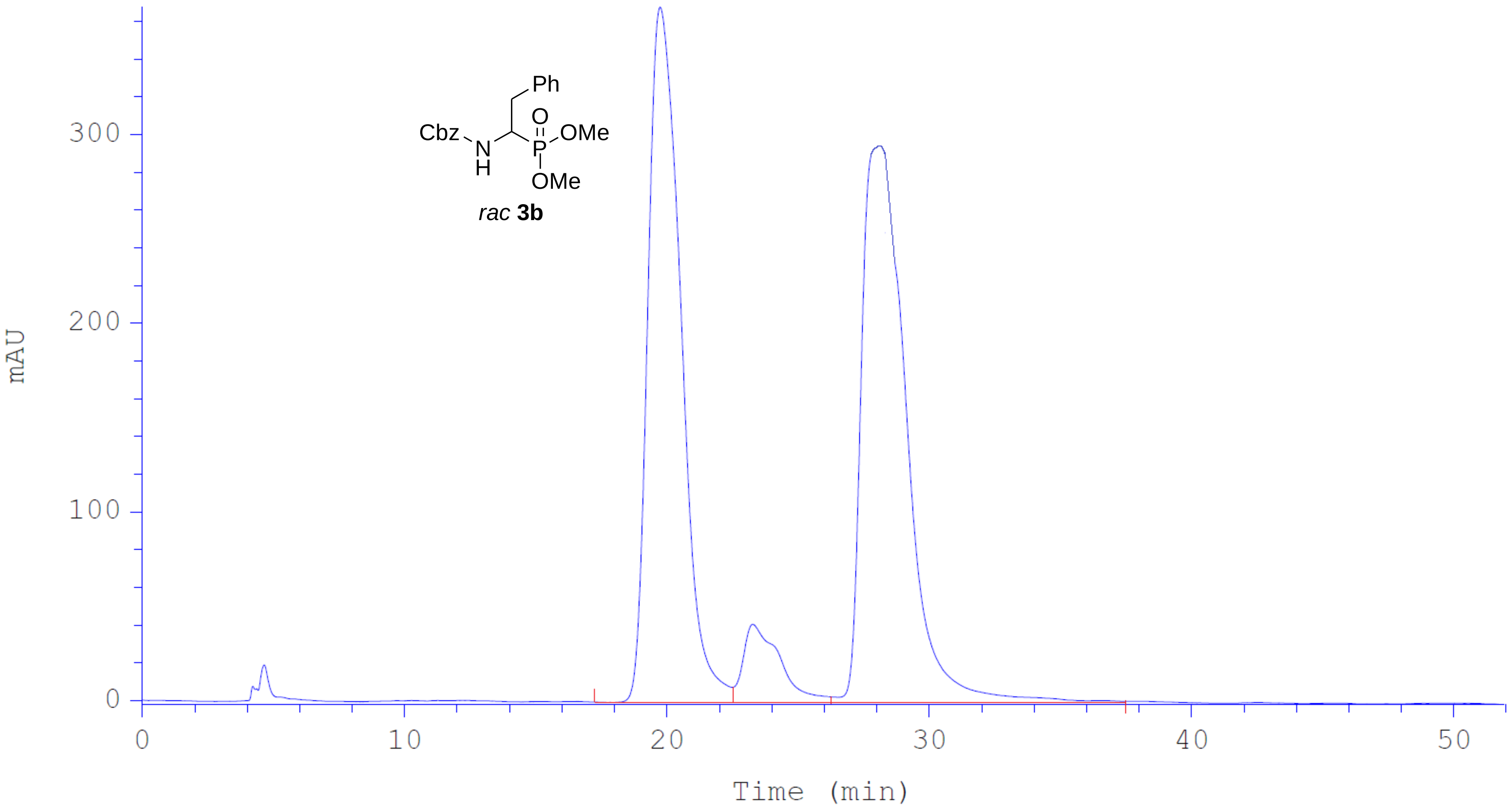

HPLC conditions:
Column: Chiralpak AD-H
Eluent: 15% *i*-PrOH/hexane
Flow: 0.8mL/min
Detection: UV, 210 nm

HPLC results for the catalyst **4**		
Signal	Retention time	% Area
1	20.6	86.3
2	28.7	13.7

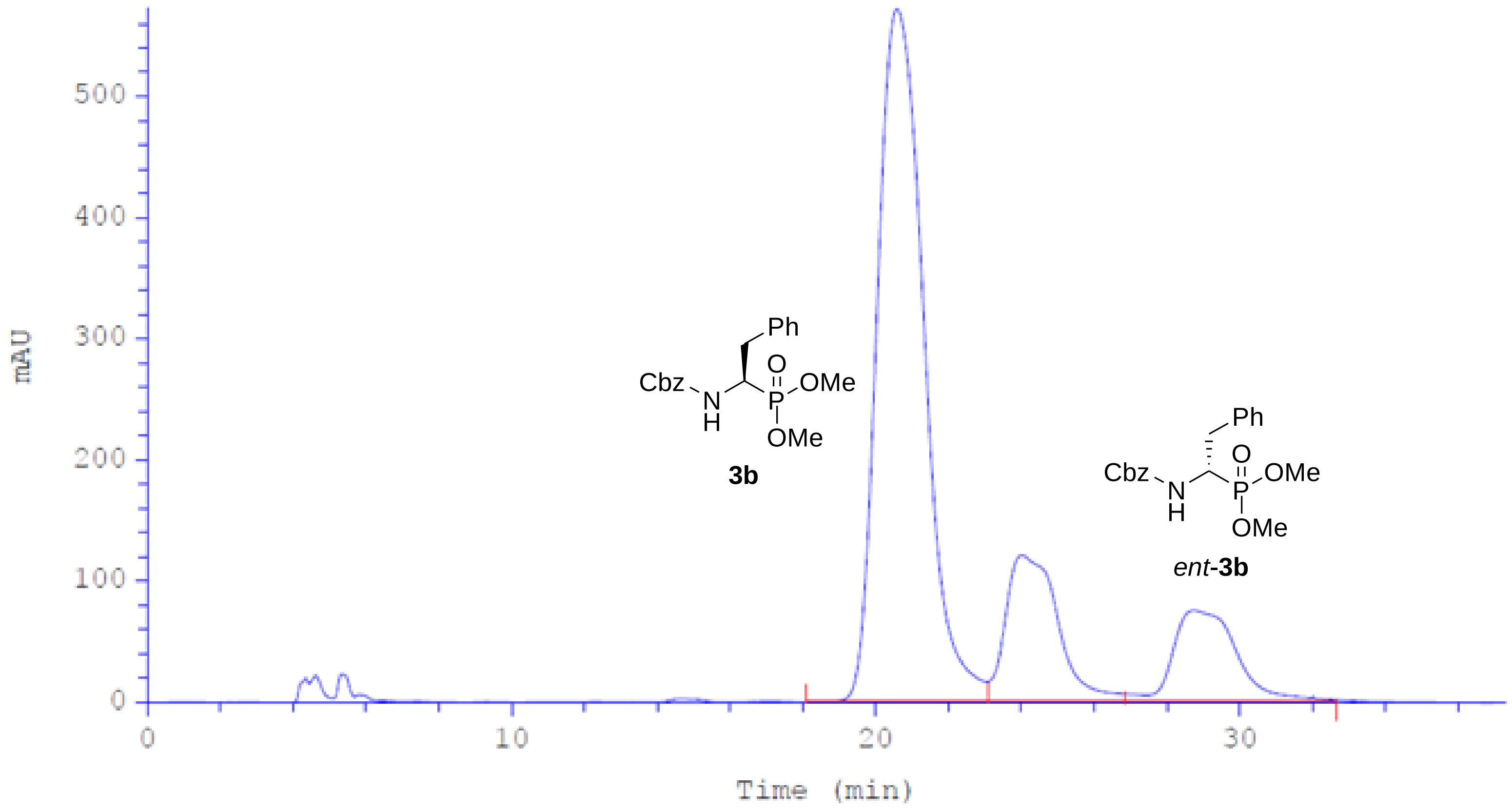

HPLC conditions:
Column: Chiralpak AD-H
Eluent: 15% *i*-PrOH/hexane
Flow: 0.5 mL/min
Detection: UV, 210 nm

Signal	Retention time	% Area
1	19.5	51.0
2	26.9	49.0

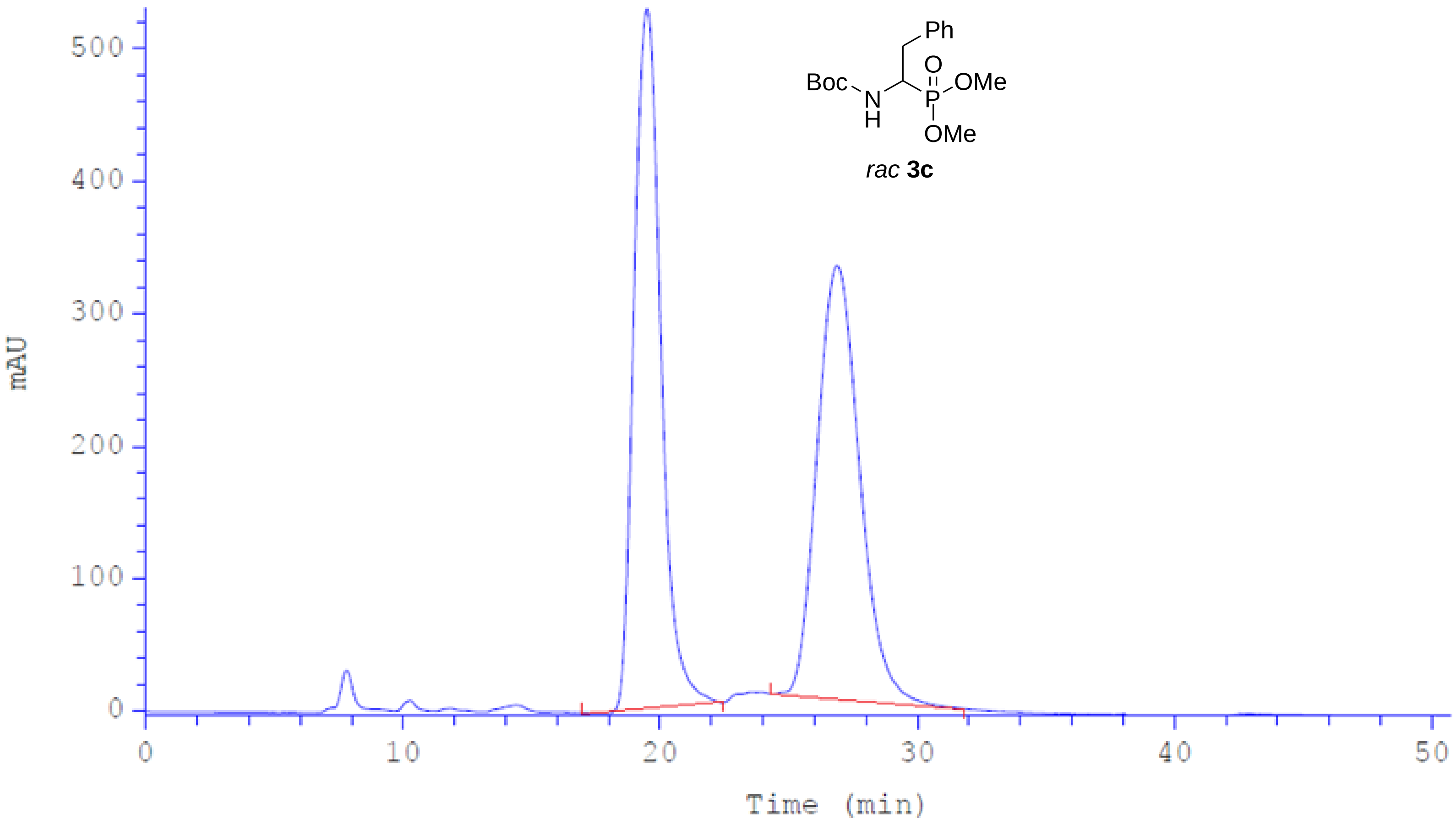

rac **3c**

HPLC conditions:

Column: Chiralpak AD-H

Eluent: 15% *i*-PrOH/hexane

Flow: 0.5 mL/min

Detection: UV, 210 nm

HPLC results for the catalyst **4**		
Signal	Retention time	% Area
1	19.1	14.0
2	26.4	86.0

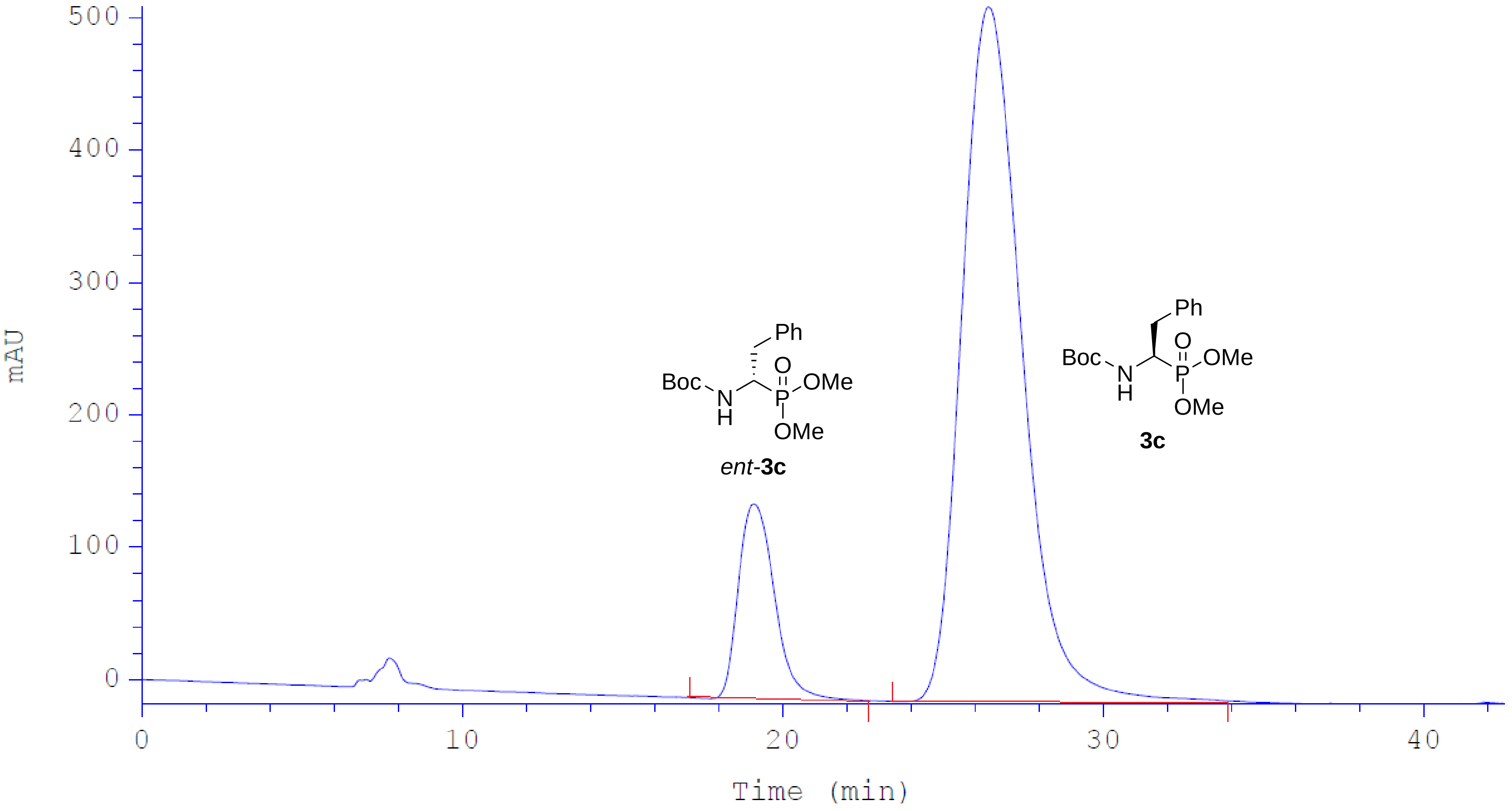

HPLC conditions:
Column: Chiralpak AD-H
Eluent: 15% *i*-PrOH/hexane
Flow: 0.5 mL/min
Detection: UV, 210 nm

HPLC results for the catalyst **5**		
Signal	Retention time	% Area
1	19.6	19.5
2	27.0	80.5

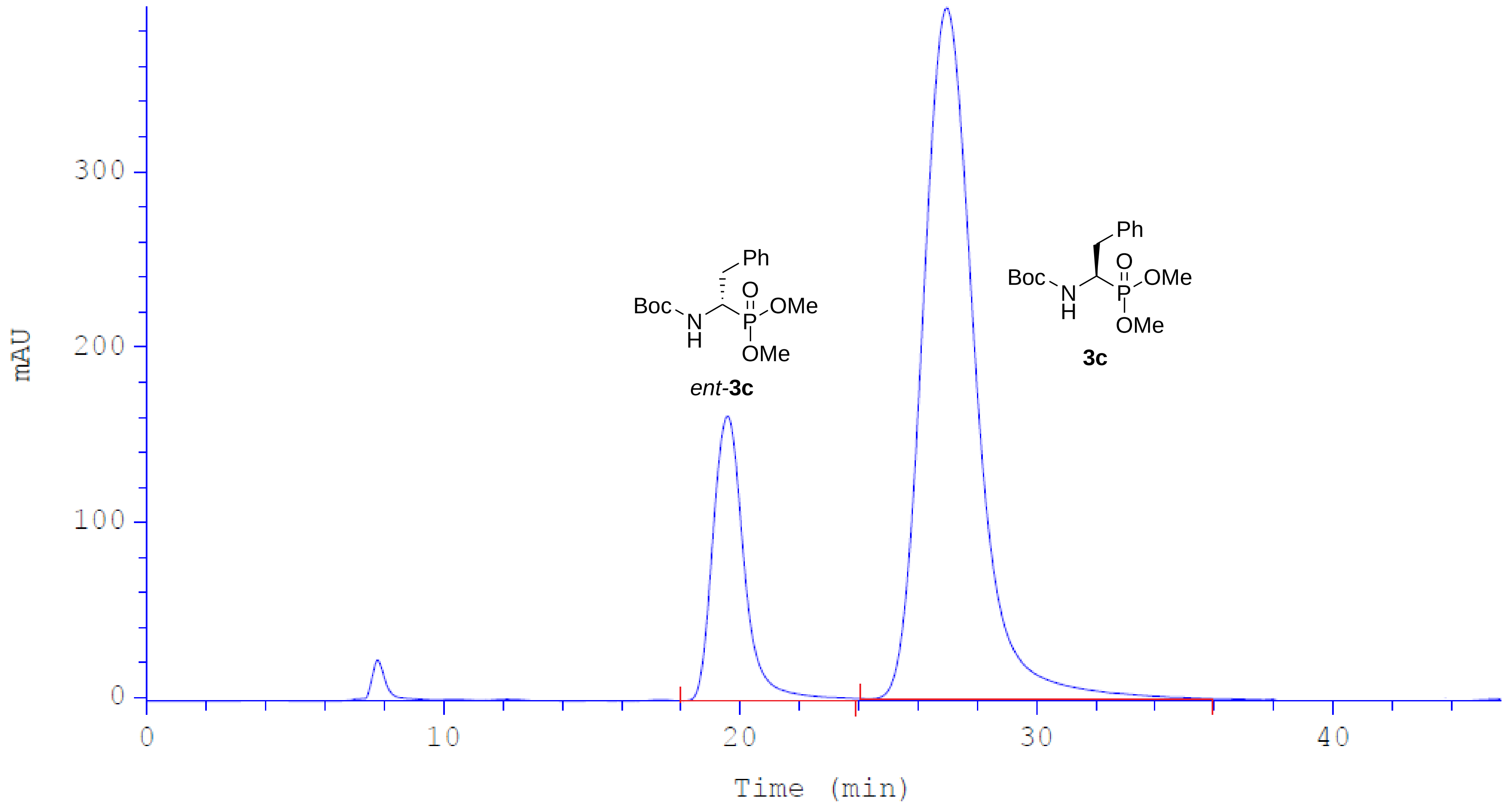

HPLC conditions:
Column: Chiralpak AD-H
Eluent: 10% *i*-PrOH/hexane
Flow: 0.75 mL/min
Detection: UV, 210 nm

Signal	Retention time	% Area
1	14.1	50.5
2	22.9	49.5

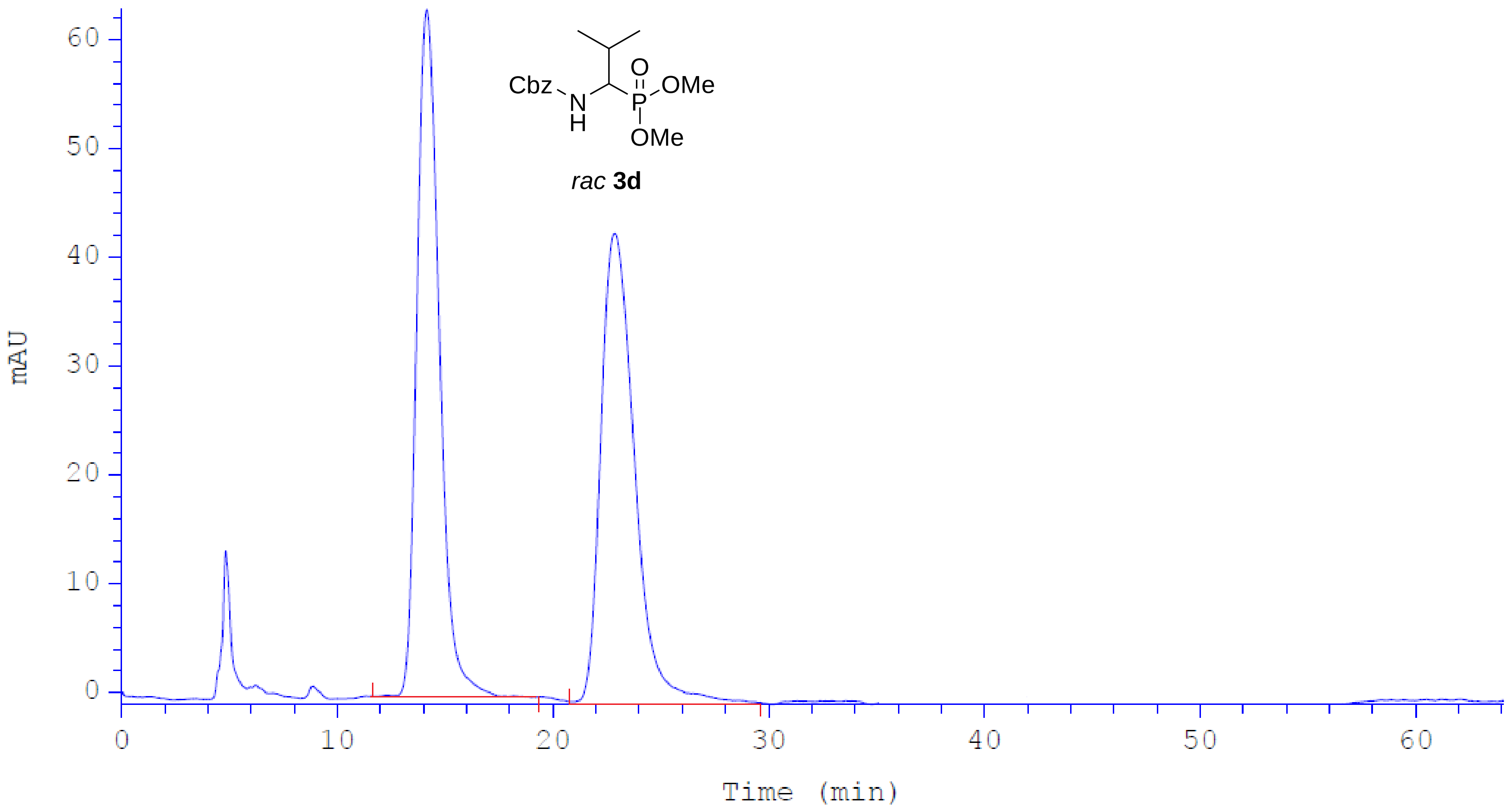

rac **3d**

HPLC conditions:
Column: Chiralpak AD-H
Eluent: 10% *i*-PrOH/hexane
Flow: 0.75 mL/min
Detection: UV, 210 nm

HPLC results for the catalyst **4**		
Signal	Retention time	% Area
1	14.5	77.9
2	23.0	22.1

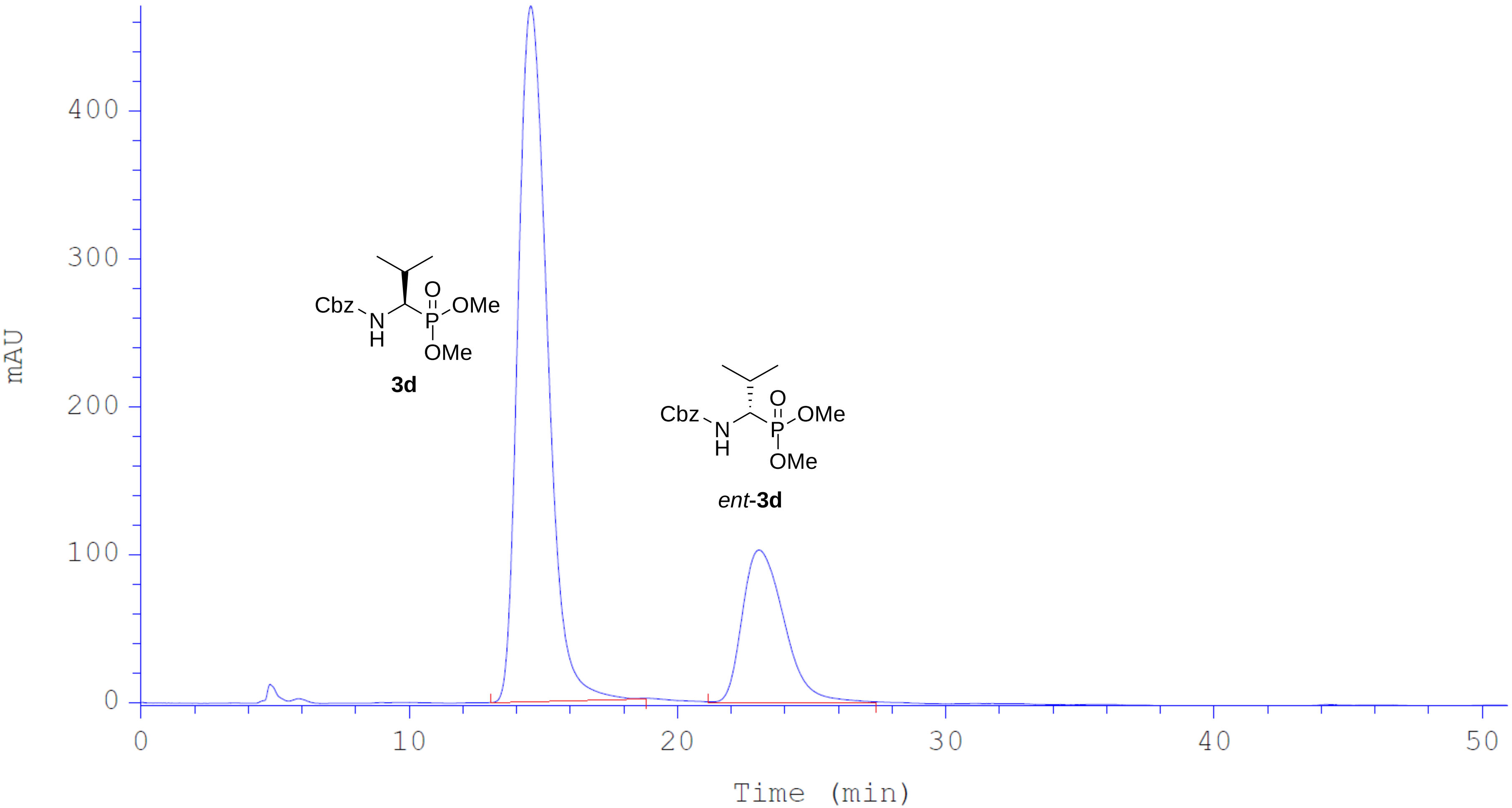

HPLC conditions:
Column: Chiralpak IA
Eluent: 10% *i*-PrOH/hexane
Flow: 0.75 mL/min
Detection: UV, 210 nm

Signal	Retention time	% Area
1	13.5	48.9
2	19.4	51.1

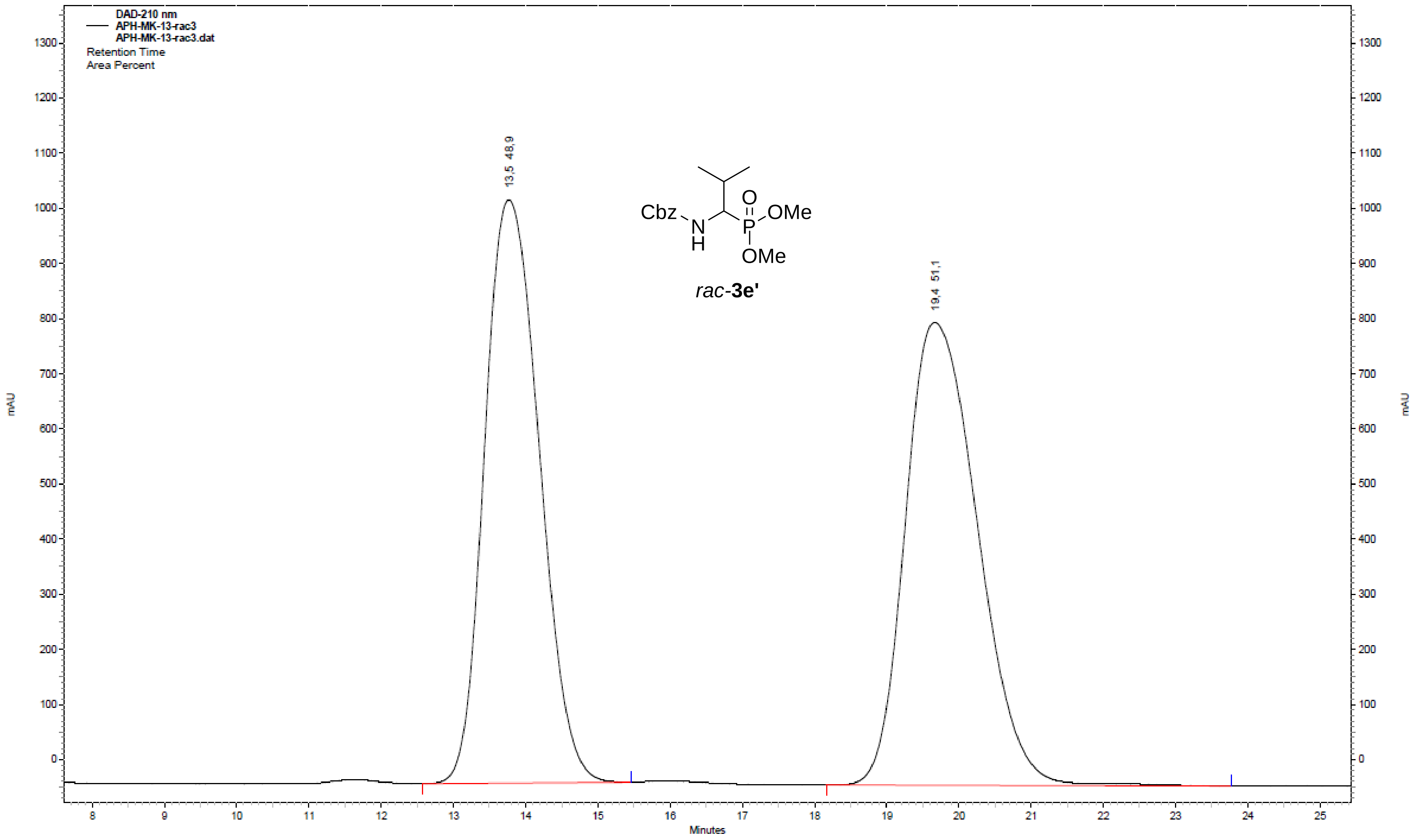

HPLC conditions:

Column: Chiralpak IA

Eluent: 10% *i*-PrOH/hexane

Flow: 0.75 mL/min

Detection: UV, 210 nm

HPLC results for the catalyst **4**		
Signal	Retention time	% Area
1	13.3	61.7
2	19.8	38.3

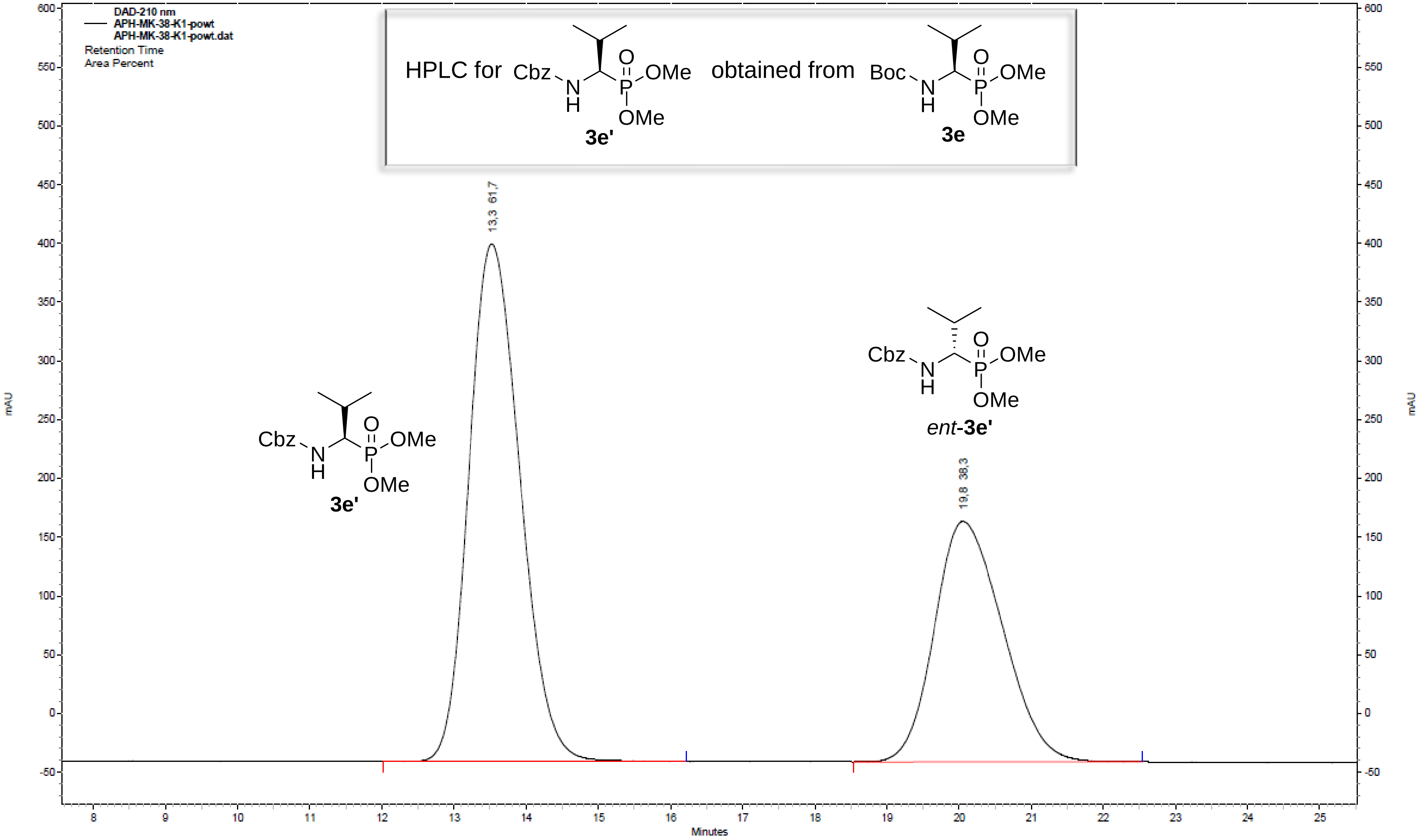

HPLC conditions:

Column: Chiralpak IA

Eluent: 15% *i*-PrOH/hexane

Flow: 0.75mL/min

Detection: UV, 254 nm

Signal	Retention time	% Area
1	10.3	48.4
2	13.6	51.2

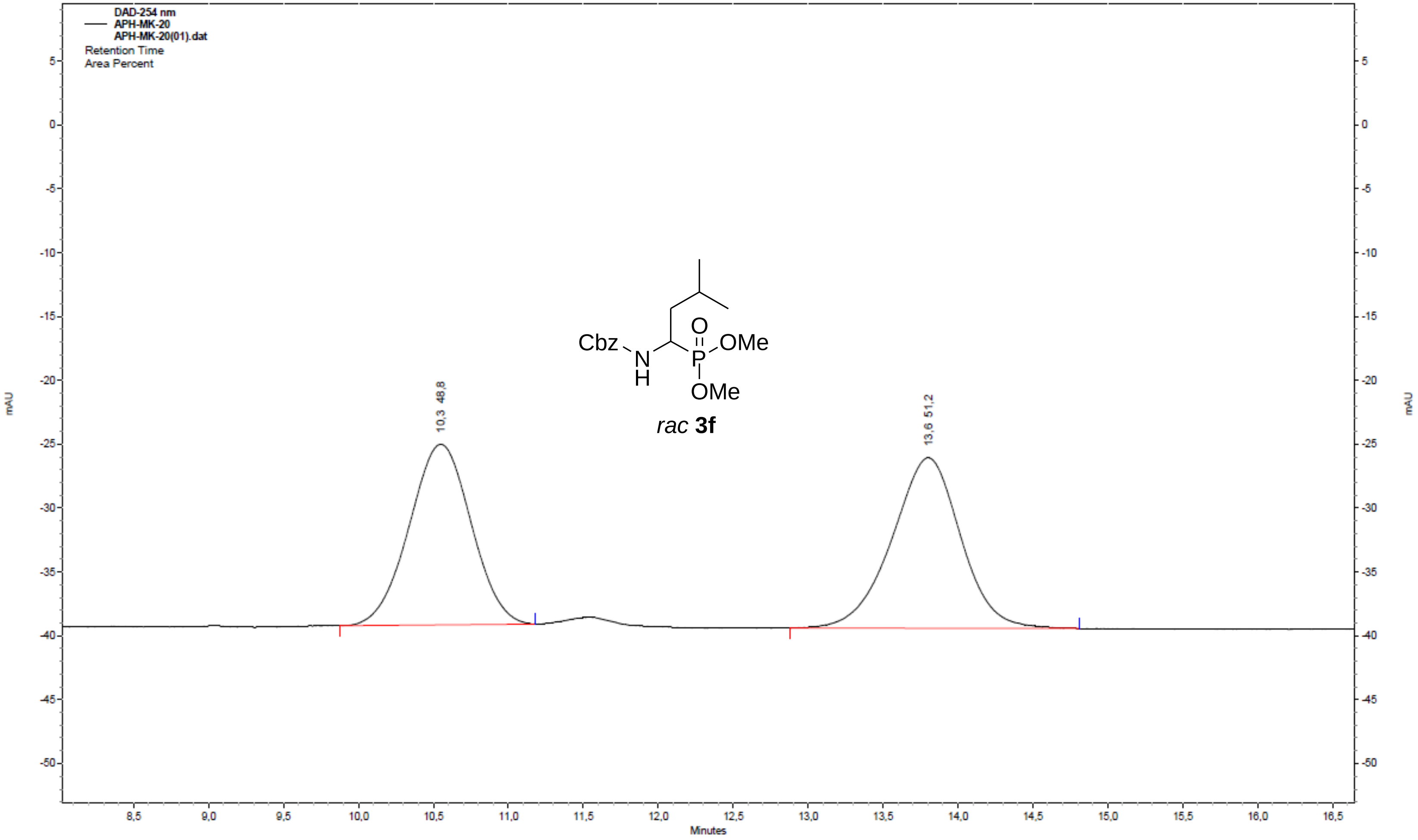

HPLC conditions:
Column: Chiralpak IA
Eluent: 15% *i*-PrOH/hexane
Flow: 0.75mL/min
Detection: UV, 254 nm

HPLC results for the catalyst **4**		
Signal	Retention time	% Area
1	10.1	90.1
2	13.4	9.9

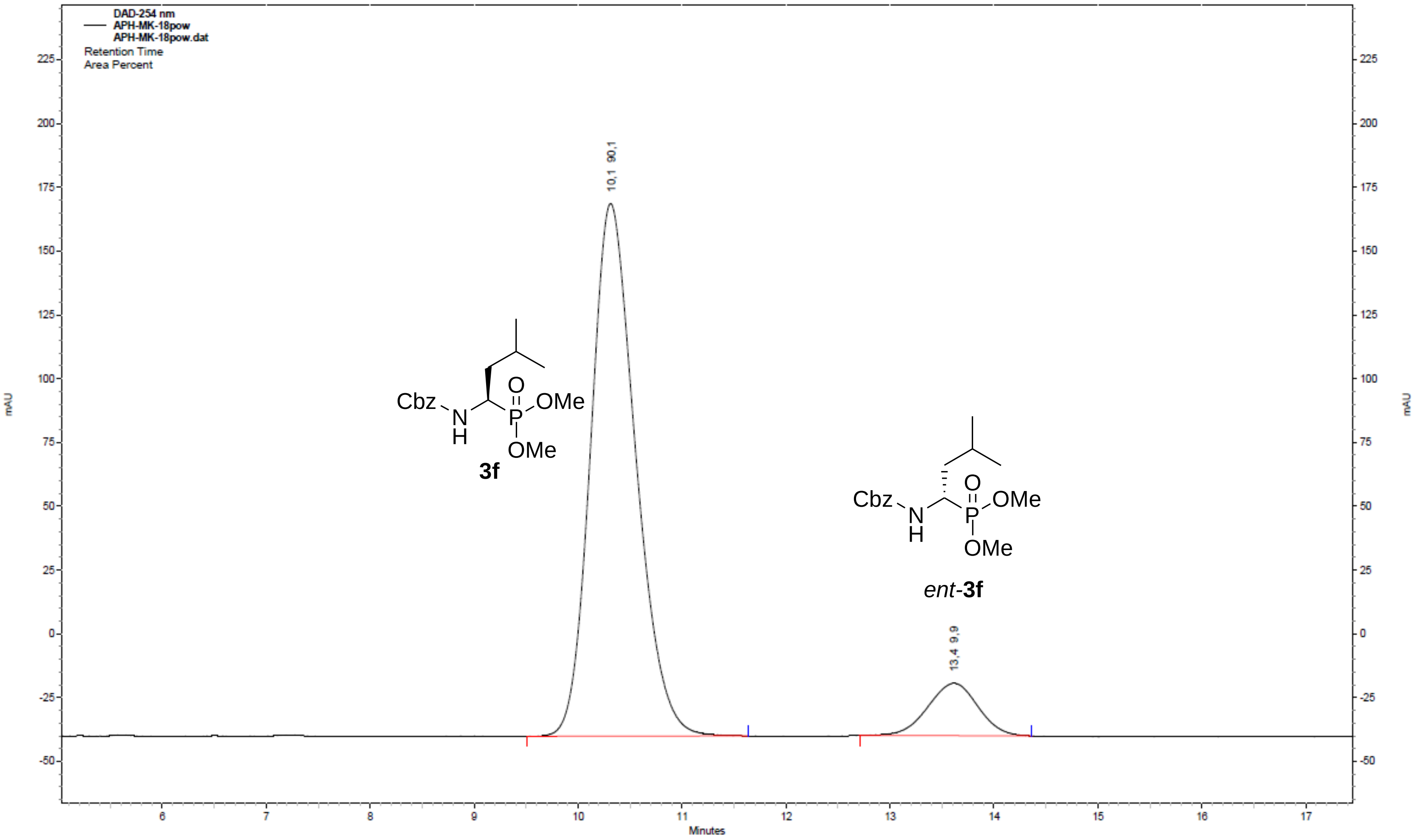

HPLC conditions:

Column: Chiralpak IA

Eluent: 15% *i*-PrOH/hexane

Flow: 0.75mL/min

Detection: UV, 254 nm

HPLC results for the catalyst **5**		
Signal	Retention time	% Area
1	10.3	89.4
2	13.6	10.6

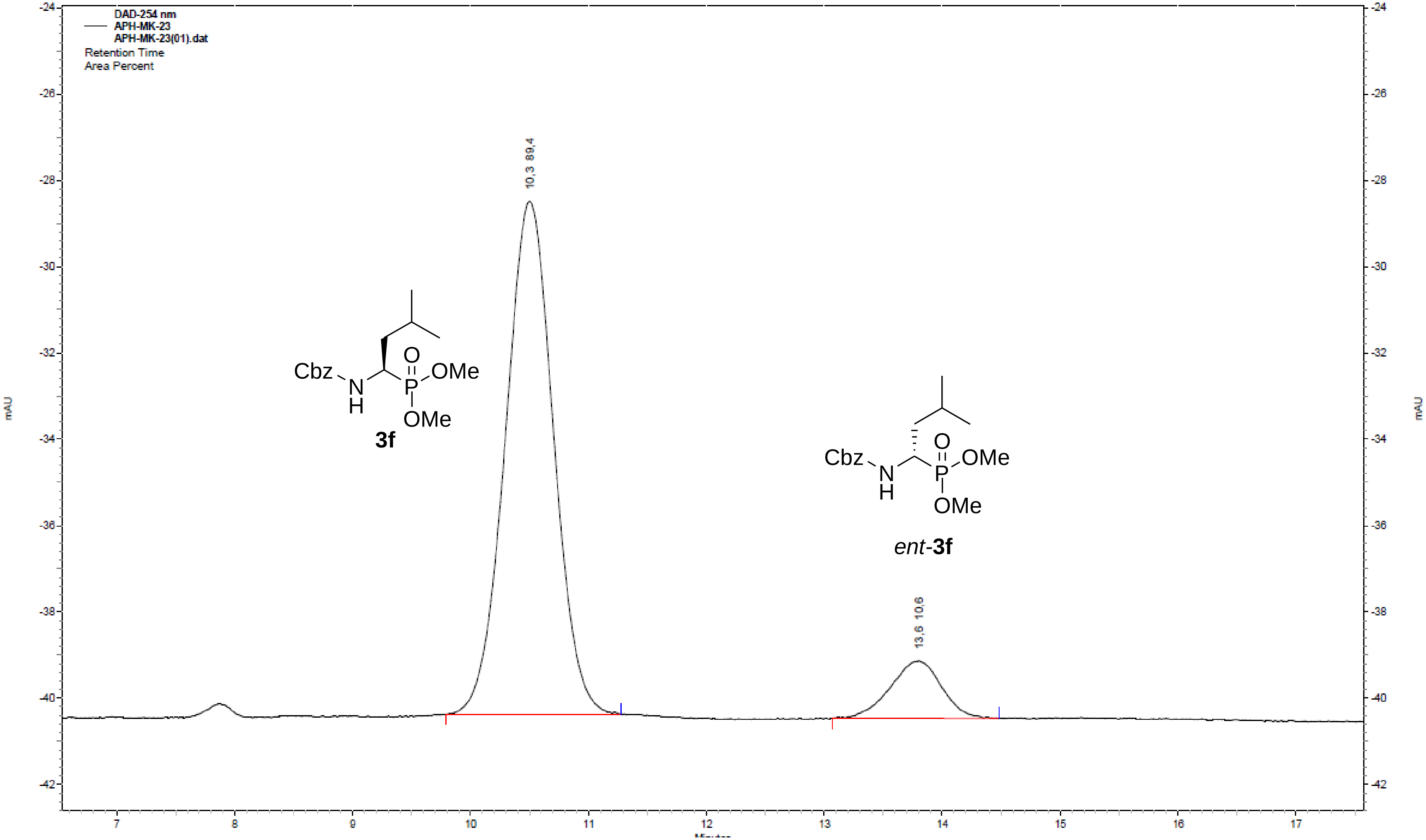

HPLC conditions:
Column: Chiralpak AD-H
Eluent: 10% *i*-PrOH/hexane
Flow: 0.75mL/min
Detection: UV, 210 nm

Signal	Retention time	% Area
1	17.3	49.6
2	23.8	50.4

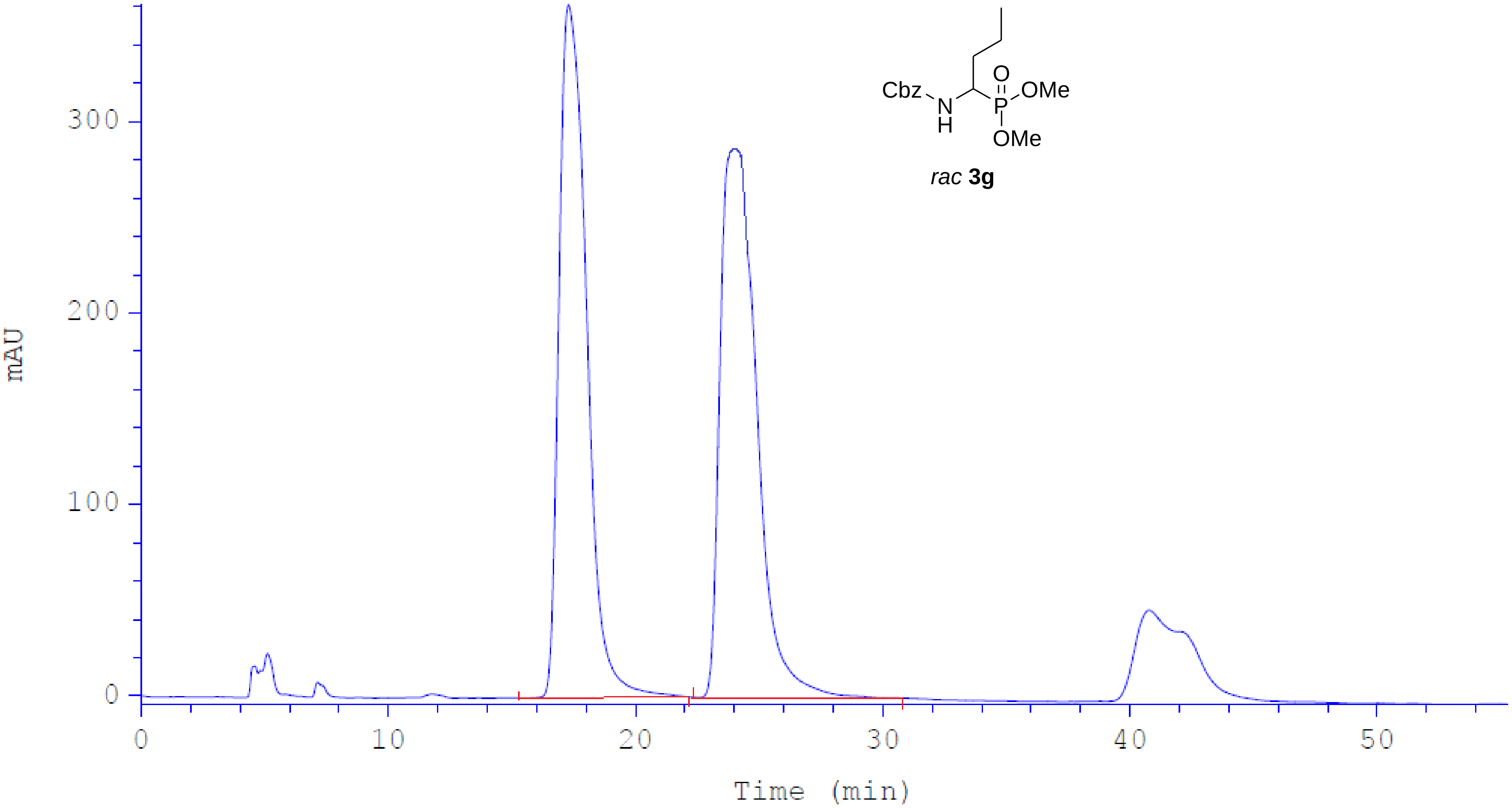

HPLC conditions:
Column: Chiralpak AD-H
Eluent: 10% *i*-PrOH/hexane
Flow: 0.75mL/min
Detection: UV, 210 nm

HPLC results for the catalyst **4**		
Signal	Retention time	% Area
1	18.1	89.8
2	24.9	10.2

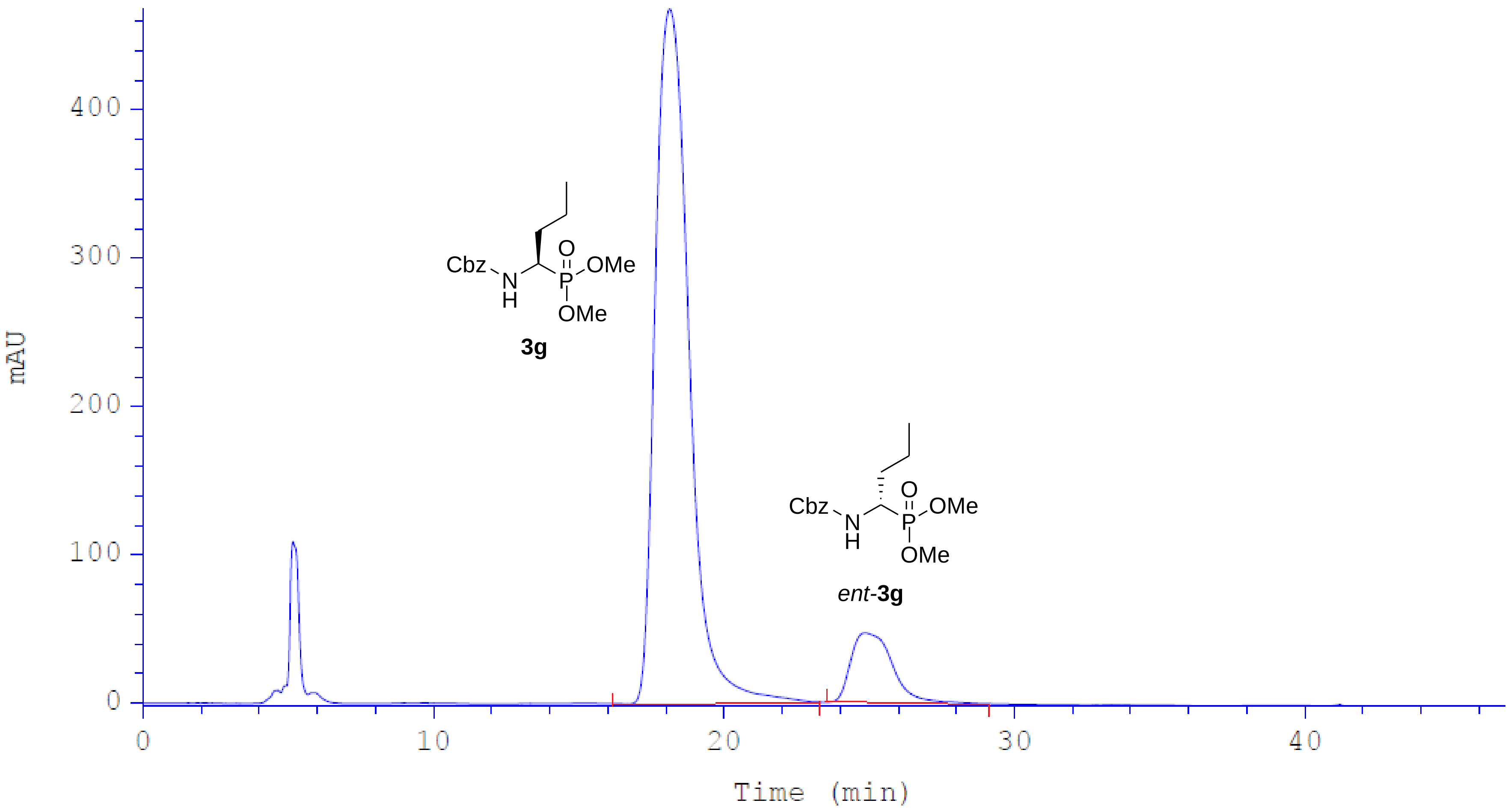

HPLC conditions:
Column: Chiralpak AD-H
Eluent: 10% *i*-PrOH/hexane
Flow: 0.75mL/min
Detection: UV, 210 nm

Signal	Retention time	% Area
1	15.3	47.7
2	22.7	52.3

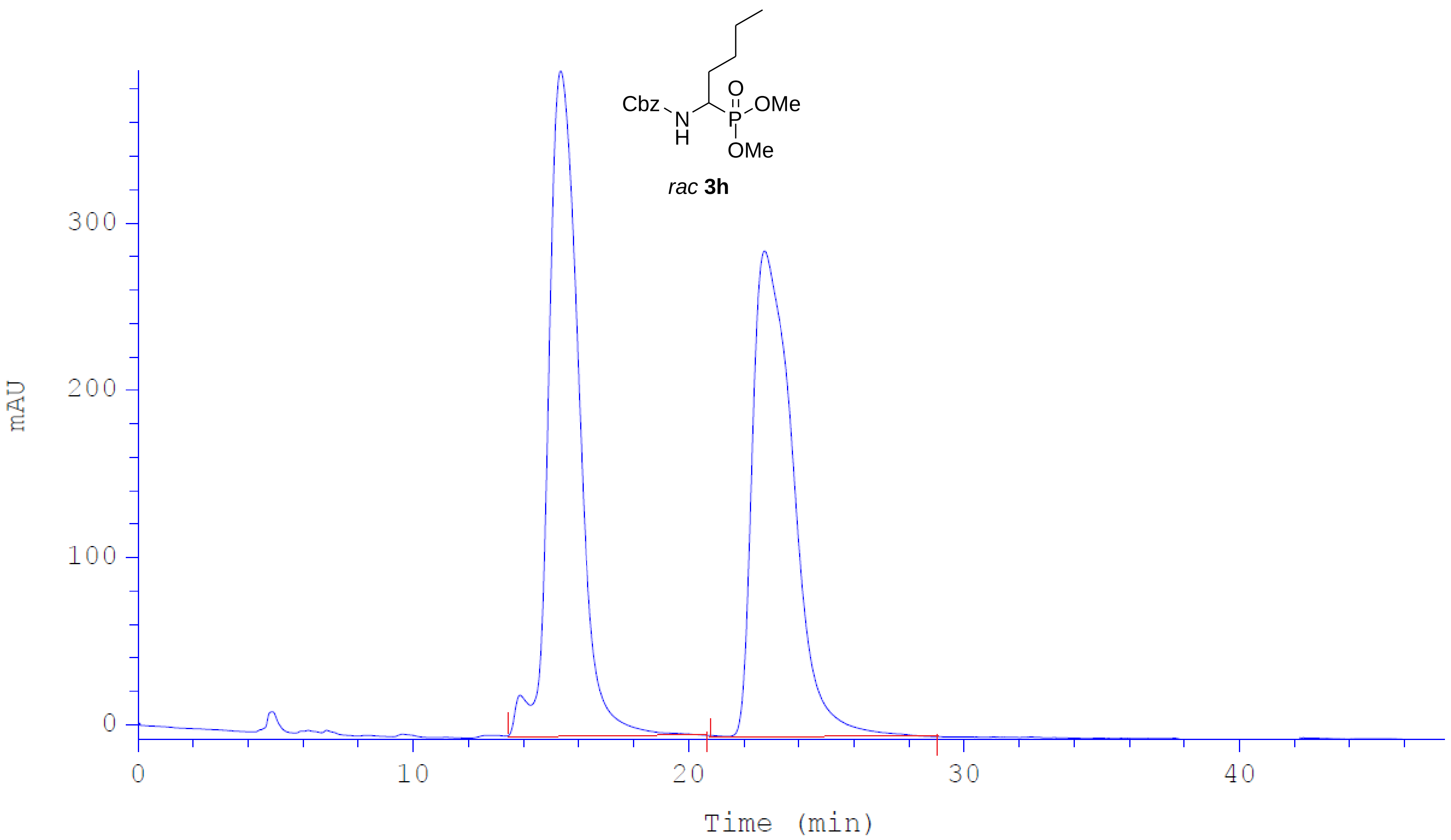

rac **3h**

HPLC conditions:
Column: Chiralpak AD-H
Eluent: 10% *i*-PrOH/hexane
Flow: 0.75mL/min
Detection: UV, 210 nm

HPLC results for the catalyst **4**		
Signal	Retention time	% Area
1	15.7	95.9
2	23.2	4.1

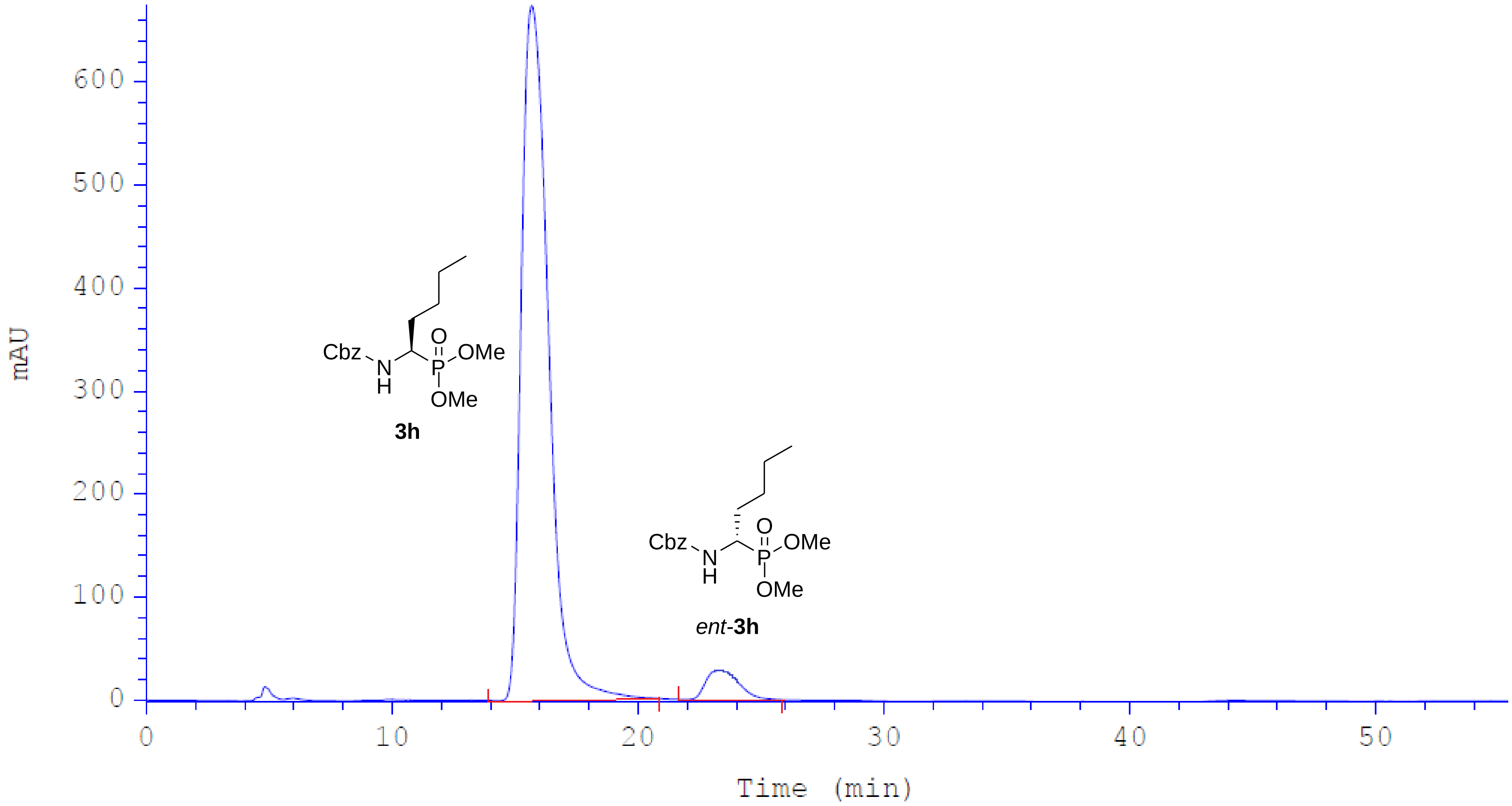

HPLC conditions:
Column: Chiralpak IA
Eluent: 15% *i*-PrOH/hexane
Flow: 0.75 mL/min
Detection: UV, 205 nm

Signal	Retention time	% Area
1	15.7	49.9
2	21.0	50.1

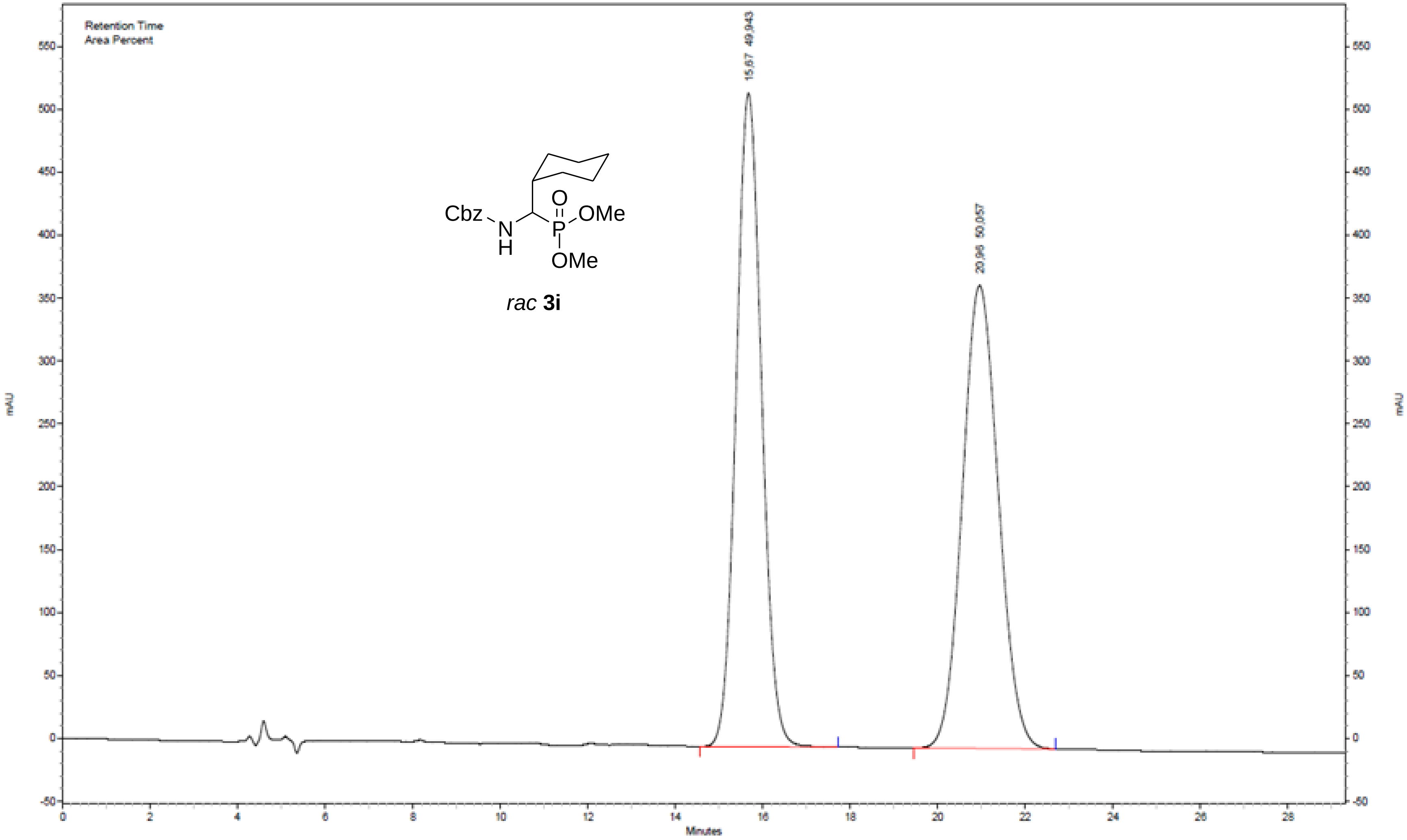

HPLC conditions:
Column: Chiralpak IA
Eluent: 15% *i*-PrOH/hexane
Flow: 0.75 mL/min
Detection: UV, 205 nm

HPLC results for the catalyst **4**		
Signal	Retention time	% Area
1	15.6	82.5
2	20.9	17.5

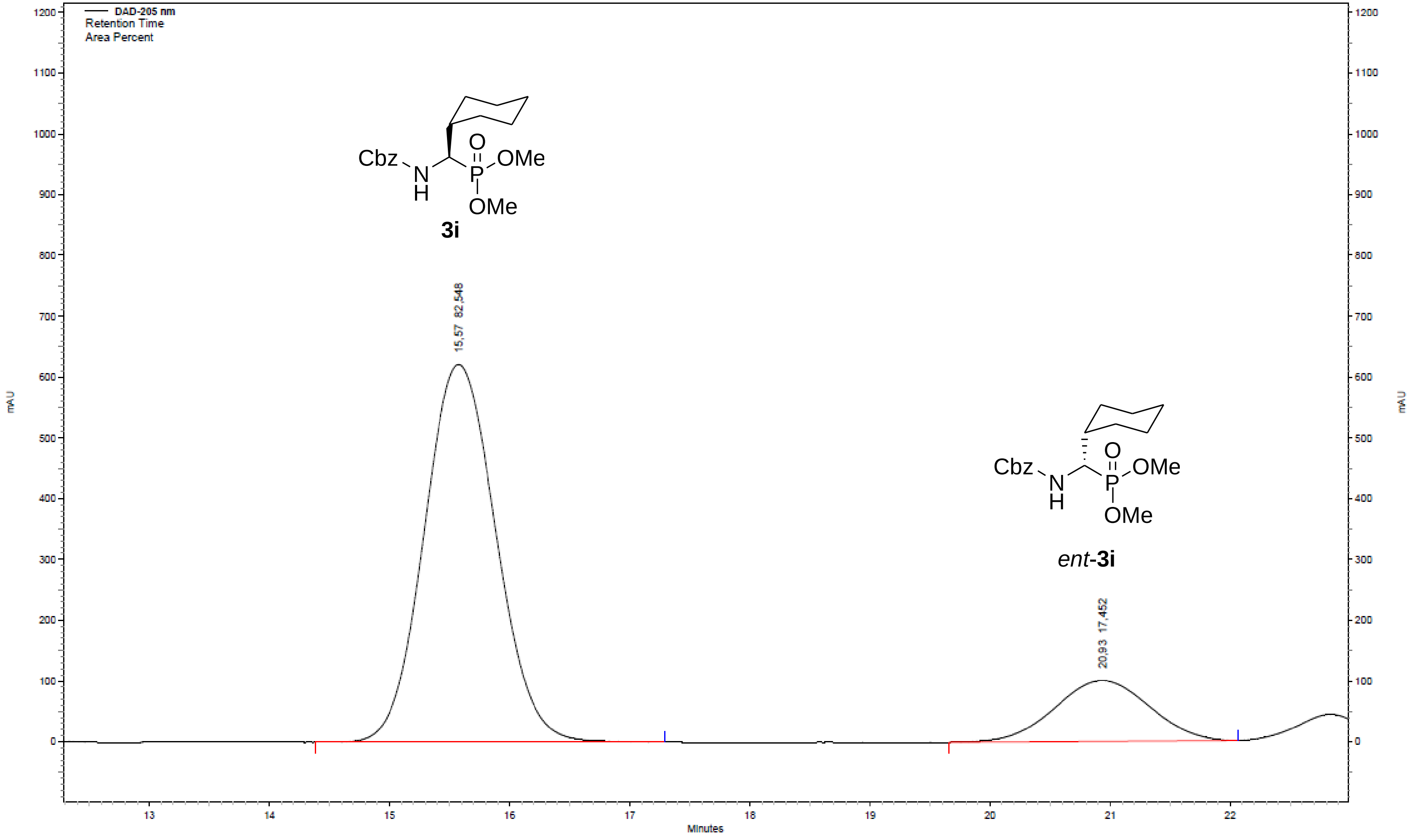

HPLC conditions:

Column: Chiralpak IA

Eluent: 15% *i*-PrOH/hexane

Flow: 0.75 mL/min

Detection: UV, 205 nm

HPLC results for the catalyst **5**		
Signal	Retention time	% Area
1	15.6	77.6
2	21.0	22.4

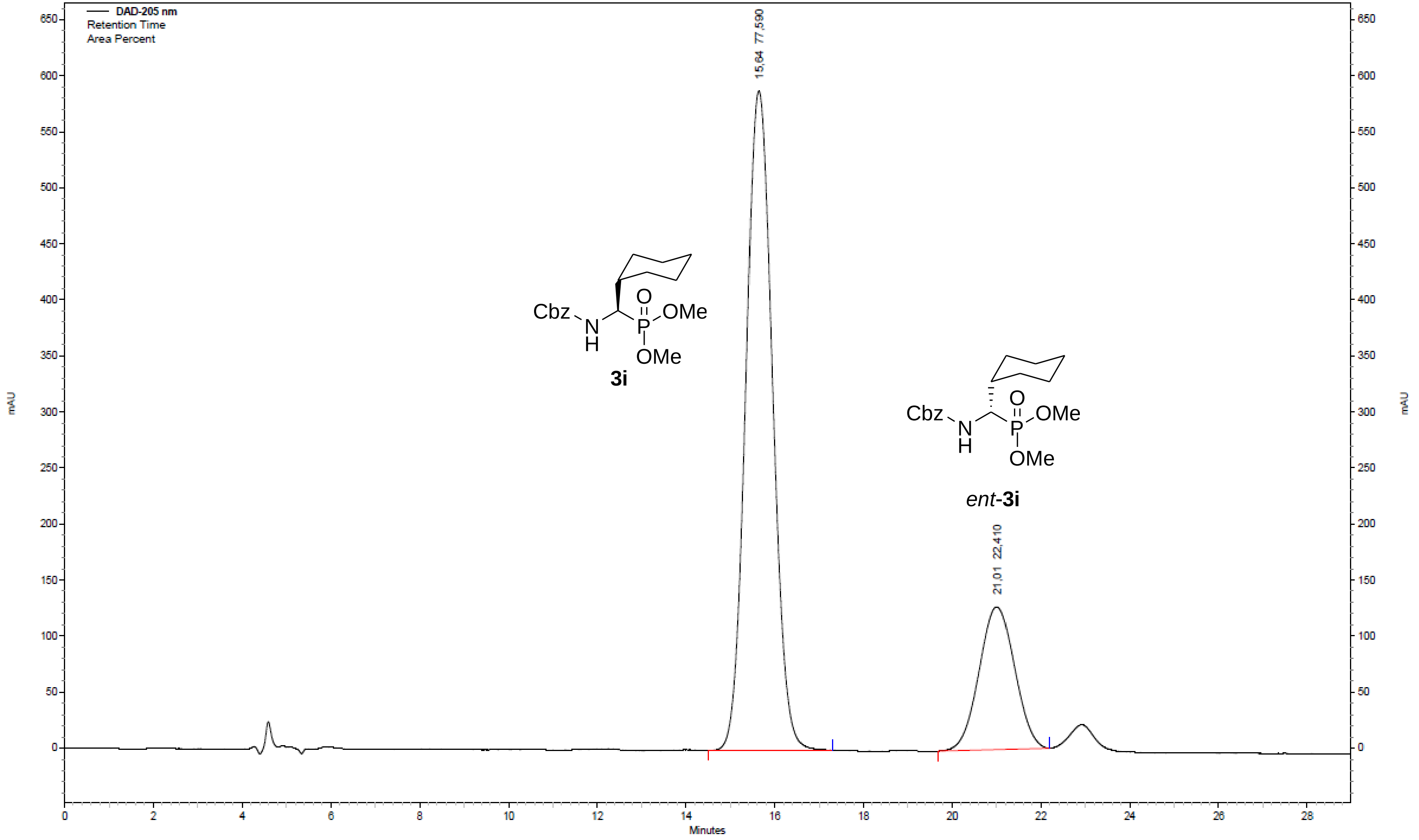

HPLC conditions:
Column: Chiralpak AD-H
Eluent: 10% *i*-PrOH/hexane
Flow: 0.75 mL/min
Detection: UV, 210 nm

Signal	Retention time	% Area
1	18.8	49.7
2	22.4	50.3

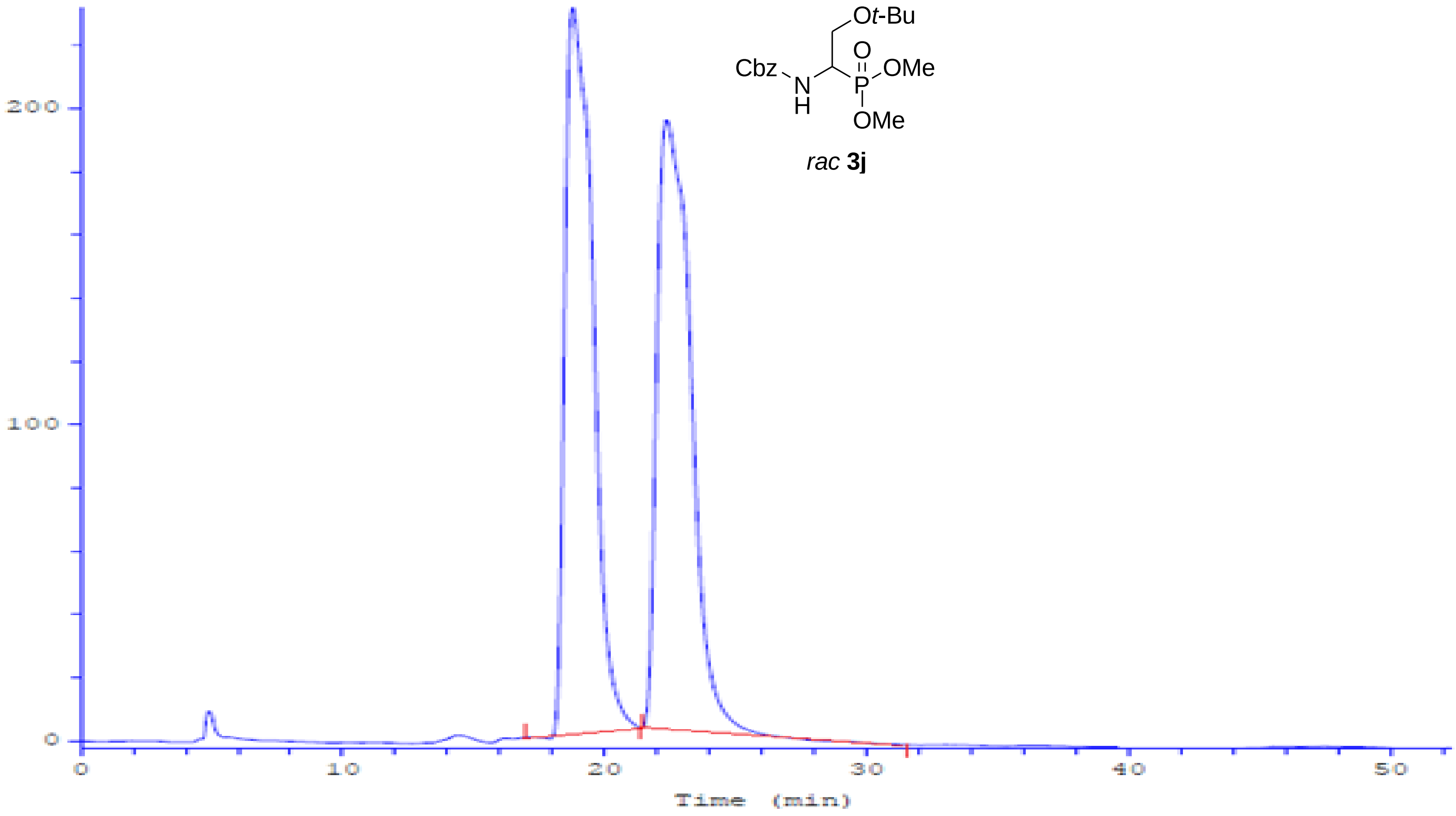

HPLC conditions:
Column: Chiralpak AD-H
Eluent: 10% *i*-PrOH/hexane
Flow: 0.75 mL/min
Detection: UV, 210 nm

HPLC results for the catalyst **4**		
Signal	Retention time	% Area
1	18.8	76.3
2	22.3	23.7

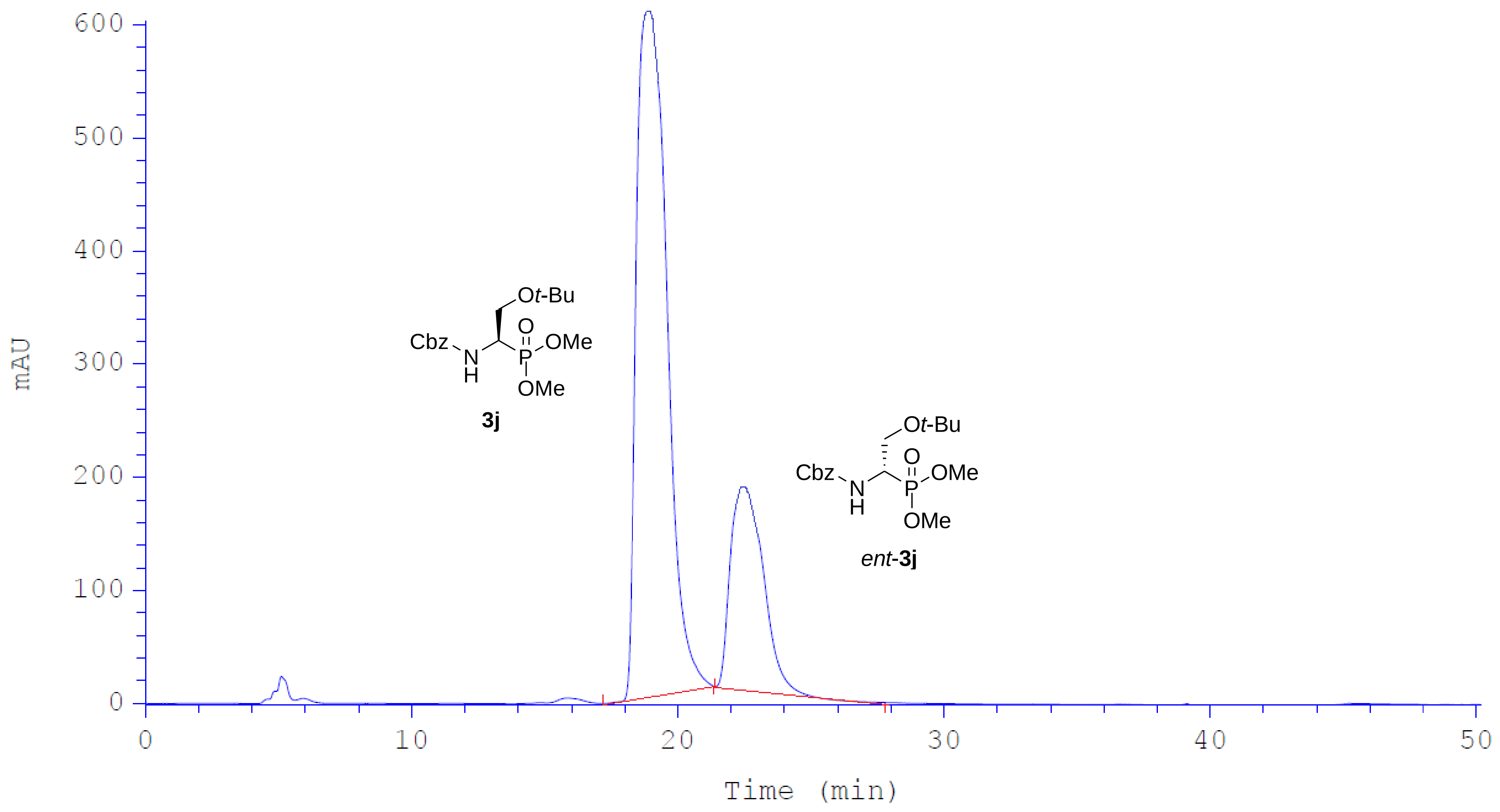

HPLC conditions:
Column: Chiralpak IA
Eluent: 10% *i*-PrOH/hexane
Flow: 0.75 mL/min
Detection: UV, 210 nm

Signal	Retention time	% Area
1	21.9	49.8
2	25.5	50.2

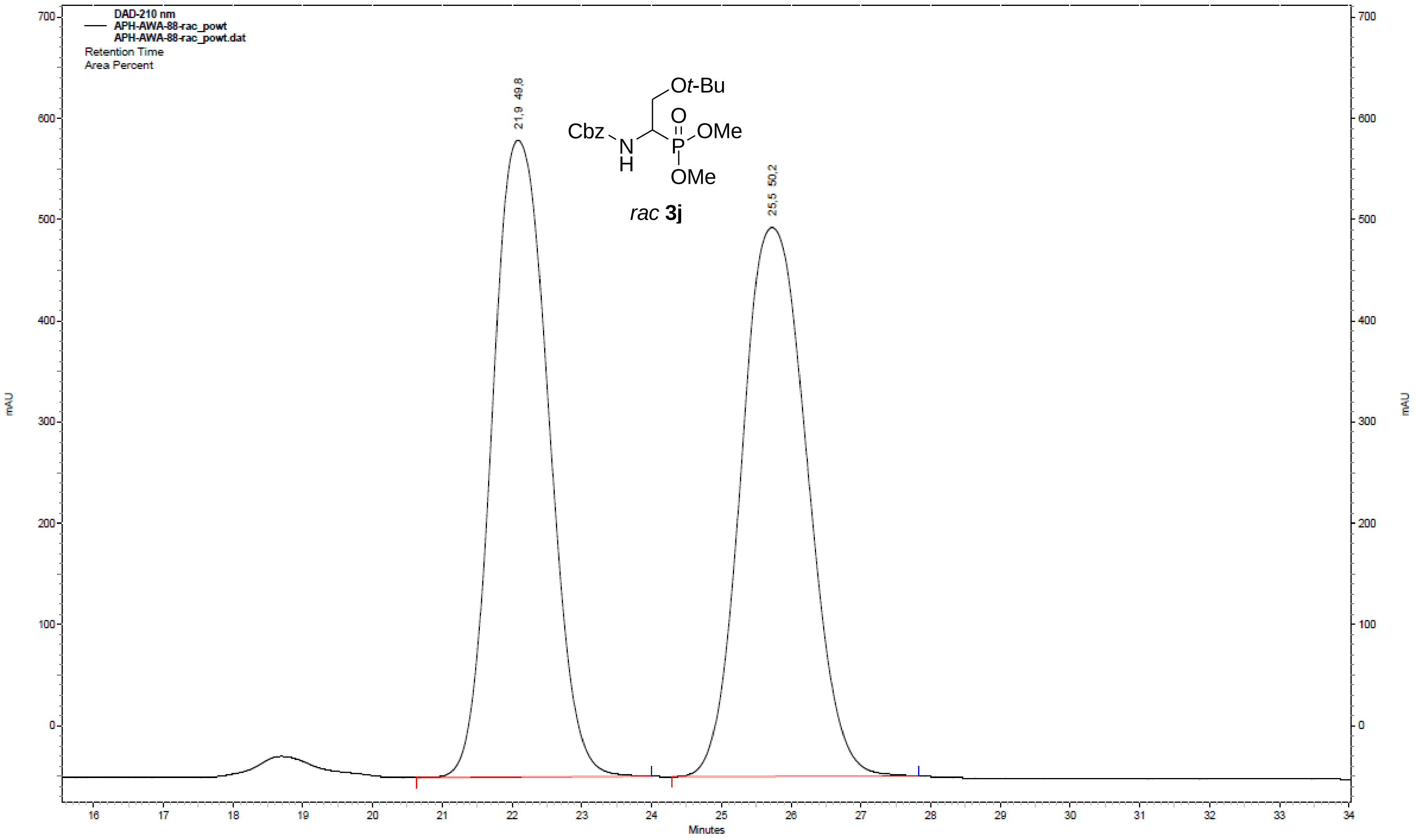

HPLC conditions:
Column: Chiralpak IA
Eluent: 10% *i*-PrOH/hexane
Flow: 0.75 mL/min
Detection: UV, 210 nm

HPLC results for the catalyst **5**		
Signal	Retention time	% Area
1	21.8	76.8
2	25.4	23.2

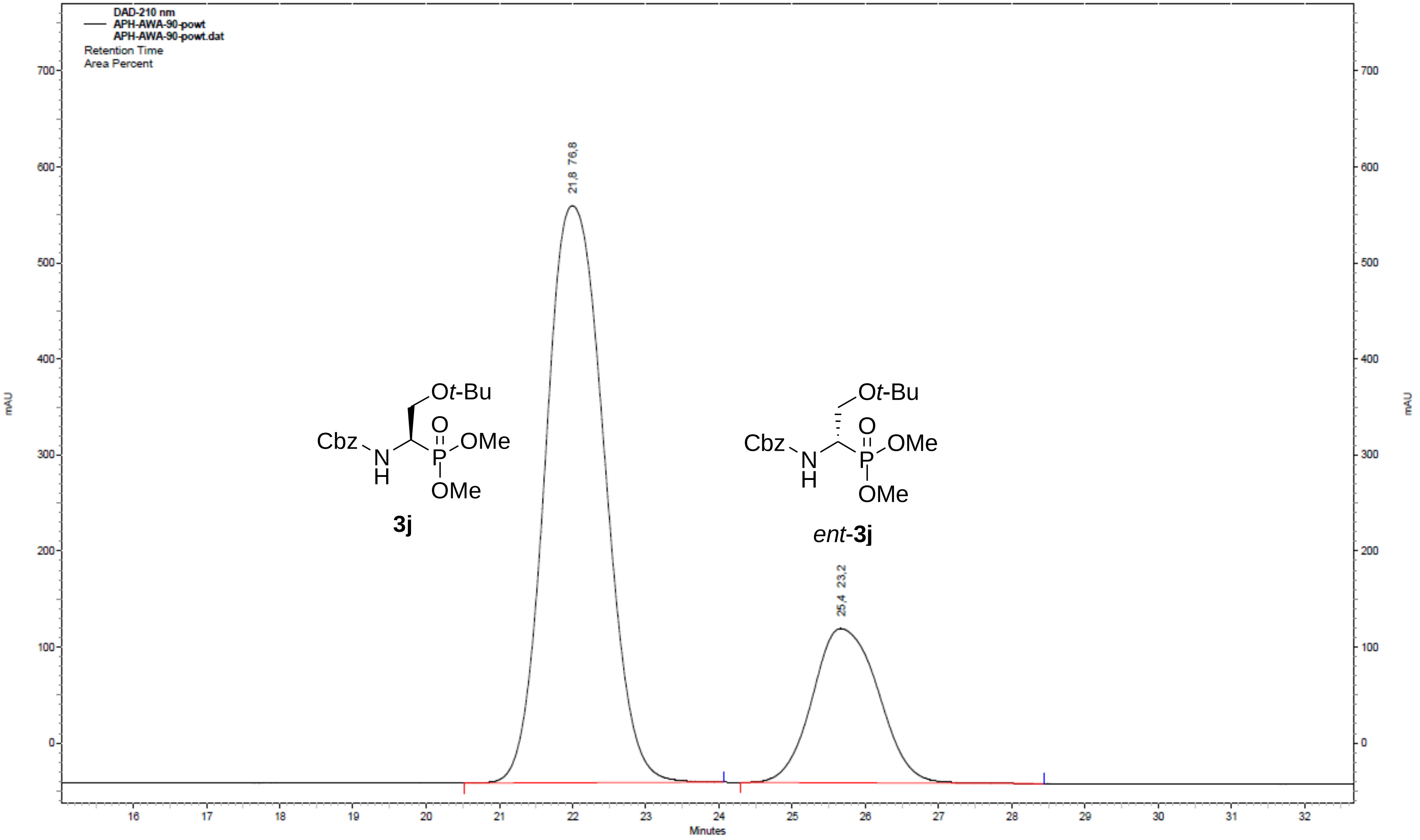

HPLC conditions:
Column: Chiralpak OD-H
Eluent: 3% *i*-PrOH/hexane
Flow: 0.5 mL/min
Detection: UV, 212 nm

Signal	Retention time	% Area
1	53.1	50.3
2	60.0	49.6

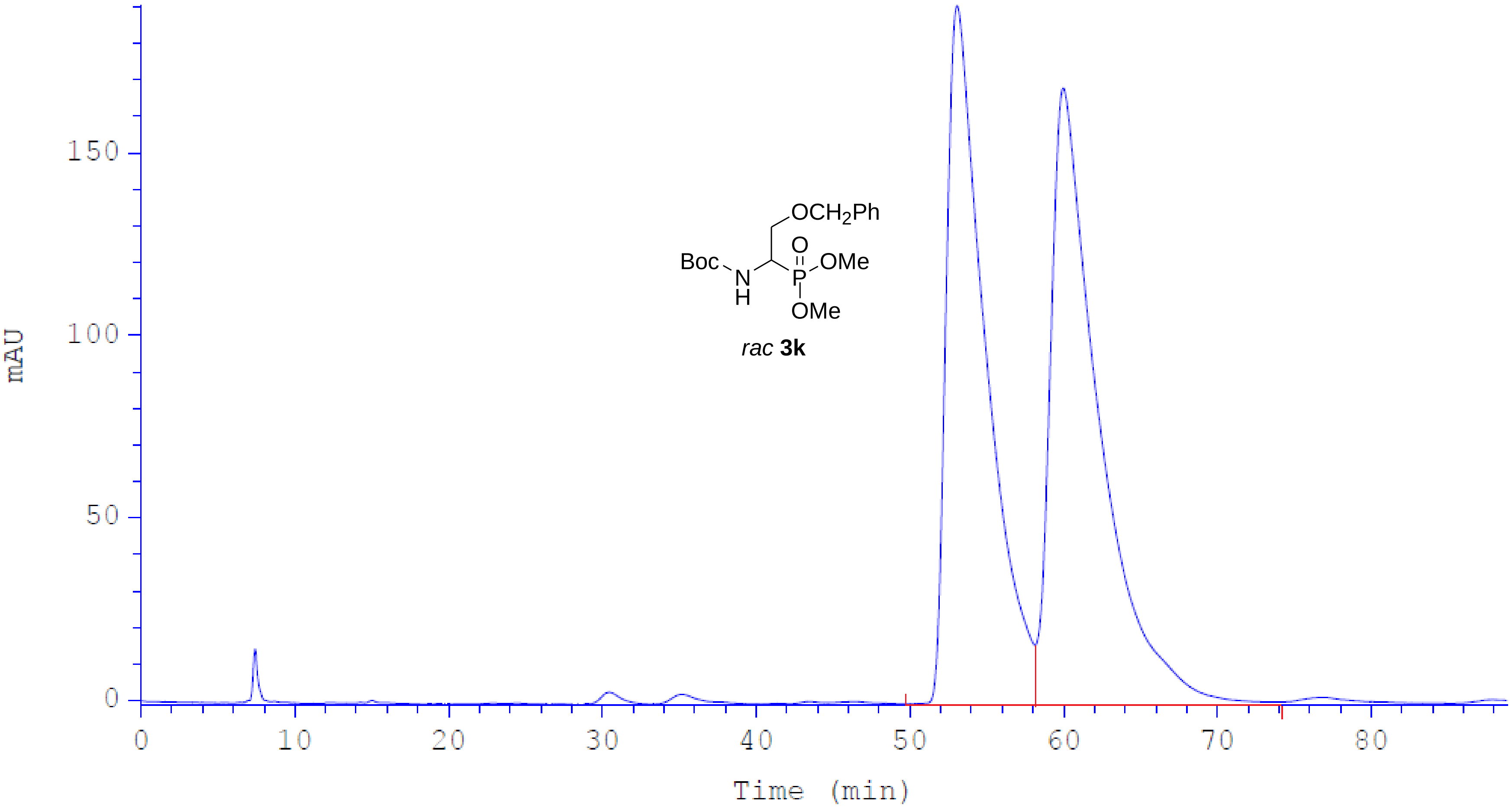

HPLC conditions:
Column: Chiralpak OD-H
Eluent: 3% *i*-PrOH/hexane
Flow: 0.5 mL/min
Detection: UV, 212 nm

HPLC results for the catalyst **5**		
Signal	Retention time	% Area
1	54.1	76.7
2	62.0	23.3

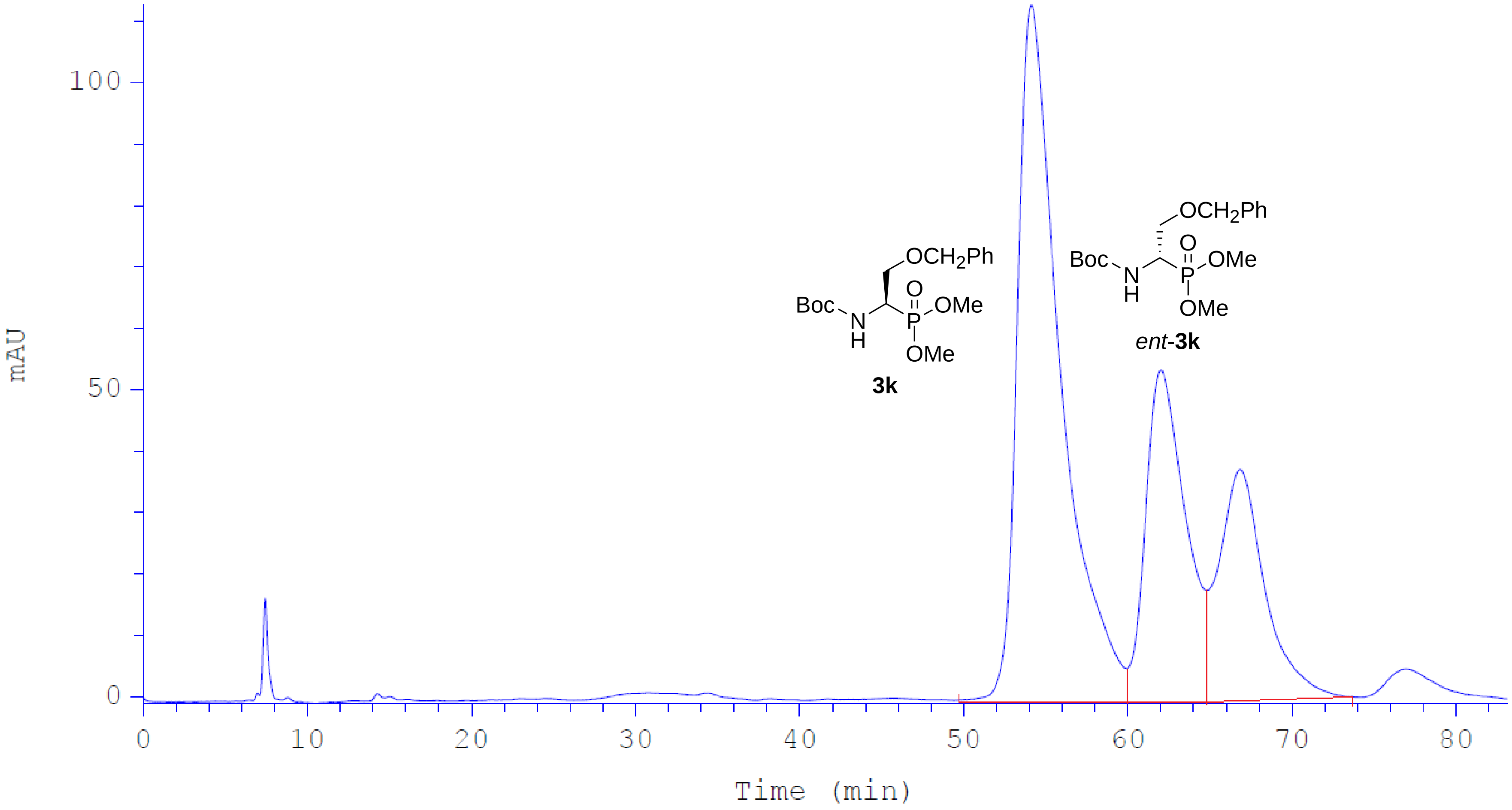

HPLC conditions:
Column: Chiralpak OD-H
Eluent: 3% *i*-PrOH/hexane
Flow: 0.5 mL/min
Detection: UV, 212 nm

HPLC results for the catalyst **4**		
Signal	Retention time	% Area
1	53.6	70.9
2	61.3	29.1

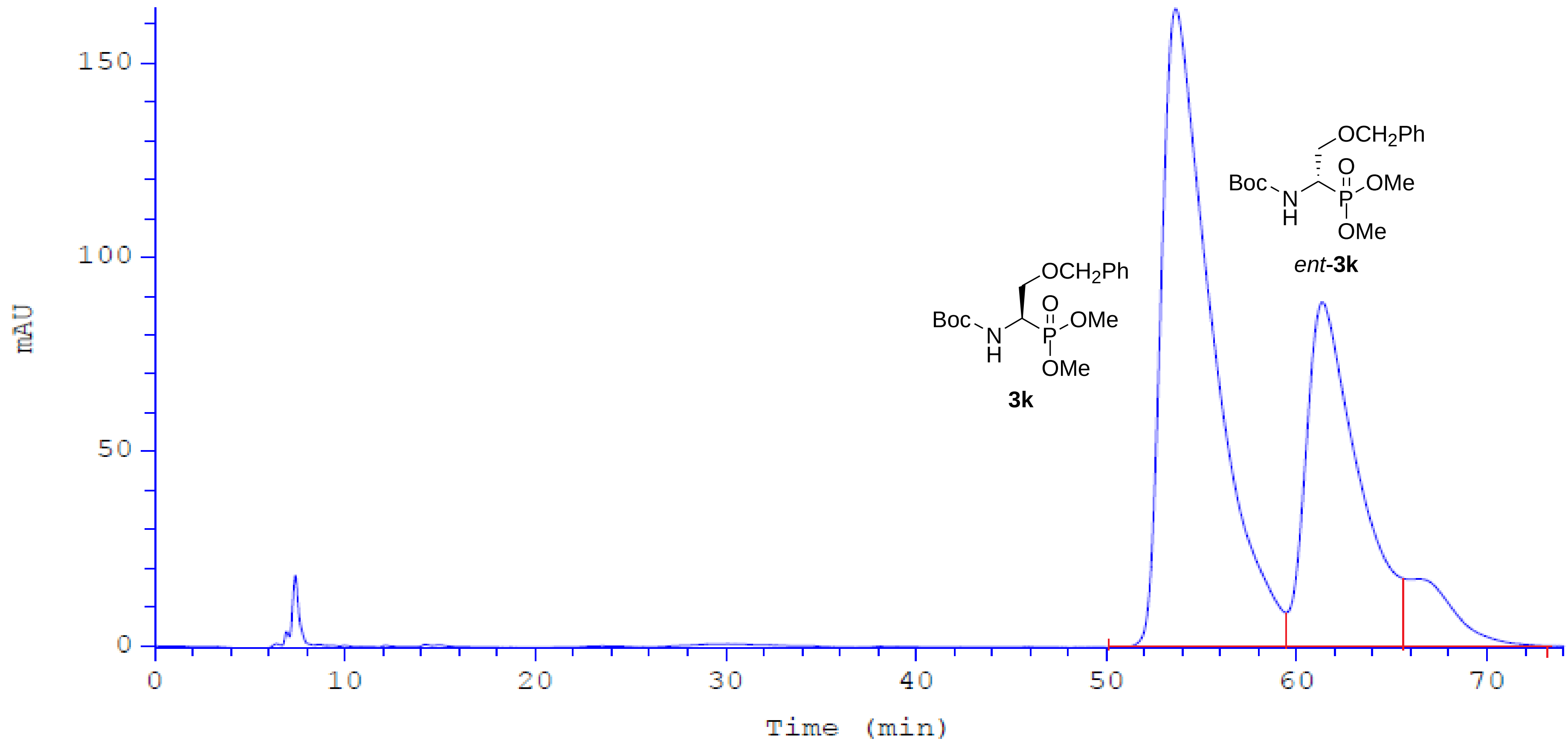

SDE test via flash chromatography

Table S1.

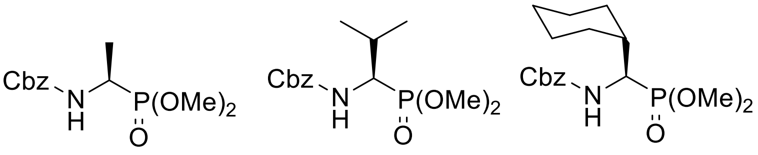

3a **3d** **3i**

Achiral flash chromatography on SiO_2; Eluent: hexane/EtOAc/acetone (5:3:2)

Product	*Starting*	*First fraction*	*Middle fraction*	*Last fraction*
**3a**	83% ee	83% ee	83% ee	83% ee
**3d**	56% ee	56% ee	56% ee	56% ee
**3i**	65% ee	65% ee	65% ee	65% ee

We conducted an appropriate SDE test for products **3a**, **3d** and **3i** via achiral flash chromatography. In the column chromatography process, we selected different fractions and measured the corresponding ee values. As shown in Table S1, the column chromatography doesn’t change the ee value of the products. HPLC results have been illustrated below.

HPLC conditions for compound 3a:
Column: Chiralpak AD-H
Eluent: 10% *i*-PrOH/hexane
Flow: 0.75 mL/min
Detection: UV, 210 nm

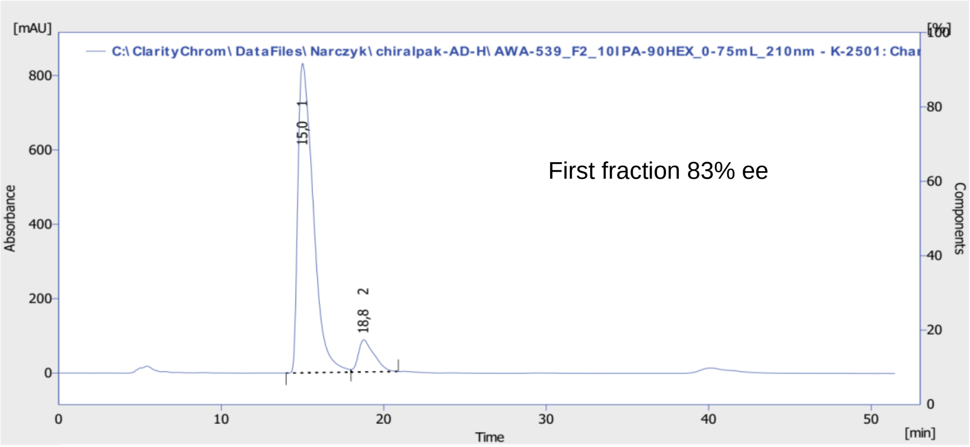

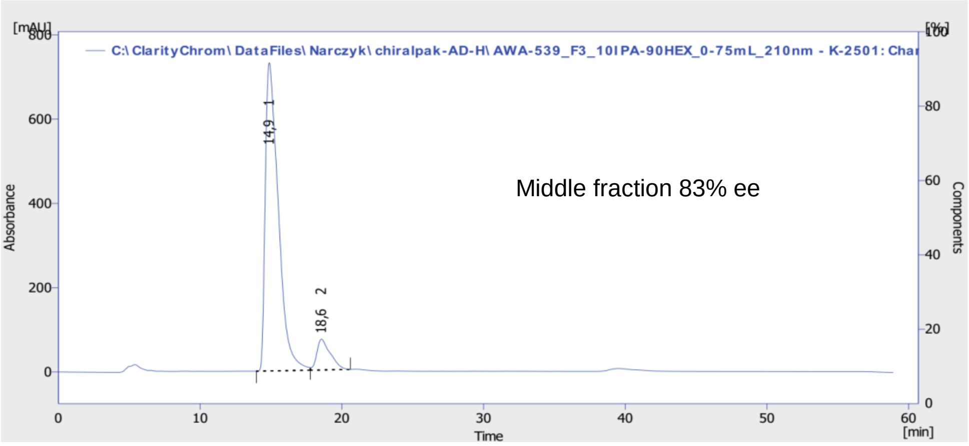

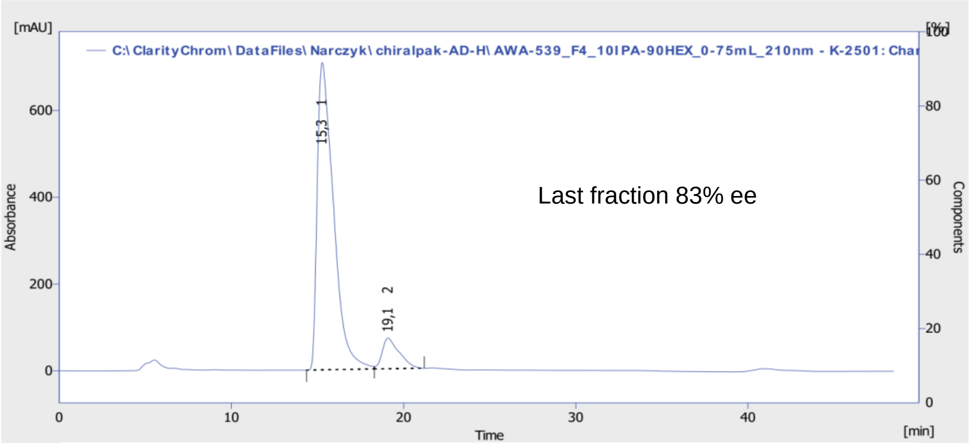

HPLC conditions for compound 3d:
Column: Chiralpak AD-H
Eluent: 10% *i*-PrOH/hexane
Flow: 0.75 mL/min
Detection: UV, 210 nm

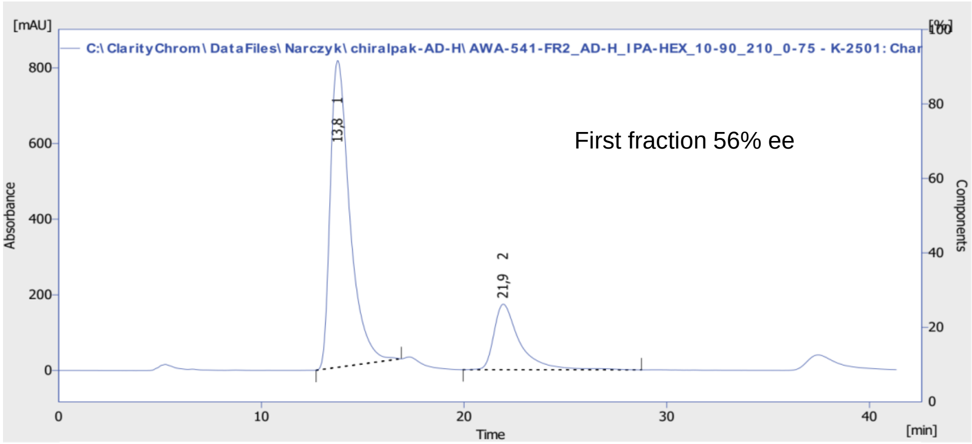

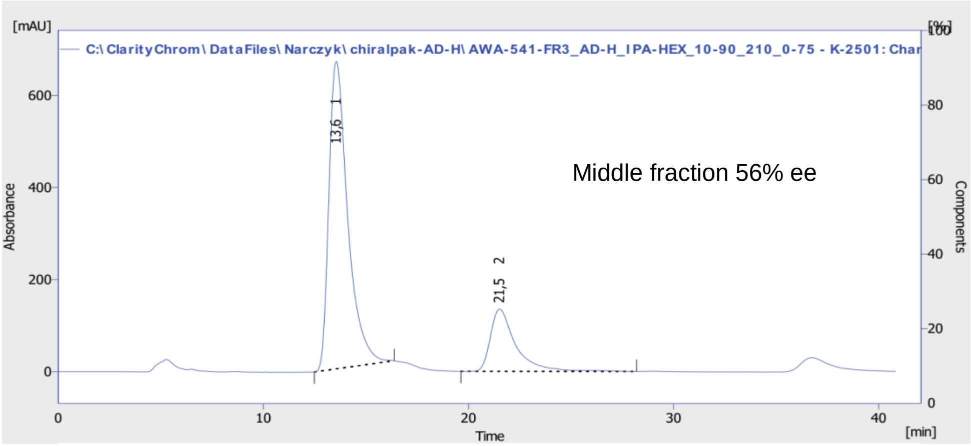

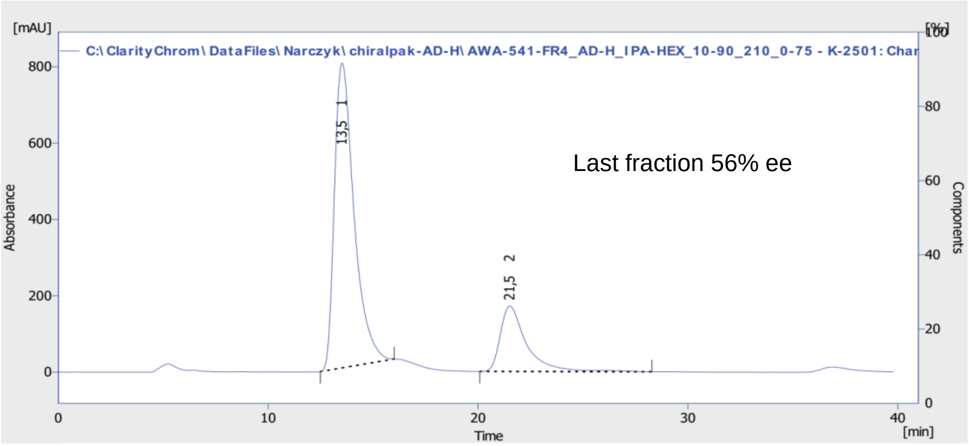

HPLC conditions for compound 3i:
Column: Chiralpak ADH
Eluent: 15% *i*-PrOH/hexane
Flow: 0.75 mL/min
Detection: UV, 205 nm

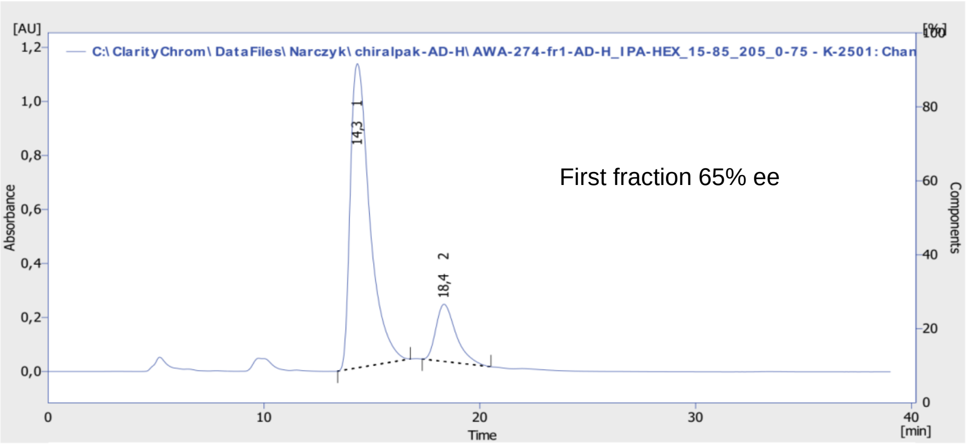

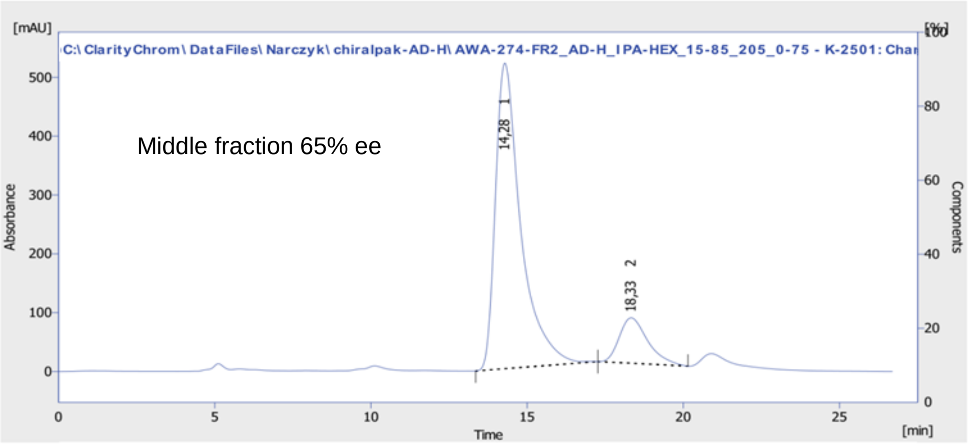

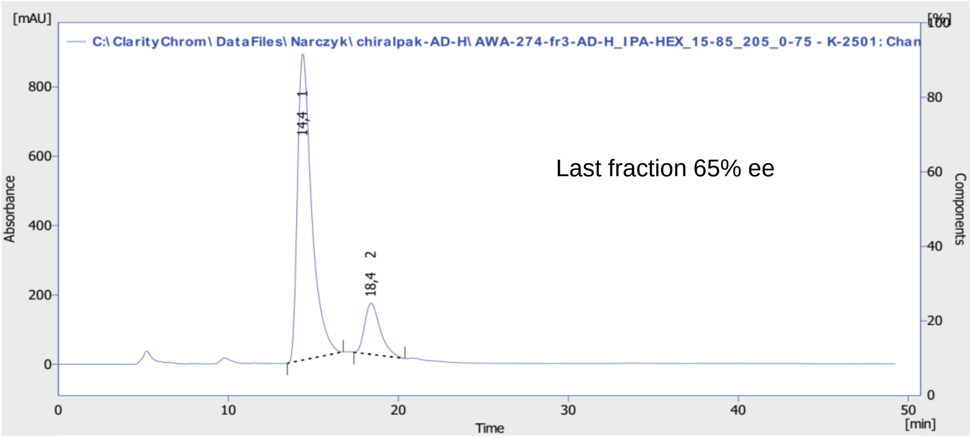

Supplement: Supplementary file 1 [file molecules-25-00405-s001.pdf]
